# Enteropathogenic *Escherichia coli* remodels host endosomes to promote endocytic turnover and breakdown of surface polarity

**DOI:** 10.1371/journal.ppat.1007851

**Published:** 2019-06-26

**Authors:** Ephrem G. Kassa, Efrat Zlotkin-Rivkin, Gil Friedman, Rachana P. Ramachandran, Naomi Melamed-Book, Aryeh M. Weiss, Michael Belenky, Dana Reichmann, William Breuer, Ritesh Ranjan Pal, Ilan Rosenshine, Lynne A. Lapierre, James R. Goldenring, Benjamin Aroeti

**Affiliations:** 1 Department of Cell and Developmental Biology, The Alexander Silberman Institute of Life Sciences, The Hebrew University of Jerusalem, Jerusalem, Israel; 2 Bio-imaging Unit, The Alexander Silberman Institute of Life Sciences, The Hebrew University of Jerusalem, Jerusalem, Israel; 3 Faculty of Engineering, Bar Ilan University, Ramat Gan, Israel; 4 Department of Biological Chemistry, The Alexander Silberman Institute of Life Sciences, The Hebrew University of Jerusalem, Jerusalem, Israel; 5 Proteomics and Mass Spectrometry Unit, The Alexander Silberman Institute of Life Sciences, The Hebrew University of Jerusalem, Jerusalem, Israel; 6 Department of Microbiology and Molecular Genetics, Institute for Medical Research Israel-Canada, The Hebrew University of Jerusalem, Jerusalem, Israel; 7 Department of Cell and Developmental Biology, Vanderbilt University School of Medicine, Nashville, Tennessee, United States of America; Emory University, UNITED STATES

## Abstract

Enteropathogenic *E*. *coli* (EPEC) is an extracellular diarrheagenic human pathogen which infects the apical plasma membrane of the small intestinal enterocytes. EPEC utilizes a type III secretion system to translocate bacterial effector proteins into its epithelial hosts. This activity, which subverts numerous signaling and membrane trafficking pathways in the infected cells, is thought to contribute to pathogen virulence. The molecular and cellular mechanisms underlying these events are not well understood. We investigated the mode by which EPEC effectors hijack endosomes to modulate endocytosis, recycling and transcytosis in epithelial host cells. To this end, we developed a flow cytometry-based assay and imaging techniques to track endosomal dynamics and membrane cargo trafficking in the infected cells. We show that type-III secreted components prompt the recruitment of clathrin (clathrin and AP2), early (Rab5a and EEA1) and recycling (Rab4a, Rab11a, Rab11b, FIP2, Myo5b) endocytic machineries to peripheral plasma membrane infection sites. Protein cargoes, e.g. transferrin receptors, β1 integrins and aquaporins, which exploit the endocytic pathways mediated by these machineries, were also found to be recruited to these sites. Moreover, the endosomes and cargo recruitment to infection sites correlated with an increase in cargo endocytic turnover (i.e. endocytosis and recycling) and transcytosis to the infected plasma membrane. The hijacking of endosomes and associated endocytic activities depended on the translocated EspF and Map effectors in non-polarized epithelial cells, and mostly on EspF in polarized epithelial cells. These data suggest a model whereby EPEC effectors hijack endosomal recycling mechanisms to mislocalize and concentrate host plasma membrane proteins in endosomes and in the apically infected plasma membrane. We hypothesize that these activities contribute to bacterial colonization and virulence.

## Author summary

Enteropathogenic *Escherichia coli* (EPEC) are pathogenic bacteria that cause infantile diarrhea. Upon ingestion, EPEC reaches the small intestine, where an injection device termed the type III secretion system is utilized to inject a set of effector proteins from the bacteria into the host cell. These proteins manipulate the localization and functions of host proteins, lipids and organelles and contribute to the emergence of the EPEC disease. The molecular mechanisms underlying the functions of the EPEC effector proteins are not completely understood. Here we show that early upon infection, two such effector proteins, EspF and Map, hijack host endosomes at bacterial adherence sites to facilitate endocytosis and recycling of plasma membrane proteins at these sites. The consequence of this event is the enrichment and mislocalization of host plasma membrane proteins at infection sites. One such protein is the transferrin receptor, which is a carrier for transferrin, whose function is to mediate cellular uptake of iron. Iron is a critical nutrient for bacterial growth and survival. We postulate that the unique manipulation of transferrin receptor endocytic membrane trafficking by EPEC plays an important role in its survival on the luminal surface of the intestinal epithelium.

## Introduction

Enteropathogenic *Escherichia coli* (EPEC) and enterohemorrhagic *Escherichia coli* (EHEC) are diarrheagenic extracellular pathogens affecting humans worldwide [1]. EPEC utilizes a molecular syringe termed type III secretion system (T3SS) to deliver a set of effector proteins from the bacterial cytoplasm into the host cell. The coordinated action of these effectors in space and time is critical for remodeling diverse host cell organelles and processes, e.g. the cytoskeleton, signaling pathways and intracellular trafficking, to support successful bacterial colonization and survival in the intestinal mucosa [2,3,4]. A prominent hallmark of EPEC infection is the induction of “attaching and effacing” (AE) lesions of the mucosal tissue, characterized by intimate microbial attachment to the apical plasma membrane of the infected epithelial cells, local elimination of brush border microvilli and the formation of a filamentous actin (F-actin)-rich membrane protrusion (often called pedestal) located immediately beneath the attached bacterium [5,6].

EPEC (strain O127:H6 E2348/69) translocates via its T3SS at least 21 ‘effector’ proteins into its host. These effectors are encoded by genes in the locus of enterocyte effacement (LEE) pathogenicity island, and by outside the LEE (non-LEE) genomic sites [7,8]. The first translocated effector is the intimin receptor (Tir) [9]. Upon translocation, Tir is incorporated into the host cell plasma membrane and its exposed extracellular domain binds the bacterial outer membrane protein, intimin. Tir-intimin interactions allow intimate attachment of the bacterium to its host plasma membrane and the formation of the F-actin rich pedestal [3]. Other injected effectors manipulate various processes in the host, including signaling pathways and immune response processes, cytoskeletal remodeling, the subversion of epithelial junctional components and membrane trafficking pathways [10,11,12]. Notably, however, limited information exists about the capacity of such effectors to manipulate membrane trafficking pathways of their host and, in particular, endosomal trafficking. Exploring the capacity of these effectors to hijack and manipulate endocytic trafficking pathways in a way that benefits the pathogen is important for better understanding the mechanisms by which EPEC colonizes its host.

Studies have shown that regulators of clathrin-mediated endocytosis (CME) are recruited to sites of EPEC infection. These include phosphatidylinositol 4,5-bisphosphate [PI(4,5)P_2_] [13], dynamin [14], the adaptor proteins Eps15 and epsin1 [15], Dab2 and Hip1R [16], CD2AP [17] and clathrin [18]. The recruitment of these components has been linked to the process of actin-rich pedestal formation. However, whether these recruitments are directly caused by injected effectors or a result of a broad host response to the bacterial assault is not known. The endocytic Rab5 [19], and the recycling VAMP3,Rab11 and Rab35 [20,21] protein markers have also been shown to be subverted by EPEC and EHEC. Nonetheless, neither study addressed the question of whether these endocytic regulators are recruited to infection sites and by what mechanism.

Data linking EPEC type III secreted effectors to endocytic trafficking have shown that the protein effector EspF transiently interacts with pre-existing clathrin-coated pits, and binds neuronal Wiskott-Aldrich syndrome protein (N-WASP) as well as the dynamin-associated PX-BAR domain sorting nexin 9 (SNX9) protein [22,23]. Recently, Tapia et al. have shown that EspF is involved in facilitating basolateral endocytosis and apical redistribution of the Crumbs polarity complex and Na+/K+ ATPase [19]. However, the mode by which EspF regulates these endocytic activities remains unknown. Here we show that early upon EPEC microcolony attachment to non-polarized host cells, EspF and Map prompt the recruitment of transferrin receptors (TfnR) and Rab11a-positive recycling endosomes to peripheral infection sites. This event may contribute to an increase in TfnR endocytosis and recycling (i.e. endocytic turnover) at these sites. We identified three novel EspF binding proteins, WIPF1, SNX18 and SNX33, which together with N-WASP and SNX9 could contribute to endocytic remodeling of the host. In polarized cells, we show that EspF is sufficient to promote basolateral-to-apical transcytosis of TfnR and increase in apical endocytic turnover. Our results tie for the first time the activity of an EPEC effector protein to endosomal dynamics and breakdown of surface polarity of infected cells. This event could lead to enrichment of the apical infected plasma membrane and endosomes with basolateral membrane proteins that contribute to bacterial colonization and the EPEC disease.

## Results

### T3SS-dependent recruitment of early and recycling endocytic elements, and Tfn-TfnR, to apical infection sites

Previous studies have shown that EPEC and EHEC subvert various elements of the clathrin-coated pits endocytic and recycling machineries [13,14,15,17,18,19,20,21,24]. However, the extent to which T3SS elements are involved in this event has been sparsely studied. We therefore investigated the T3SS dependence of the distribution of clathrin endocytic (CHC, AP2-α, Rab5a and EEA1) and recycling [Rab11a, Rab11b, Rab25, Myosin 5b (Myo5b)] components upon EPEC infection of polarized MDCK cells. Results show that all these markers accumulated at EPEC-*wt*, and not at EPEC-*escV* infection sites (S1 Fig). These data suggest the involvement of type III secreted elements in the recruitment of early and recycling endocytic components to bacterial infection sites at the apical plasma membrane.

Transferrin receptors (TfnR) are basolateral recycling membrane proteins that typically do not reach the sub-apical Rab11a/Rab25 endosomal recycling compartment (reviewed in [25]) unless they are enforced to undergo transcytosis [26]. We analyzed whether the distribution of TfnR and their Tfn cargo is altered in response to EPEC infection of polarized MDCK-PTR9 (S2A Fig), Caco2-BBe (S2B Fig), or MDCK-GFP-TfnR (S3A Fig) cells. Fluorescently-tagged Tfn was internalized either from the apical, or from the basolateral (basal) plasma membrane of the polarized cells concomitant to apical exposure to EPEC-*wt* or EPEC-*escV*. The ligand distribution along the basal-apical axis of the cells was analyzed in fixed (MDCK-PTR9 and Caco2-BBe), and live (MDCK-GFP-TfnR) cells by confocal microscopy. Tfn internalized from either cell pole was found to be clustered at EPEC-*wt*, but not at EPEC-*escV*, infection sites. Surface immunostained TfnR (s-TfnR) also showed significant clustering at EPEC-*wt*, compared to EPEC-*escV* infected cells. Interestingly, SH3BP4 and ACAP1, which have been shown to promote TfnR endocytosis and recycling by binding to the receptor [27,28], were also recruited to infection sites in a T3SS-dependent manner (S3B Fig). These data suggest that T3SS elements prompt the clustering of Tfn-TfnR complexes and proteins that directly modulate their endocytic/recycling pathways at apical infection sites.

#### Basolaterally internalized Tfn partially co-localized with Rab11a/Myo5b at apical infection sites and undergoes transcytosis

**Fig 1:**
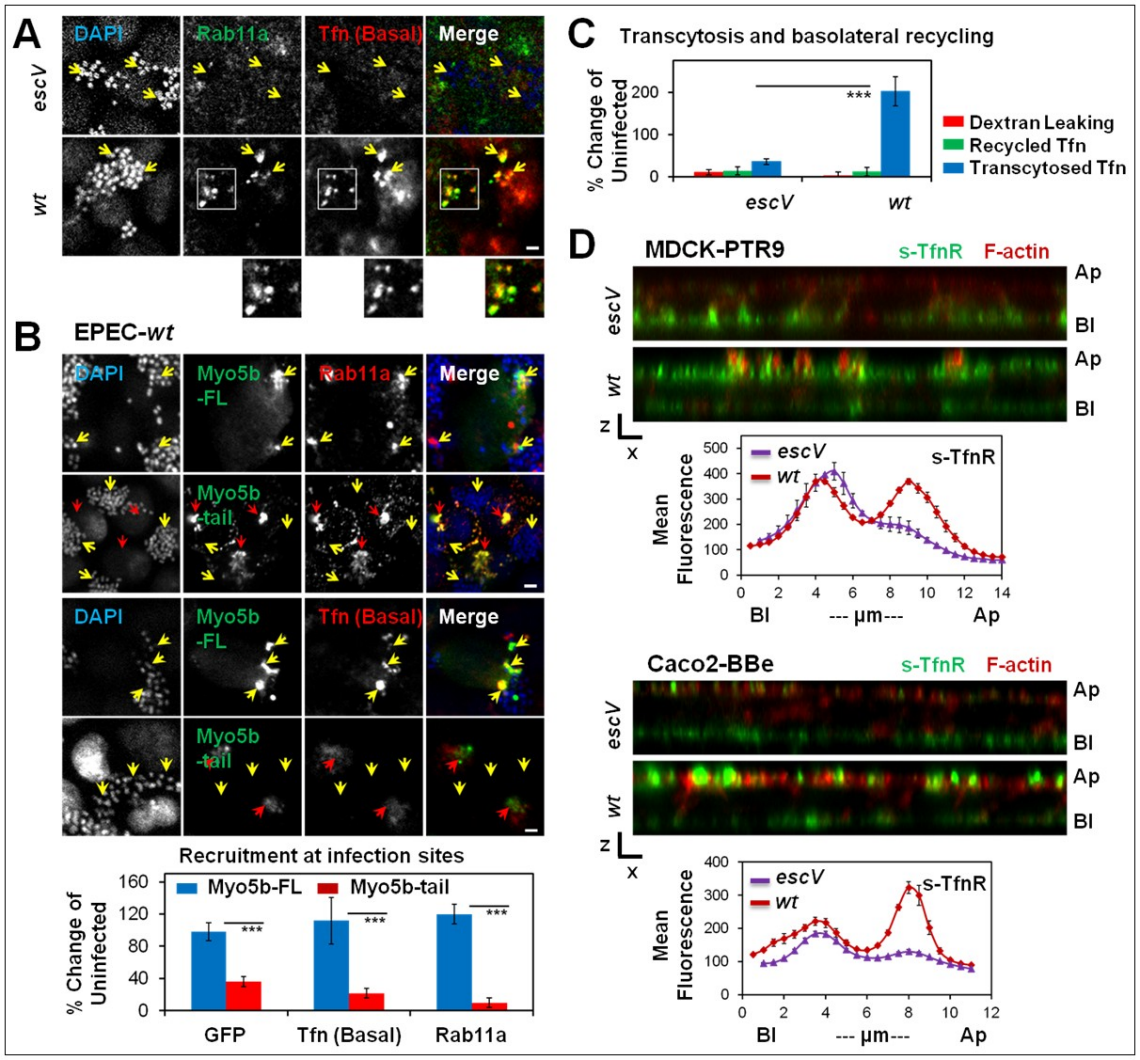
T3SS-dependent targeting of TfnR to the apically infected plasma membrane. **(A) Basolaterally internalized Tfn and Rab11a partially co-localize at apical infection sites**. Polarized MDCK-PTR9 cells were infected with EPEC-*escV*, or EPEC-*wt* and exposed to basolateral Tfn-AF647 [Tfn (Basal)]. Cells were then fixed and immunolabeled with anti-Rab11a antibodies. Representative confocal images are shown. Boxed regions highlight areas of Tfn and Rab11a co-residence at apical infection sites. Arrows point towards additional regions where Rab11a, or Tfn, are visualized at infection sites. Bar = 5 μm. **(B) Rab11a and Tfn recruitment at apical infection sites depends on functional Myo5b motors**. Polarized MDCK-PTR9 cells expressing the GFP- Myo5b-FL or GFP-Myo5b-tail mutant were infected with EPEC-*wt*. These cells were exposed to basolateral Tfn-AF647 [Tfn (Basal)], fixed, immunolabeled with anti-Rab11a antibodies, and stained with DAPI. Representative confocal images and recruitment of Rab11a, Myo5b and Tfn at infection sites are shown. Yellow arrows point towards infecting microcolonies. Red arrows point towards Myo5b-tail labeled structures. Bar = 5 μm. **(C) T3SS-dependent increase in Tfn transcytosis**. Polarized MDCK-PTR9 cells were infected with EPEC-*escV*, EPEC-*wt*, or left uninfected. Tfn basolateral-to-apical transcytosis and recycling were measured. Results are mean ± SE of n≥12 samples analyzed in four independent experiments. **(D) T3SS-dependent increase in the abundance of s-TfnR on the apical surface**. Polarized MDCK-PTR9 (top panel) and caco2-BBe (bottom panel) cells were infected with EPEC-*escV*, or *wt*, fixed and TfnR located at the apical (Ap) and basolateral (Bl) surfaces (s-TfnR) were immunostained with the B3/25 antibodies. Cells were then permeabilized, stained with TR-phalloidin (F-actin) and imaged by confocal microscopy. x-z representative images and the apical/basal distribution of s-TfnR (Z-plots) are shown. Results are mean ± SE of 12 images analyzed in three independent experiments. Bar = 5 μm.

To investigate further the characteristics of apically recruited Tfn/TfnR, we examined the localization of basolaterally internalized Tfn with respect to the Rab11a, a GTPase suggested to be involved in transcytosis. Confocal imaging revealed that the basolaterally internalized Tfn clustered at apical infection sites in a T3SS dependent manner and that the Tfn apical clusters partially co-localized with Rab11a (Fig 1A) and with the full-length (FL) GFP-Myo5b (Myo5b-FL) motor that moves Rab11a-positive vesicles along actin filaments (Fig 1B). These data suggest that a fraction of the basolaterally recycling ligand has possibly reached the transcytotic route mediated by the Rab11-Myo5b positive apical recycling endosomes [25,29].

In polarized MDCK cells, the Myo5b motor has been suggested to play a role in transcytosis, but not in basolateral recycling [30]. Ectopically expressed Myo5b-FL co-clustered with Rab11a and basolaterally internalized Tfn at apical bacterial infection sites (Fig 1B). These data further support the possibility that Tfn internalized from the basolateral pole accessed the Rab11-Myo5b positive apical recycling endosomes. Interestingly, ectopically expressed GFP-Myo5b-tail (Myo5b-tail) appeared as aggregates colocalizing with Rab11a, but not with basolaterally internalized Tfn (Fig 1B; **red arrows**). Expression of Myo5b-tail inhibited the recruitment of Rab11a and Tfn to the infection sites (Fig 1B, **yellow arrows**), suggesting that Myo5b is involved in the trafficking of basolaterally internalized TfnR to the apical recycling endosomes at the infection sites. These findings show that Myo5b-tail was able to inhibit Tfn recruitment at the infection site without sequestering it at the Myo5b aggregates. This outcome can be explained in light of previous studies suggesting that Myo5b motors can act on basolateral recycling endosomes by sequestering Rab11-family interacting proteins, e.g. FIP2 [31], which are part of the Rab11-FIP2-Myo5b complex [32].

Data presented in Fig 1C show that basolaterally internalized Tfn was released at a greater extent into the apical medium of EPEC-*wt*, compared to EPEC-*escV* infected cells. TR-Dextran (70 kDa) added to the apical medium showed negligible release to the basolateral medium in the infected cells, suggesting that the epithelial barrier remained intact under the experimental conditions employed. Taken together, these data suggest that type III secreted elements evoke basolateral-to-apical transcytosis of TfnR, a process that can contribute to the increased steady-state apical localization of s-TfnR in EPEC-*wt* infected cells (Fig 1D).

### EPEC promotes type III secretion-dependent increase in apical endocytic turnover

**Fig 2:**
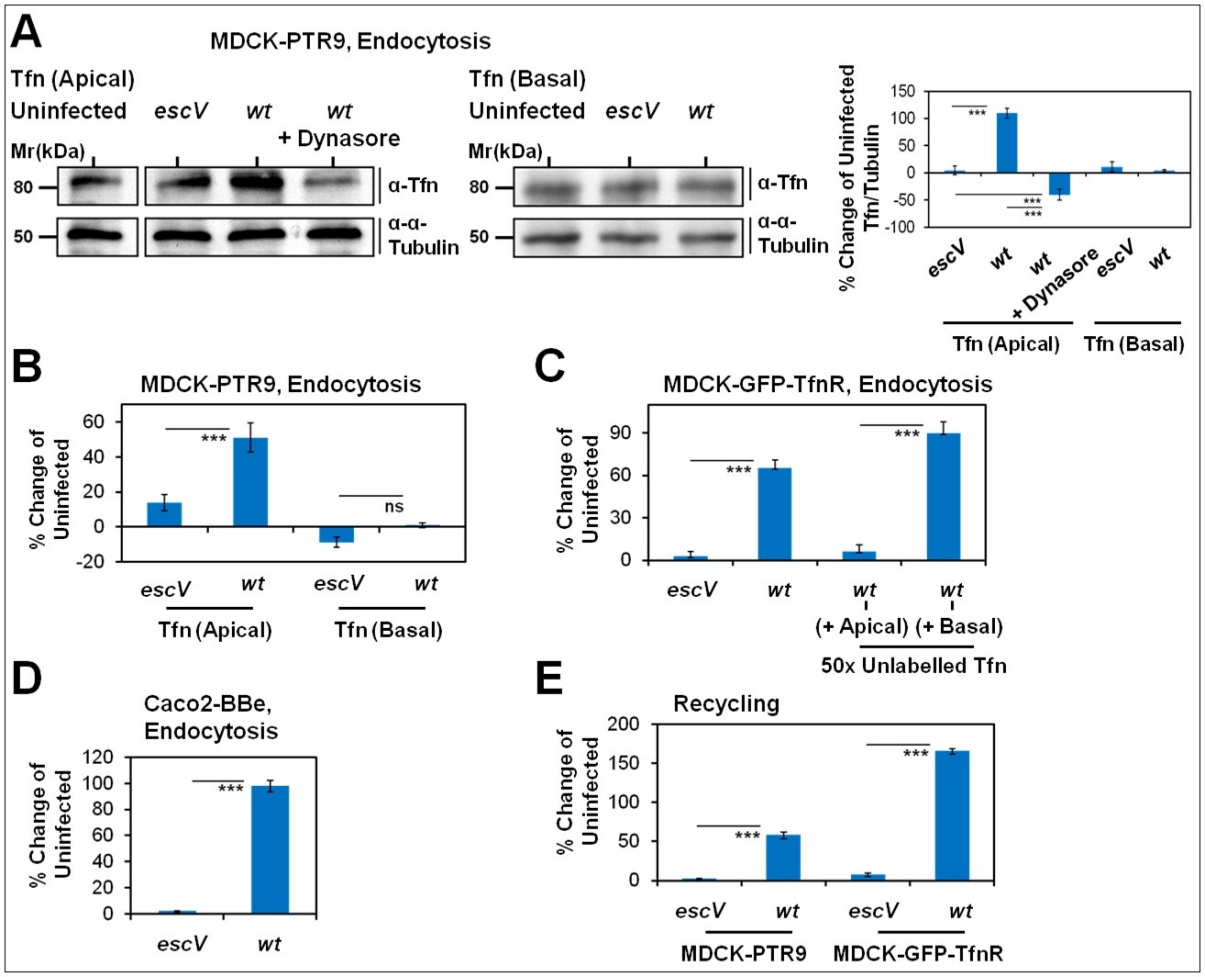
T3SS elements mediate an increase in apical endocytic turnover. **(A-D) EPEC-*wt* promotes an increase in Tfn apical endocytosis. (A) Biochemical analysis**: Polarized MDCK-PTR9 cells were infected with EPEC-*escV*, *wt*, or left uninfected. Cells were exposed to human holo-Tfn (S2 Table) administered at either their apical [Tfn (apical)] or basolateral [Tfn (Basal)] surface. In another experiment, the dynamin inhibitor, Dynasore, was supplemented with the apical Tfn to the cells. Cells were lysed and cell-associated Tfn was detected by Western blotting, using anti-human Tfn antibodies (S4 Table). Representative Western blots are shown. For the quantitative analysis, cell-associated Tfn was normalized to α-tubulin, and results are presented as “% change of uninfected” cells. Results are mean ± SE of three independent experiments. **(B) FACS analysis**: Polarized MDCK-PTR9 cells were infected and exposed to Tfn-AF647, as above. The amount of cell-associated ligand was analyzed by the ‘endocytosis assay’ using flow cytometry. Results are mean ± SE; n≥12 were analyzed in three independent experiments. **(C) EPEC-*wt* specifically affects Tfn endocytosis**. Polarized MDCK-GFP-TfnR cells were infected and exposed to apical Tfn-AF647 [Tfn (apical)], as above. One set of cells was supplemented with a 50x molar excess of unlabeled Tfn administered to the apical surface while another set of cells was exposed to a 50x molar excess of unlabeled Tfn administered to the basal surface. Cell-associated ligand was assessed by the ‘endocytosis assay’ using flow cytometry. Data are presented as "% change of uninfected" cells. Results are mean ± SE; n≥6 were analyzed in two independent experiments. **(D) T3SS-dependent increase in apical endocytosis of Tfn in Caco2-BBe cells**. Polarized Caco2-BBe cells were infected with EPEC-*escV*, *wt*, or left uninfected. Cells were then exposed to apical Tfn-AF647, and cell-associated ligand was assessed by flow cytometry. Results are mean ± SE; n≥12 were analyzed in three independent experiments. **(E) T3SS-dependent increase in apical recycling of Tfn**. Polarized MDCK-PTR9 or MDCK-GFP-TfnR cells were infected with EPEC-*escV*, *wt*, or left uninfected. Cells were then exposed to apical Tfn-AF647 and the levels of released Tfn were determined by the ‘recycling assay’, using flow cytometry. Results are mean ± SE; n = 12 were analyzed in three independent experiments.

The observation that early endocytic and recycling machineries are recruited to infection sites has raised the hypothesis that EPEC modulates them to control membrane trafficking at the infected plasma membrane. To address this hypothesis, we examined whether EPEC infected cells display altered Tfn internalization and recycling capacities. To this end, MDCK-PTR9 cells were infected and concomitantly exposed to Tfn administered at either the apical or the basolateral poles of the cells. In the case of apically internalized Tfn, one set of EPEC-*wt* infected cells was treated with the dynamin inhibitor Dynasore. Cells were then washed, lysed and the amount of cell-associated Tfn was analyzed by Western blotting. Data showed an apparent increase in cell-associated Tfn only when the ligand was administered to the apical surface of EPEC-*wt* infected cells. The apical intake of Tfn by the Dynasore treated cells was significantly lower compared to EPEC-*wt* infected cells and uninfected cells (Fig 2A). These data suggest that type III secreted elements stimulate dynamin-dependent apical endocytosis of Tfn.

To further validate this conclusion, we applied a flow-cytometry-based assay for monitoring Tfn endocytosis and recycling. Polarized MDCK-PTR9 cells were infected with EPEC-*wt*, or EPEC-*escV*, or left uninfected, in the presence of fluorescently-tagged Tfn applied at either the apical, or the basolateral (basal) poles of the cells. Thereafter, adherent cells were detached from their substrate and cell-associated Tfn was determined by flow cytometry. Results showed an apparent increase in cell-associated Tfn only in EPEC-*wt* infected cells that were exposed to apical Tfn (Fig 2B). The EPEC-*wt* driven increase in Tfn endocytosis was also observed in polarized MDCK-GFP-TfnR cells (Fig 2C), and in Caco2-BBe cells, which express endogenously the hTfnR (Fig 2D). The intake of fluorescently labeled Tfn by EPEC-*wt* infected cells was diminished when an excess of unlabeled Tfn was co-administered with Tfn-AF647 to the apical plasma membrane of the cells (Fig 2C). In contrast, the addition of unlabeled Tfn to the basolateral compartment had no effect on the EPEC-induced intake of Tfn-AF647 added to the apical plasma membrane (Fig 2C). These data further argue that Tfn is specifically internalized from the infected apical plasma membrane. Finally, we used flow cytometry to monitor the effects that EPEC infection may have on the release (i.e. apical recycling) of apically internalized Tfn. Results showed enhanced Tfn release in response to EPEC-*wt* infection, compared to EPEC-*escV* and uninfected cells (Fig 2E). Together, these data suggest that type III secreted components stimulate apical endocytosis and recycling of Tfn, i.e. increase the apical endocytic turnover of the ligand.

### EPEC elicits an increase in Tfn endocytic turnover in non-polarized HeLa cells

**Fig 3:**
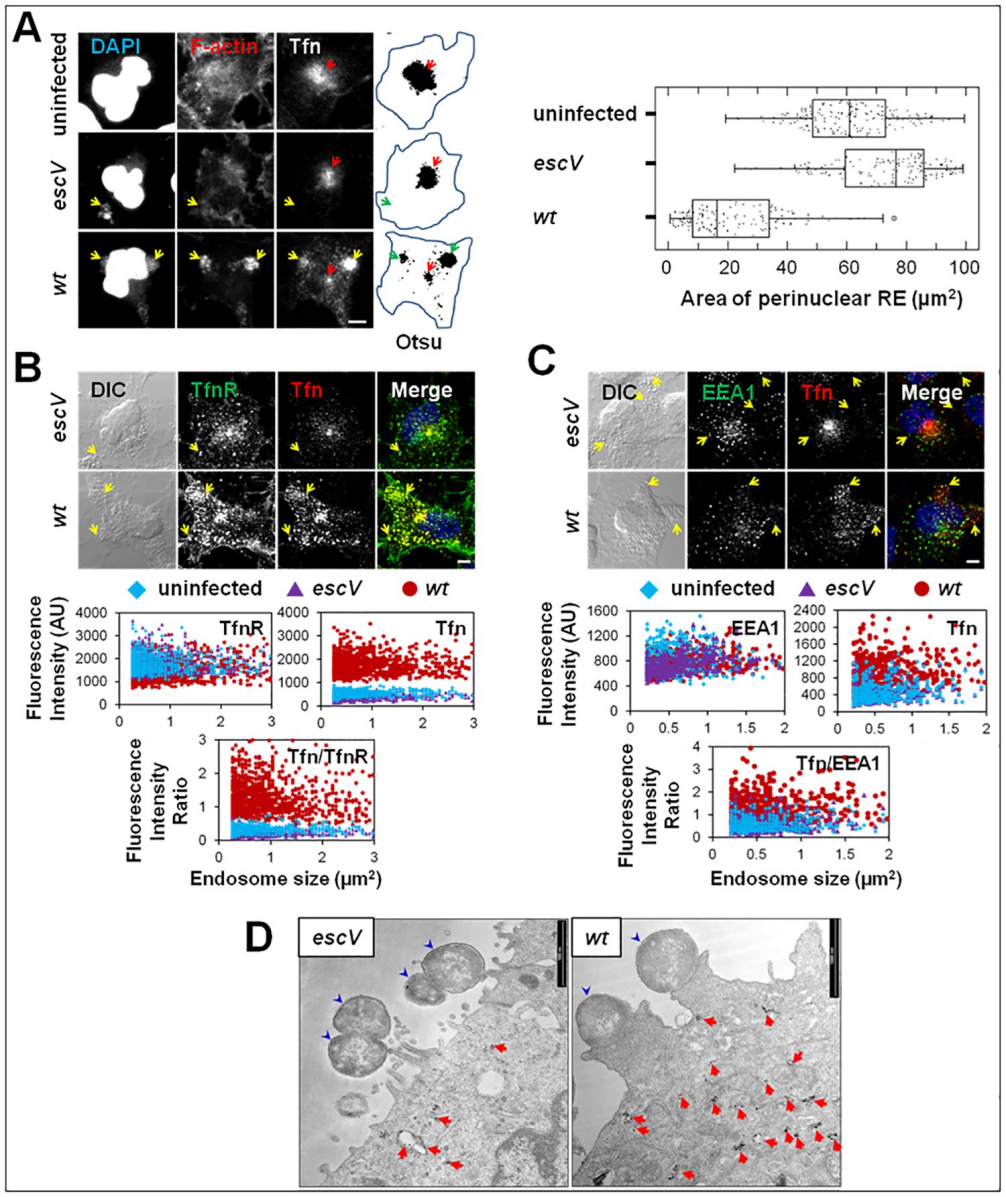
T3SS secreted elements redistribute endosomes from perinuclear to peripheral host sites. **(A). T3SS elements promote a reduction in the Tfn-positive perinuclear recycling endosome area and an increase in endosomal abundance at peripheral infection sites**. HeLa cells were infected with EPEC-*escV*, or EPEC-*wt*, or remained uninfected. Cells were exposed to Tfn-AF647 concomitant to infection and the subcellular localization of endocytosed Tfn-AF647 was determined by whole cell projection of confocal images. The determination of Tfn-positive perinuclear recycling endosomal (RE) punctum area was determined. Representative confocal images and processed (Otsu-threshold) images whose cell edges have been manually marked, are shown. Results presented in the box plot are mean ± SE of n≥150 infected cells analyzed in three independent experiments. Red arrows point towards the Tfn-positive perinuclear endosomal punctum, while yellow (confocal images) and green (Otsu) arrows point towards infecting microcolonies. Bar = 5 μm. **(B-C) T3SS-dependent increase in Tfn-loading of TfnR and EEA1-positive peripheral endosomes**. HeLa cells were infected with EPEC-*escV*, or EPEC-*wt*, or remained uninfected. Cells were exposed to Tfn-AF647 concomitant to infection, fixed, immunostained with anti-TfnR, or anti-EEA1 antibodies, and imaged by confocal microscopy. The intensity level of Tfn-AF647, TfnR and EEA1 fluorescence was determined with the ‘Image Particle Analysis’ tool. Representative confocal images (except for uninfected cells which looked very similar to the EPEC-*escV* infected cells) and results representing fluorescence intensity of TfnR, Tfn and their ratio **(B)** and EEA1, Tfn and their ratio **(C)** in individual endosomes (n≥600 single particles) are shown in scatter plots. Arrows point towards infecting microcolonies. Bar = 5 μm. **(D) Electron micrograph showing enrichment of endosomes containing Tfn at peripheral EPEC-*wt* infection sites**. HeLa cells were infected with EPEC-*escV* or EPEC-*wt* and concomitantly exposed to Tfn-HRP. Cells were then processed for Tfn-HRP visualization by electron microscopy. Representative images are shown. Blue arrowheads point towards infecting bacteria and red arrows point towards internalized Tfn-labeled endosomes. Bar = 1 μm.

Next, we asked whether the effects observed in polarized cells can be extended to non-polarized cells. Indeed, we found that early and recycling endocytic markers (S4A & S4B Fig), as well as internalized Tfn and s-TfnR (S4C Fig), were recruited at EPEC-*wt*, but not at EPEC-*escV* infection sites. Monitoring the recruitment dynamics of some of these markers using live cell imaging revealed that the process, which is T3SS-dependent, is initiated early upon EPEC microcolony landing on the host (S5 Fig; S1–S6 Movies). Unlike Tfn, fluid-phase uptake (70 kDa Dextran), lysosomal (LysoTracker) and late endosomal (Rab7a) markers were not recruited at EPEC-*wt* infection sites (S6 Fig). Additionally, EPEC also prompts the recruitment of the Rab11a/Myo5b-dependent recycling cargoes, β1-integrins, in non-polarized and polarized cells (S7 Fig [21,33]). Collectively, these data suggest that the recruitment effects were specific for cargoes utilizing CME and Rab11 recycling endosomes. Flow cytometry analysis showed increased levels of Tfn endocytosis and recycling, as well as increased surface bound Tfn in EPEC-*wt*, compared to EPEC-*escV* and uninfected cells (S8A Fig). The EPEC-induced increase in Tfn endocytosis was inhibited by the dynamin inhibitors Dynasore and Dyngo (S8B Fig). These data, together with data presented in Fig 3, suggest that infected plasma membranes of polarized and non-polarized cells promote T3SS-dependent increase in Tfn endocytic turnover.

### EPEC promotes redistribution of endosomes from perinuclear to peripheral sites

In non-polarized cells, internalized TfnR utilizes either a fast constitutive recycling pathway i.e. shuttling to the cell surface from early recycling endosomes or a slow pathway that involves the transport via perinuclear Rab11-positive recycling endosomes [34]. The latter is thought to play a key role in directing recycling proteins to specific and specialized cellular locations on the cell surface, such as the leading edges of motile cells [35,36]. On the basis of these paradigms, we hypothesized that T3SS elements mediate the trafficking of TfnR from the slow perinuclear/pericentriolar recycling endosomal compartment to infection sites confined to the host periphery. To address this hypothesis, endosomes of uninfected, EPEC-*escV*, or EPEC-*wt* infected HeLa cells were loaded with Tfn-AF647 and imaged by confocal microscopy. The area of perinuclear recycling endosomes was determined. As predicted, internalized Tfn accumulated at the perinuclear recycling endosomal compartment of uninfected cells (Fig 3A, **red arrow**). The area occupied by this compartment was not significantly altered in EPEC-*escV* infected cells. In contrast, the area of the perinuclear endosomal compartment was considerably diminished in EPEC-*wt* infected cells (Fig 3A). The majority of Tfn positive endosomes apparently redistributed mainly to the periphery of the cells and concentrated at the infection sites (Fig 3A; **yellow and green arrows**). We applied single particle image analysis to explore whether Tfn is enriched in individual peripheral endosomes of the infected cells. As expected, data showed significant enrichment of Tfn in TfnR (Fig 3B) and in EEA1 (Fig 3C)-positive peripheral endosomes in EPEC-*wt*, but not in EPEC-*escV* or uninfected cells. EEA1 acts downstream of Rab4 (fast recycling) and Rab5 (early) endosomes [37,38,39]. Thus, the fact that Tfn was enriched in EEA1-positive endosomes suggests that EPEC type III secreted elements shifted the pathway of Tfn from slow to fast recycling endosomes. The increased abundance of Tfn-positive endosomes at peripheral infection sites in EPEC-*wt* compared to EPEC-*escV* infected cells could also be observed at the ultrastructural level (Fig 3D).

### EPEC type III secreted components prompt the shuttling of Rab11a/Tfn endosomes from perinuclear to peripheral infection sites

**Fig 4:**
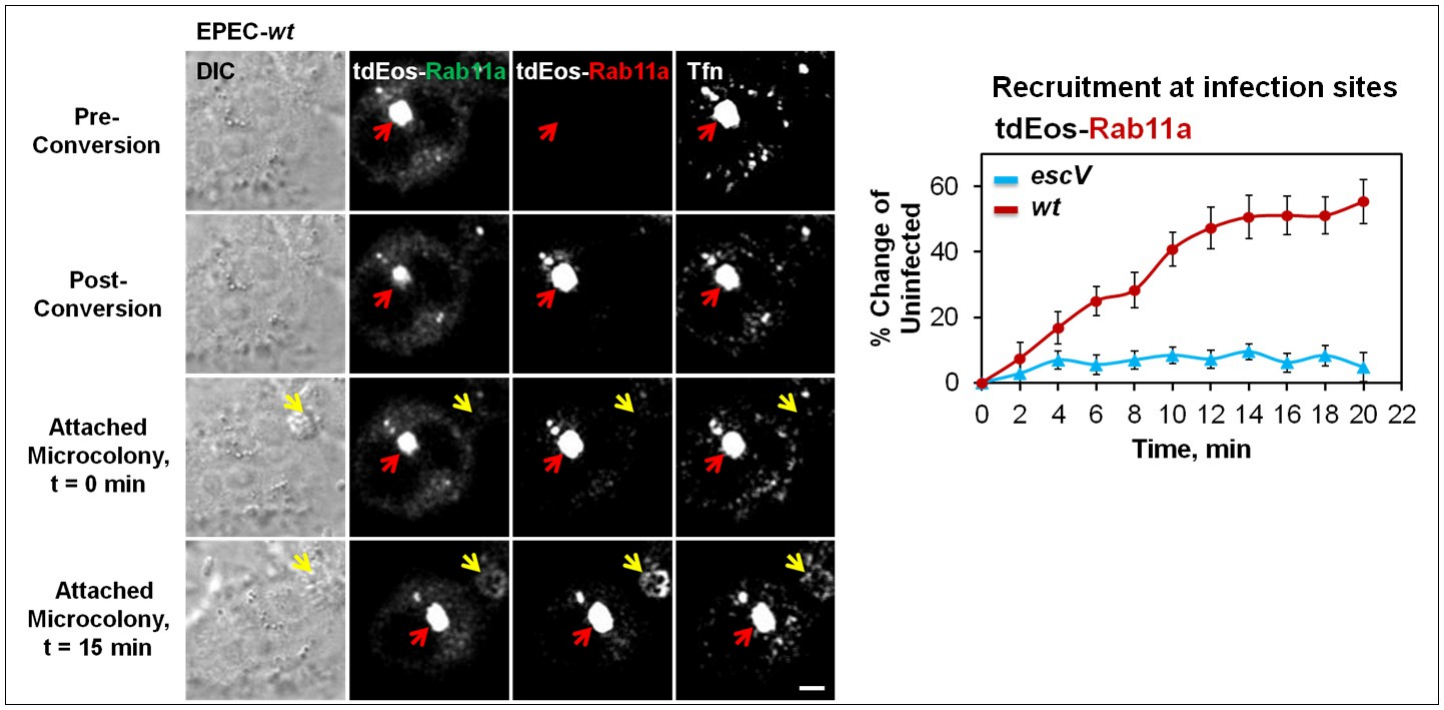
Shuttling of Rab11a and Tfn from perinuclear recycling endosomes to peripheral infection sites. HeLa cells transiently expressing tdEos-Rab11a were exposed to Tfn-AF647 and then infected with EPEC-*escV*, or EPEC-*wt*. Immediately upon bacterial attachment to the host cell, tdEos-Rab11a and Tfn labeled central puncta were subjected to photo-conversion and images were acquired by live-cell confocal microscopy (S7 and S8 Movies). Representative images and quantitative analysis of the photo-converted tdEos-Rab11a recruitment at infection sites are shown. Results are mean ± SE; n≥20 bacterial microcolonies (yellow arrow) were analyzed in three independent experiments. Red arrow points towards a tdEos-Rab11a/Tfn positive perinuclear endosomal punctum subjected to photoconversion. Bar = 5 μm.

We asked whether vectorial shuttling of Tfn/Rab11a-positive endosomes takes place from perinuclear to peripheral infection sites. To this end, HeLa cells were transfected with Rab11a fused to photoconvertible tdEos encoding plasmid (tdEos-Rab11a). These cells were loaded with Tfn-AF647 concomitant to EPEC-*wt* or EPEC-*escV* exposure. The Tfn-AF647 and tdEos-Rab11a labeled perinuclear endosomes (Fig 4; **Pre-Conversion; red arrow**) were selected and irreversibly photoconverted (Fig 4; **Post-Conversion; red arrow**), and time-lapse imaging was applied to track the distribution of the photoconverted Rab and internalized Tfn-AF647 over time (S7 and S8 Movies). A representative image of EPEC-*wt* infected cells (Fig 4; **t = 15; yellow arrow**) and quantitative analysis of the time-dependent accumulation of photoconverted Rab11a at EPEC-*wt* and EPEC-*escV* infection sites are shown (Fig 4). Data clearly show T3SS-dependent recruitment of the photoconverted Rab11a at bacterial infection sites, suggesting that type III secreted components elicit the shuttling of Rab11a/Tfn-positive endosomes from perinuclear recycling endosomes to peripheral infection sites.

To further elucidate this notion, we used Dynasore and Dyngo, which inhibit endocytosis but not recycling of clathrin-dependent cargoes [40]. HeLa cells were first treated with Dynasore or Dyngo and then exposed to EPEC and Tfn-AF647. Cells treated with DMSO served as controls. The localization of Tfn, Rab5a, EEA1 (endocytic markers) and Rab11a (recycling marker) in these cells was analyzed by confocal microscopy. Interestingly, while Tfn, Rab5a and EEA1 showed very low levels of clustering at infection sites, Rab11a displayed significantly higher clustering at these sites (S9A Fig). If Tfn was internalized into the cells prior to exposure to Dyngo along with EPEC-*wt*, both Tfn and Rab11a clustered efficiently at infection sites (S9B Fig). Clustering of F-actin was not affected in any of the experiments. These results suggest again that Tfn/Rab11a recruited at peripheral infection sites are contributed by recycling endosomes.

### Myo5b plays a role in EPEC-induced recruitment of endosomes to infection sites

**Fig 5:**
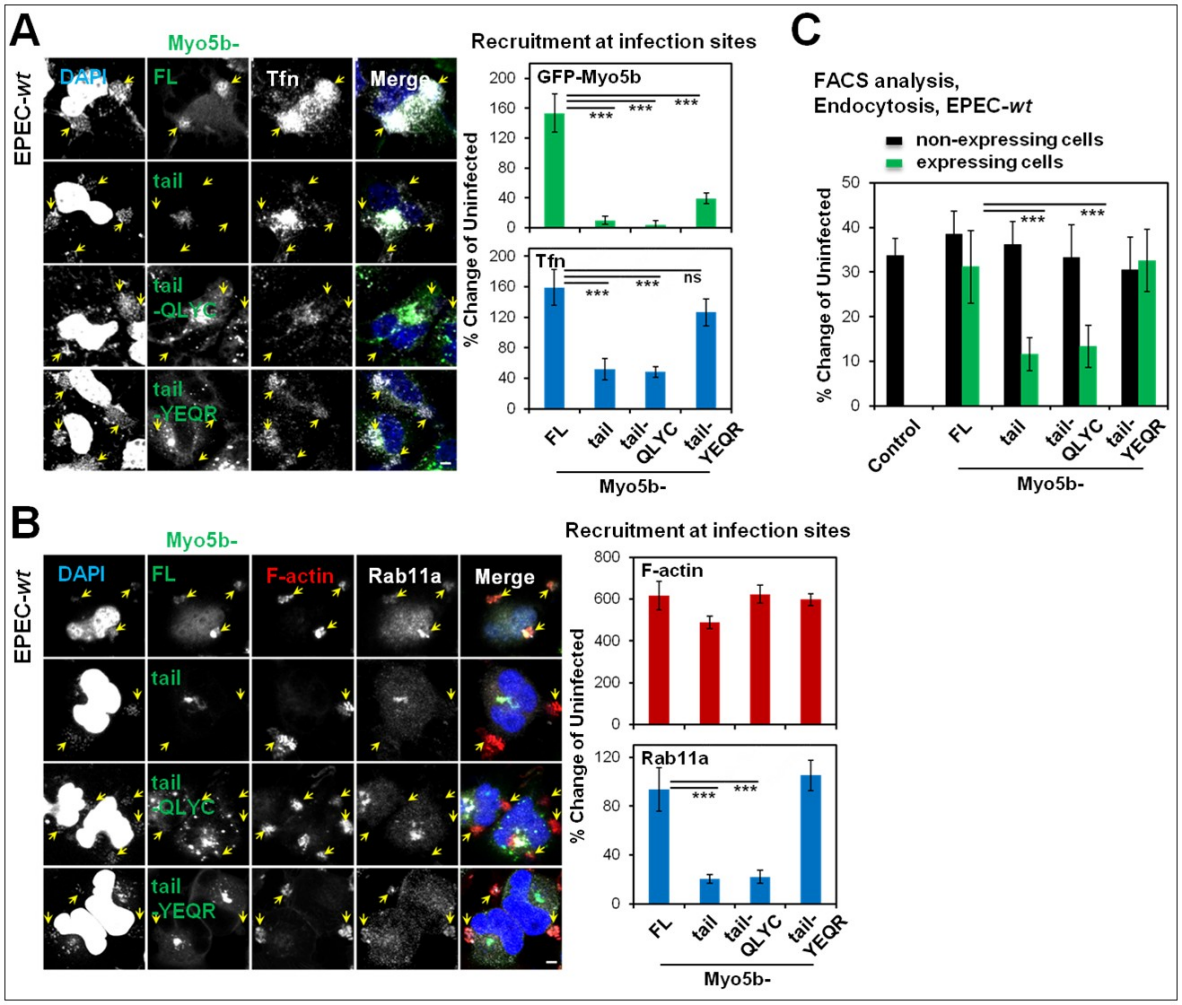
Myo5b is essential for T3SS-dependent recruitment of Rab11a/Tfn-positive endosomes to infection sites and increased endocytic activity. **(A) Effects of GFP-Myo5b expression on Tfn recruitment at infection sites**. HeLa cells transiently expressing the indicated GFP-Myo5b constructs were infected with EPEC-*wt* and exposed to Tfn-AF647. Cells were fixed and stained with DAPI and subjected to confocal imaging. Representative images and quantitative analysis of the recruitment of the indicated markers at infection sites are shown. Results are mean ± SE; n≥30 bacterial microcolonies were analyzed in at least three independent experiments. Bacterial microcolonies are pointed out by arrows. Bar = 5 μm. **(B) Effects of GFP-Myo5b expression on F-actin and Rab11a recruitment at infection sites**. HeLa cells transiently expressing the indicated GFP-Myo5b constructs were subjected to EPEC-*wt* infection, fixed, immunostained with anti-Rab11a antibodies and then stained with TR-phalloidin (F-actin) and DAPI (DNA). Representative images and quantitative analysis of marker recruitment at infection sites are shown. Results are mean ± SE; n≥30 microcolonies were analyzed in at least three independent experiments. Arrows point towards infecting bacterial microcolonies. Bar = 5 μm. **(C) Effects of GFP-Myo5b expression on Tfn endocytosis**. HeLa cells expressing the indicated GFP-Myo5b constructs were infected with EPEC-*wt* concomitant to Tfn-AF647 exposure, or left uninfected. The levels of internalized ligand in GFP-expressing cells, and in cells that showed no detectable GFP expression ('non-expressing cells'), were measured by FACS. Untransfected cells served as control. Results are mean ± SE; n≥12 were analyzed in at least three independent experiments.

As the movement of TfnR-Rab11 positive recycling endosomes is controlled by Myo5b motors [32], we asked whether Myo5b is involved in their recruitment at infection sites. To address this question, the following constructs were ectopically expressed in HeLa cells: GFP-Myo5b-FL, the GFP-Myo5b-tail mutant (Myo5b-tail), which harbors the Rab8/Rab11 C-terminal binding motif but lacks the ability to mobilize Rab11-dependent cargo due to deleted N-terminal motor domain, the GFP-Myo5b tail-QLYC mutant, which binds Rab11 but not Rab8a, and the GFP-Myo5b-tail-YEQR mutant, which does not bind Rab11 but binds Rab8a [41] (see S3 Table). Consistent with previous data, over expression of Myo5b-tail and Myo5b-tail-QLYC, but not of Myo5b-FL or Myo5b-tail-YEQR, resulted in the sequestration of Rab11a and Tfn into large intracellular puncta (S10A Fig). These data further corroborate the existence of a correlation between the ability of these Myo5b mutants to sequester Rab11a-Tfn endosomes and to reduce s-TfnR levels (S10B Fig). These effects are contributed by the ability of Myo5b-tail mutants to inhibit Tfn recycling, resulting in the retention and accumulation of internalized Tfn within the cells (S10C Fig). Infection of these cells with EPEC-*wt* resulted in significant Tfn (Fig 5A) and Rab11a (Fig 5B) recruitment in Myo5b-FL or Myo5b-tail-YEQR, but not in Myo5b-tail or Myo5b-tail-QLYC expressing cells. Infection with EPEC-*wt* induced Tfn endocytosis in Myo5b-FL or Myo5b-tail-YEQR, but not in Myo5b-tail or Myo5b-tail-QLYC expressing cells (Fig 5C). These data suggest that EPEC requires accessible Rab11 and functional Myo5b motors for recruiting TfnR and Rab11a-positive endosomes to peripheral plasma membrane infection sites, and for stimulating Tfn endocytosis.

### Rab11 is essential for Tfn recruitment at infection sites

**Fig 6:**
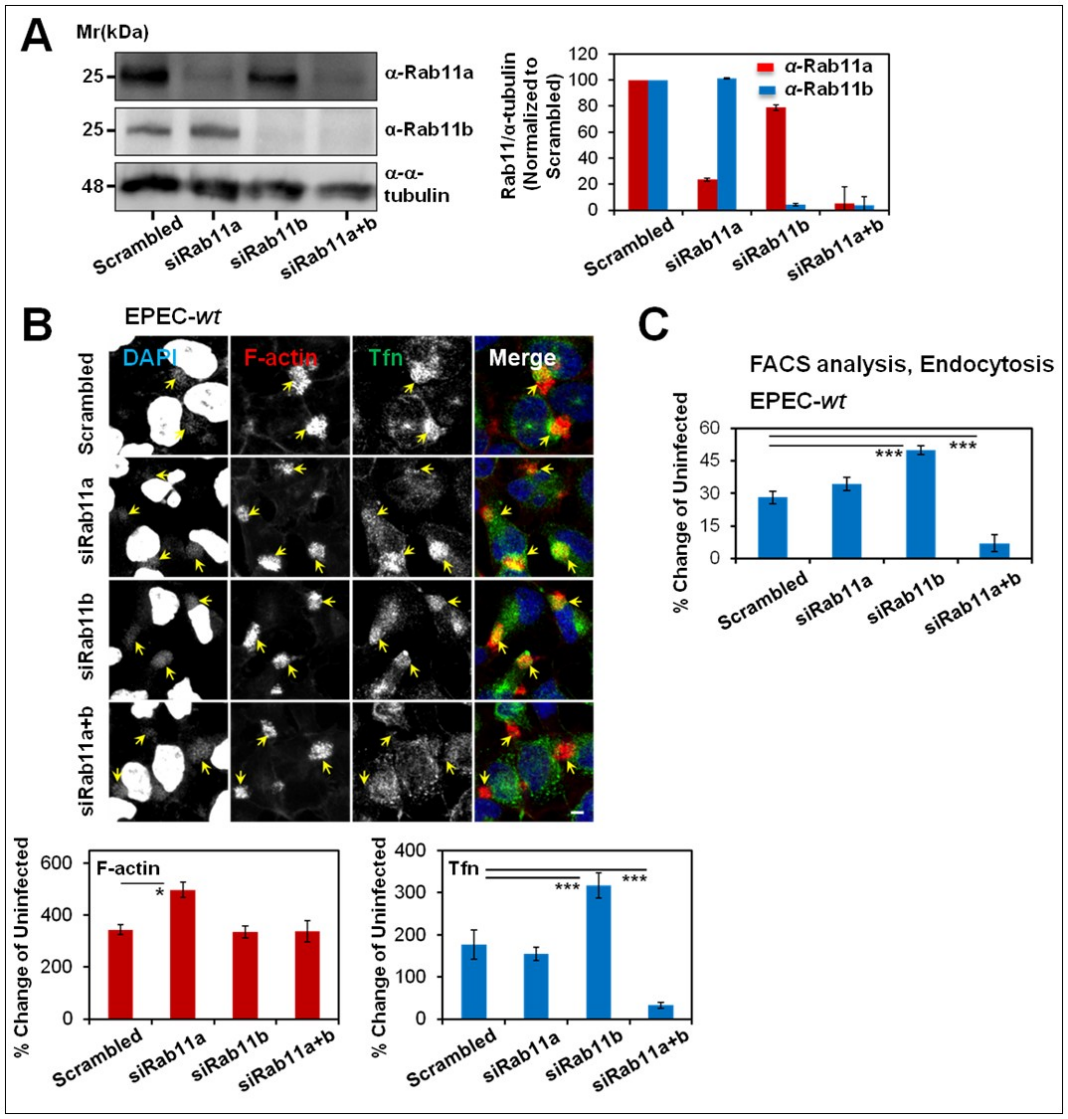
Rab11a and Rab11b are essential for Tfn-positive endosome recruitment to infection sites and increased endocytic activity. **(A) Rab11a and Rab11b silencing by siRNA**. HeLa cells were treated with scrambled siRNA, siRab11a, siRab11b or a mixture of siRab11a and siRab11b (a+b). Cells were subjected to Western blotting analysis using anti-Rab11a, anti-Rab11b, and anti-α-tubulin antibodies (S4 Table). A representative Western blot and quantitative analysis of the Rab11 levels are shown. Results are mean ± SE of three experiments. **(B) Expression of Rab11a and Rab11b is required for Tfn recruitment at EPEC-*wt* infection sites**. HeLa cells transfected with the indicated siRNAs were infected with EPEC-*wt* and exposed to Tfn-AF488. Cells were stained with TR-phalloidin and DAPI, and imaged by confocal microscopy. Representative images and quantitative analysis of markers’ recruitment at infection sites are shown. Results are mean ± SE; n≥30 infecting microcolonies were analyzed in three independent experiments. Arrows point towards infecting microcolonies. Bar = 5 μm. **(C) Rab11a and Rab11b are required for EPEC- mediated increase in Tfn endocytosis**. HeLa cells treated with the indicated siRNAs were infected with EPEC-*wt* and exposed to Tfn-AF488. The levels of internalized ligand were measured by flow cytometry. Results are mean ± SE; n≥12 were analyzed in three independent experiments.

The involvement of Rab11 in endosomal subversion by EPEC was further examined using selective gene silencing by siRNA. The expression level of Rab11a and Rab11b was either individually or simultaneously knocked-down using siRNA, as demonstrated in Fig 6A. Consistent with previous studies [42], internalized Tfn localized mainly to the cell periphery, rather than to the perinuclear recycling endosome, in the siRab11a+b depleted cells (S11A Fig). Additionally, a significant diminishment in perinuclear recycling endosomal area size (S11B Fig), and an increase in Tfn endocytic turnover (S11C Fig) were observed in these cells. Interestingly, Tfn clustering at EPEC-*wt* infection sites was apparent in cells treated with siRab11a, or siRab11b, but not in cells treated with siRab11a+b (Fig 6B), suggesting that the two Rab11 variants share structural information that supports the EPEC-mediated recruitment of Tfn to infection sites. Our flow cytometry data showed that EPEC-*wt* failed to increase Tfn endocytosis in the Rab11a+b depleted cells (Fig 6C). The inability of EPEC to further enhance endocytosis in these cells could be attributed to lack of capacity of the pathogen to further redistribute the already peripherally distributed recycling endosomes, and to concentrate them at infection sites.

### EspF and Map stimulate the recruitment of Tfn/TfnR, Myo5b and Rab11a at infection sites in nonpolarized cells

**Fig 7:**
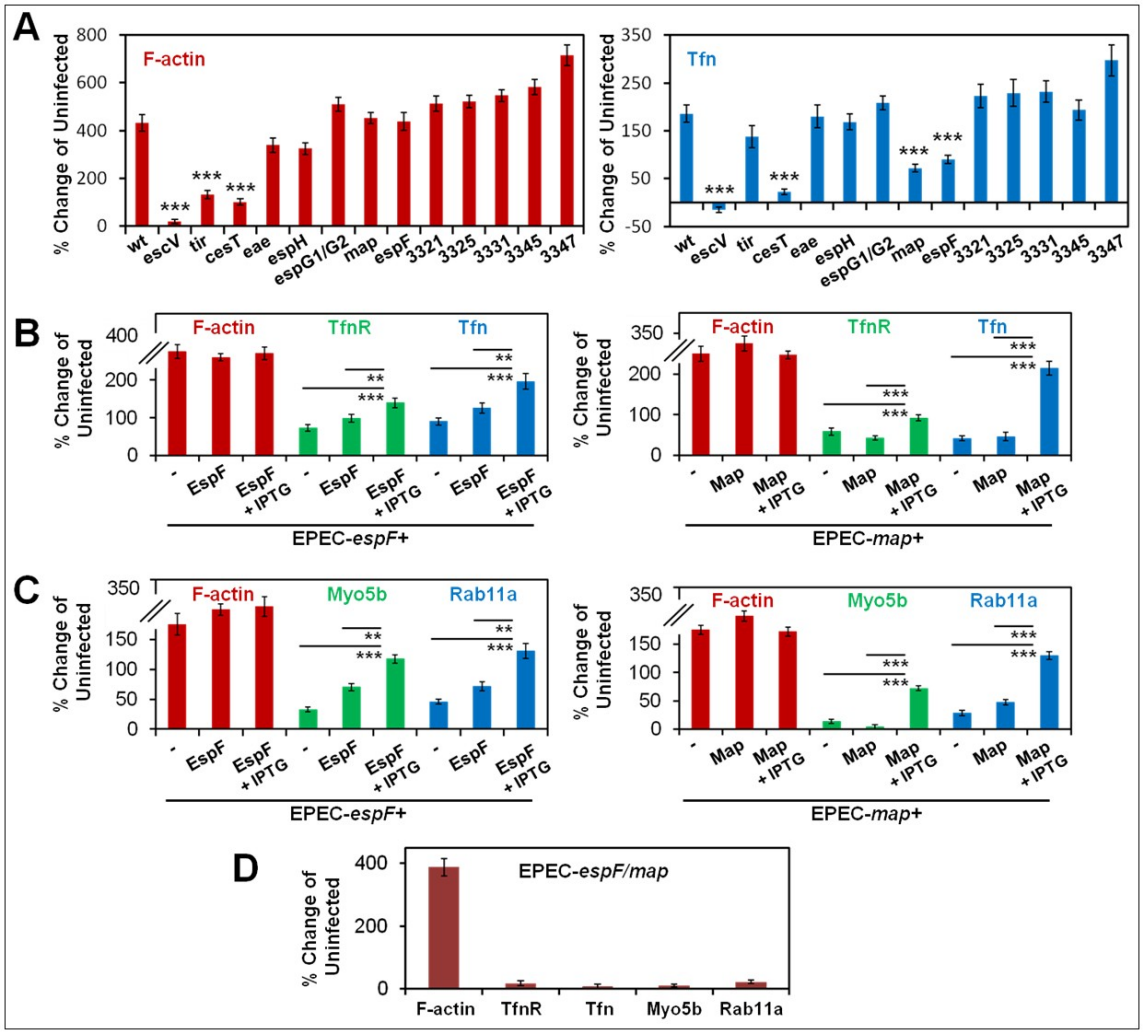
EspF and Map mediate the recruitment of Tfn/TfnR and Myo5b/Rab11a at infection sites. **(A) Screening for type III secreted protein effectors mediating Tfn recruitment to infection sites**. HeLa cells were infected with EPEC-*wt*, or with the indicated mutant strains (S1 Table), or remained uninfected. Cells were exposed to Tfn-AF647 during the infection, fixed, stained with TR-phalloidin (F-actin) and DAPI and analyzed by confocal microscopy. Quantitative analysis of F-actin and Tfn recruitment at infection sites are shown. Results are mean ± SE; n≥30 bacterial microcolonies. **(B) EspF and Map are essential for Tfn/TfnR recruitment at infection sites**. HeLa cells were infected with the EPEC-*espF* or *map* mutant strains and their corresponding *espF*+EspF, or *ma*p+Map complemented strains. Effector protein expression was induced by IPTG. Cells were exposed to Tfn-AF647 during infection, fixed, immunolabeled with anti-TfnR antibodies, stained with TR-phalloidin and DAPI and analyzed by confocal microscopy. Quantitative analysis of F-actin, TfnR and Tfn recruitment at infection sites are shown. Results are mean ± SE of n≥30 bacterial microcolonies analyzed in three independent experiments. Arrows point towards infecting microcolonies. Bar = 5 μm. **(C) EspF and Map are essential for Myo5b and Rab11a recruitment at infection sites**. HeLa cells were infected with the indicated EPEC strains, immunolabeled with anti-Myo5b and anti-Rab11a antibodies, stained with TR-phalloidin and DAPI and visualized by confocal microscopy. Quantitative analysis of F-actin, Myo5b and Rab11a recruitment at infection sites are shown. **(D) Simultaneous mutation of *espF* and *map* markedly reduces the recruitment of Tfn, TfnR, Myo5b and Rab11a at infection sites**. HeLa cells were infected with the EPEC-espF/map mutant strain and the level of the recruitment of the indicated protein markers at infection sites was evaluated as in panels B&C. Results are mean ± SE; n≥30 infecting microcolonies analyzed in three independent experiments. Representative confocal images are shown in S12 Fig.

Next, we aimed at identifying type III secreted effectors that might mediate the clustering of Rab11a and Tfn-positive endosomes at bacterial infection sites. HeLa cells were infected with a series of EPEC strains mutated in their LEE, or non-LEE effector encoding genes, and concomitantly exposed to Tfn-AF647. Cells were then fixed, subjected to F-actin staining and confocal imaging. Results show that, compared to EPEC-*wt* infected cells, infection with EPEC-*espF*, EPEC-*map*, EPEC-*cesT*, or EPEC-*escV* resulted in low levels of Tfn/TfnR clustering at infection sites (Fig 7A). F-actin recruitment to EPEC-*espF* and EPEC-*map* infection sites was not significantly altered compared to EPEC-*wt*, suggesting that the reduced Tfn clustering is not contributed by the capacity of EPEC to recruit F-actin. Inefficient Tfn/TfnR clustering in the EPEC-*cesT* infected cells is likely contributed by inefficient Map translocation that is partially mediated by the CesT chaperone [9,43]. The involvement of CesT-dependent effectors other than Map in the clustering effect cannot be excluded at this point. Tfn clustering at infection sites was restored upon cell infection with EPEC-*espF*+EspF or EPEC-*map*+Map (Fig 7B, S12A Fig). Infection with these strains also prompted the recruitment of Rab11a and Myo5b at infection sites (Fig 7C, S12B Fig), where EspF and Map staining partially overlapped with Rab11a and Myo5b (S12C Fig), suggesting that the effector proteins reside in close proximity to the host proteins. Simultaneous deletion of *espF* and *map* from the bacterial genome resulted in a complete loss of EPEC-stimulated recruitment of all indicated endocytic markers to the infection sites (Fig 7D, S12D Fig). Notably, both complemented EPEC strains were capable of translocating the expressed effectors into the infected cells (S13A Fig) and a fraction of translocated EspF and Map has been located in mitochondria-free regions at these sites (S13B Fig), suggesting that the translocated protein effectors may exert their activities prior to their targeting to mitochondria.

### EspF and Map increase the Tfn endocytic turnover in nonpolarized cells

**Fig 8:**
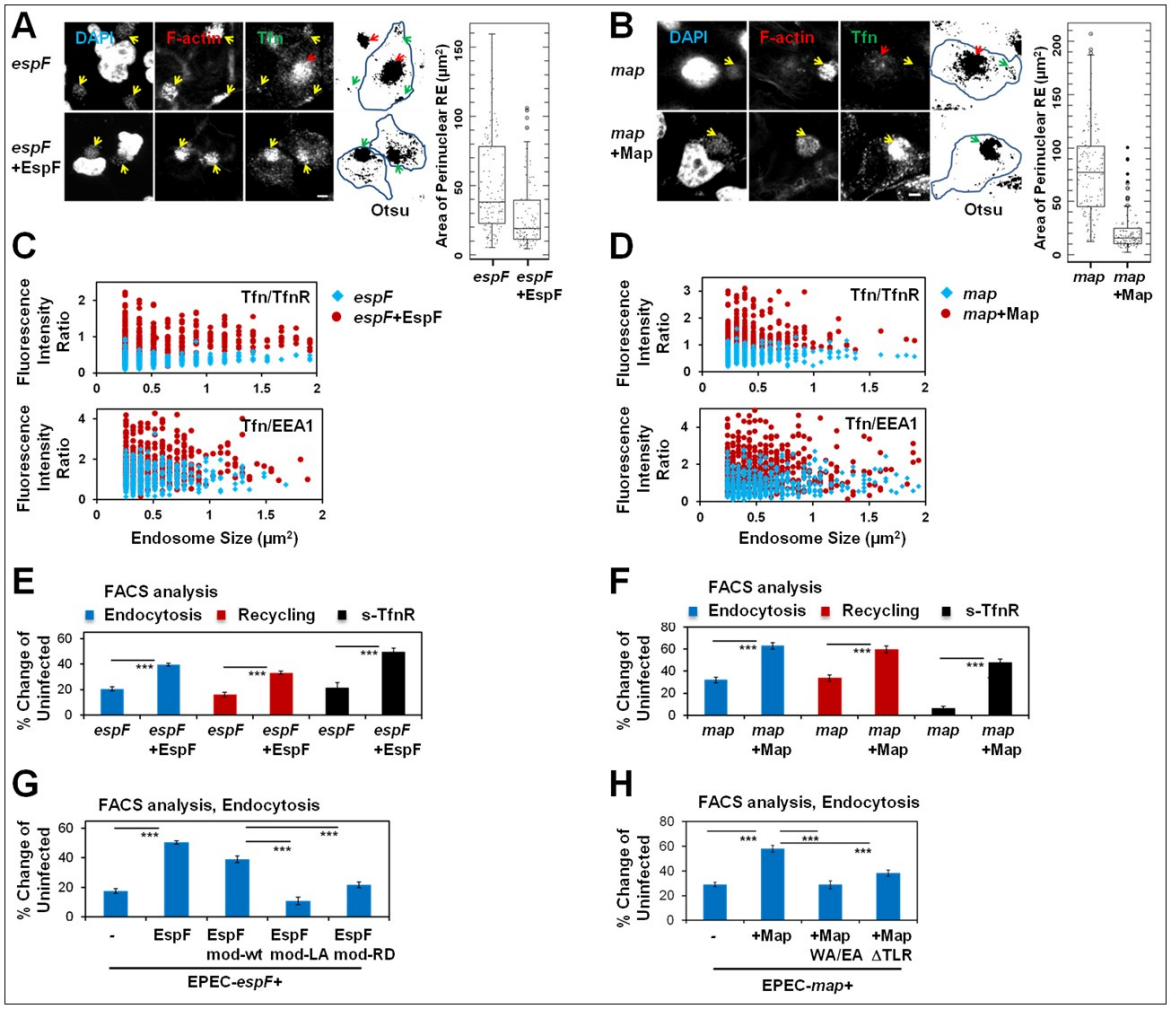
EspF and Map mediate alterations in endosomal distribution and stimulate an increase of endocytic turnover. **(A-B) Translocated EspF and Map prompt a reduction in Tfn-positive perinuclear endosome area and an increase in endosomal abundance at peripheral infection sites**. HeLa cells were infected with the indicated EPEC strains and exposed to Tfn-AF647 during infection. The area of Tfn-positive perinuclear recycling endosome (RE) punctum was determined. Results are mean ± SE; n≥75 infected cells were analyzed in three independent experiments. Red and yellow arrows point towards the Tfn positive perinuclear endosomal puncta and infecting bacterial microcolonies, respectively. Bar = 5 μm. **(C-D) EspF and Map-dependent increase in Tfn-loading of TfnR and EEA1-positive peripheral endosomes**. HeLa cells were infected with the indicated EPEC strains and exposed to Tfn-AF647 during the infection. Quantitative analyses of the fluorescence intensities of Tfn, TfnR and EEA1 in individual endosomes were determined. Results present fluorescence intensity of Tfn normalized to the fluorescence intensity of TfnR or to the fluorescence intensity of EEA1, from n≥500 single particles (endosomes). **(E-F) EspF and Map increased the Tfn endocytic turnover**. HeLa cells were infected with the indicated EPEC strains and exposed to Tfn-AF647 during infection. Tfn-AF647 endocytosis, recycling and surface-bound ligand were determined by flow cytometry. Results are mean ± SE of n≥6 samples from three independent experiments. **(G-H) The interactions of EspF and Map with host proteins are essential for eliciting Tfn endocytosis**. HeLa cells were infected with the indicated EPEC strains (see S3 Table) and exposed to Tfn-AF647 during the infection. The capacity of cells to endocytose Tfn was determined by flow cytometry. Results are mean ± SE; n≥6, analyzed in three independent experiments.

Infection with EPEC-*espF*+EspF and EPEC-*map*+Map evoked a sharp decrease in perinuclear Tfn-positive endosomal area (Fig 8A and 8B), a concomitant increase in Tfn/TfnR and Tfn/EEA1 intensity in the peripheral endosomes (Fig 8C and 8D), and an increase in Tfn endocytic turnover (Fig 8E and 8F). Together, these results suggest that upon translocation, EspF and Map promote the redistribution and clustering of Rab11a/Tfn positive endosomes at peripheral infection sites to increase the endocytic turnover at the infected plasma membrane. EspF contains three proline-rich modules that can bind the N-WASP/WASL and sorting nexin9 (SNX9) [22,23] proteins. We examined whether EPEC-*espF* complemented with plasmids encoding EspF mutants deficient in the interactions with N-WASP, or SNX9 (EspF mod-LA and EspF mod-RD, respectively; S3 Table) affects the ability of EspF to alter Tfn endocytosis. Results showed that infection with either bacterial strain resulted in diminished Tfn endocytosis compared to EPEC-*espF* + EspF or EPEC-*espF*+EspF mod-*wt*, and that is similar to the level obtained by EPEC-*espF* (Fig 8G). Map activates Cdc42 by a WXXXE guanine nucleotide exchange (GEF) motif [44,45,46]. Map has also been reported to possess a C-terminal TRL PDZ class I binding motif [47]. EPEC-*map* strains complemented with Map encoding plasmids mutated in either one of the aforementioned two motifs (S3 Table) failed to elicit increased endocytic activity (Fig 8H). Together, these data suggest a role for EspF and Map binding host proteins in eliciting Tfn endocytic turnover in response to EPEC infection.

### EspF alone stimulates Tfn endocytic turnover and transcytosis in polarized MDCK cells

**Fig 9:**
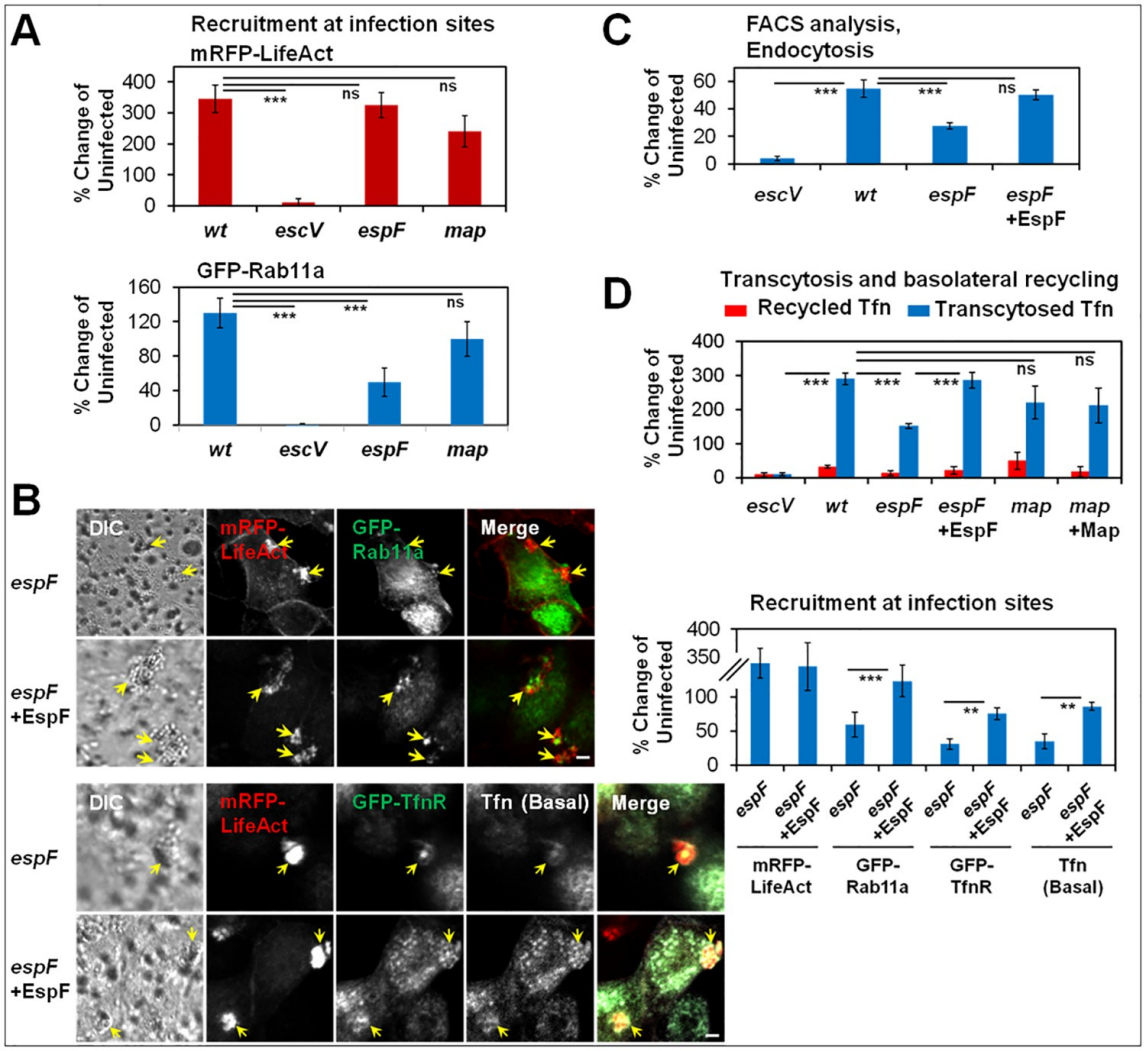
EspF, but not Map, mediates the recruitment of TfnR and Rab11a at apical infection sites in polarized epithelial cells. **(A) Infection with an EPEC-*espF*, but not EPEC-*map*, mutant results in diminished recruitment of Rab11a at infection sites**. Polarized MDCK cells transiently co-expressing mRFP-LifeAct and GFP-Rab11a were infected with the indicated EPEC strains (S1 Table) and imaged by live-cell confocal microscopy. Results are mean ± SE of n≥20 bacterial microcolonies analyzed in three independent experiments. (**B) EspF-dependent recruitment of Rab11a, TfnR and Tfn at infection sites**. Polarized MDCK cells transiently co-expressing mRFP-LifeAct and GFP-Rab11a, or mRFP-LifeAct and GFP-TfnR, were infected with EPEC-*espF* or EPEC-*espF*+EspF. The latter cells were exposed to basolateral Tfn-DL649 during the infection. Cells were then imaged by live-cell confocal microscopy, and the degree of recruitment of the expressed host proteins and internalized Tfn at apical infection sites were quantitatively determined. Arrows point to infection sites. Results are mean ± SE; n≥20 bacterial microcolonies (arrows) were analyzed in three independent experiments. **(C) EspF-dependent increase in endocytic turnover**. The apical surface of polarized MDCK-GFP-TfnR cells was infected with the indicated EPEC strains or remained uninfected. Cells were exposed to apical Tfn-DL649 and cell-associated Tfn was determined by FACS analysis. Results are mean ± SE of six independent experiments. **(D) EspF-dependent increase in Tfn transcytosis**. Polarized MDCK-PTR9 cells were infected with the indicated EPEC strains, or remained uninfected. Tfn transcytosis was analyzed as in Fig 1C. Results are mean ± SE of five independent experiments.

Next, we asked whether EspF or Map can affect endosomal recruitment and traffic in polarized epithelial cells. Polarized MDCK cells co-expressing mRFP-LifeAct and GFP-Rab11a were infected with EPEC-*wt*, EPEC-*escV*, EPEC-*espF*, EPEC-*map*, or left uninfected. Cells were fixed, and the recruitment of the expressed proteins at infection sites was analyzed by confocal microscopy (Fig 9A). Data showed that compared to EPEC-*wt*, the recruitment of GFP-Rab11a to infection sites of EPEC-*escV* and EPEC-*espF*, but not of EPEC-*map*, was significantly reduced. Neither EPEC-*espF* nor EPEC-*map* had an effect on mRFP-LifeAct recruitment to these sites, suggesting again that the effects obtained in EPEC-*espF* infected cells were not contributed by alterations in F-actin. We then infected polarized MDCK cells co-expressing mRFP-LifeAct and GFP-Rab11a, or mRFP-LifeAct and GFP-TfnR with EPEC-*espF*, or EPEC-*espF*+EspF. The results showed diminished GFP-Rab11a and GFP-TfnR clustering in EPEC-*espF* compared to EPEC-*espF*+EspF infected cells, suggesting that the recruitment of these proteins to apical infection sites is EspF dependent (Fig 9B, **upper**). The clustering of Tfn internalized from the basolateral pole of the GFP-TfnR expressing cells at apical infection sites also showed EspF dependence (Fig 9B, **lower**). Based on previous observations, these data suggest that EspF increases Tfn-endocytic turnover and Tfn basolateral-to-apical transcytosis. Indeed, data obtained by flow cytometry (Fig 9C) and transcytosis assays (Fig 9D) concur with this notion.

### EspF interacts with the novel SNX18, SNX33 and WIPF1 proteins

**Fig 10:**
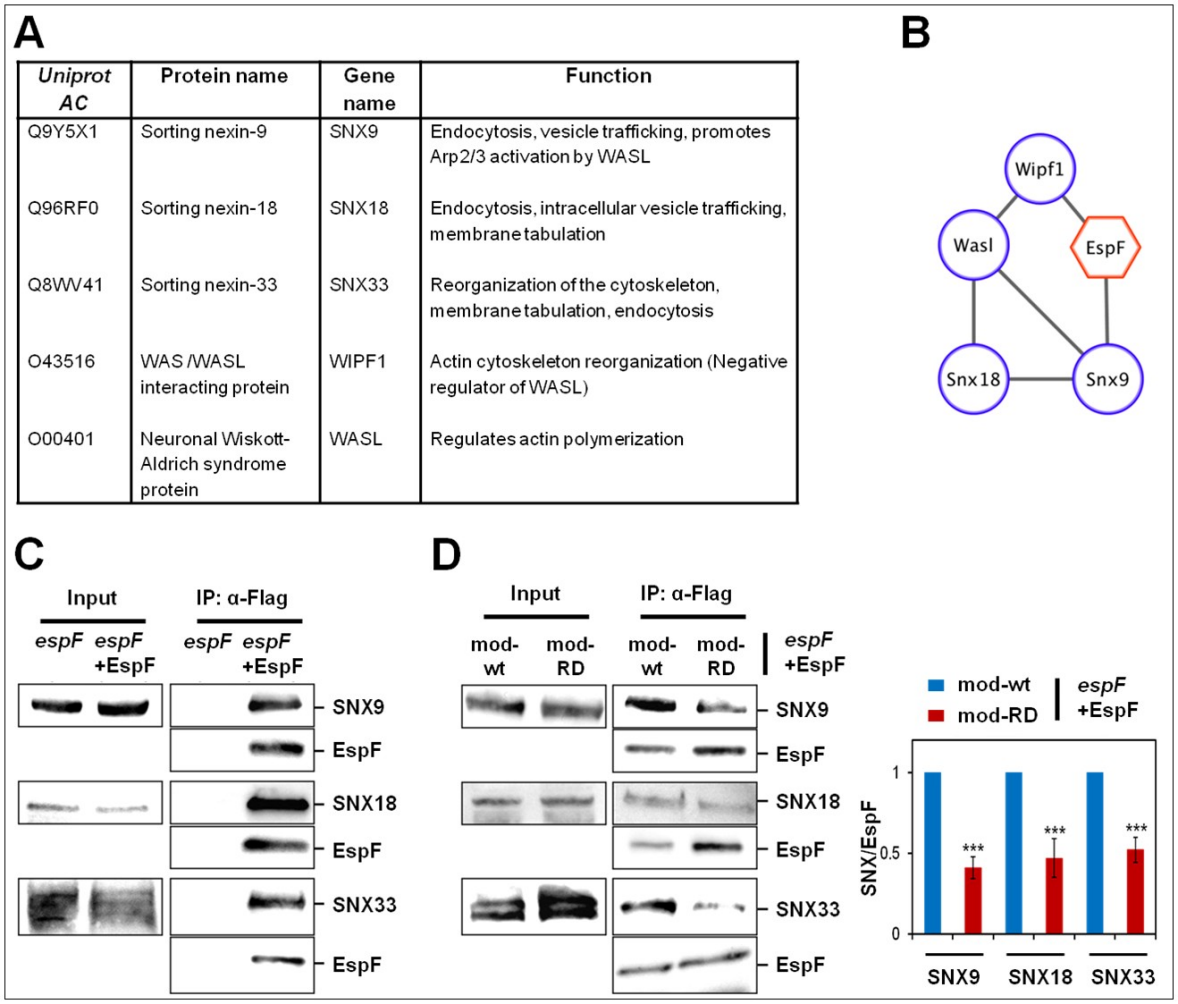
Translocated EspF interacts with SNX9, N-WASP, and the novel SNX18, SNX33, WIPF1 proteins. **(A) Major host cell proteins co-immunoprecipitated with EspF**. HeLa cells were infected with EPEC-*espF* or *espF*+EspF. The Flag-tagged EspF was immunoprecipitated and co-immunoprecipitated proteins were identified, as described in Methods. 232 proteins were identified in at least two of four replicates (S6 Table). Student’s t-test was used to assess which proteins were significantly more abundant in the EspF pull-down compared to the control sample. Six proteins enriched by at least 80-fold with p-value < 0.05 were retrieved. One is EspF itself (not shown), three are sorting nexin proteins (SNX9, SNX18, and SNX33) and two are actin polymerization regulators, WIPF1 (WASP/WASL-interacting protein) and N-WASP (also named WASL). **(B) STRING protein interaction analysis**. To identify potential interactions within the EspF binders, we used the STRING protein interaction database [85], updated in September 2018, under the highest confidence and experimentally validated interaction setting. Cluster analysis shows that WIPF1, WASL, SNX9 and SNX18 form a highly connected interaction network with experimentally validated interactions (based on the STRING annotation), suggesting that these proteins are pulled-down as one interaction unit. Previous studies have identified only SNX9 and N-WASP/WASL as EspF binding partners [22,23]. The EspF interactions were added using the Cytoscape program. We, as a result, identified here three additional interactors, SNX33, SNX18 and WIPF1. **(C) SNX9, SNX18 and SNX33 co-immunoprecipitation with EspF confirmed by Western blotting**. HeLa cells infected with EPEC-*espF* or EPEC-*espF*+EspF were subjected to EspF immunoprecipitation (IP), using anti-FLAG antibodies. The immunoprecipitated EspF and associated proteins were subjected to SDS-PAGE followed by Western blotting analyses. EspF (~26 and 15 kDa) was detected by anti-FLAG antibodies, and co-immunoprecipitated SNX9, SNX18 and SNX33 (~75 kDa) were detected by the appropriate antibodies (S4 Table). The results show that the SNX proteins are specifically co-immunoprecipitated with translocated EspF **(D) Co-immunoprecipitation efficiency of SNX9, SNX18 and SNX33 with EspF**. Experiments were performed as in panel C, except that cells were infected with the indicated EspF mod strains. Results are mean ± SE of three independent experiments. The results show reduced levels of co-immunoprecipitated SNX with EspF mod-RD compared to EspF mod-wt.

Because of the significance of EspF, its interactions with host cell proteins upon translocation were examined. Using co-immunoprecipitation followed by mass-spectrometry analysis we show that aside of the previously reported N-WASP and SNX9, the proteins SNX18, SNX33 and WIPF1 were specifically co-immunoprecipitated with translocated EspF (Fig 10A and S6 Table). STRING analysis identified the possible protein-protein interaction network among these proteins (Fig 10B). The interactions between EspF and SNX, which were more efficient than the interactions with WIPF1 and N-WASP (see “Fold change” S6 Table), could also be confirmed by Western blotting (Fig 10C). Our data also showed a partial, but consistent reduction in the co-immunoprecipitation levels of the three SNX proteins with the EspF mod-RD mutant (Fig 10D), suggesting that these proteins interact with the SNX9 binding motif of EspF.

## Discussion

Our results show that EPEC type III secreted components stimulate the recruitment of clathrin-dependent early (Rab5a, EEA1) and recycling (e.g. Rab11a, Rab11b, Rab4a, Rab25, Myo5b, FIP2) endocytic machineries to plasma membrane infection sites of polarized and non-polarized epithelial cells (S1, S4A and S4B Figs). These rearrangements, which occur early upon EPEC microcolony contacting the host (S5 Fig), coincided with enhanced endocytosis and recycling activities, i.e. increased endocytic turnover, at that host cell surface (Fig 2 and S8 Fig), and with basolateral-to-apical transcytosis of basolaterally recycled cargoes, e.g. TfnR (Fig 1C). The consequence of these events is the enrichment of the infected plasma membrane with specific membrane proteins.

The recruitment of endosomes to infection sites is mediated by several mechanisms. From the ‘host cell perspective’, Myo5b motors are required to mobilize Rab11-positive recycling endosomes and their cargoes to host infection sites. In non-polarized cells, this process involves Myo5b-dependent shuttling of Rab11 endosomes and their cargoes from perinuclear slow recycling endosomes to peripheral infection sites (Figs 3 and 4). In polarized cells, the process involves Myo5b-dependent shuttling of basolaterally internalized cargoes to apical infection sites (Fig 1B). In both cases, the enrichment of peripheral infection sites with recycling endosomes could play an important role in facilitating endocytic turnover near these sites. This hypothesis is supported by previous studies suggesting that the shift in cargo localization from slow perinuclear to fast peripheral recycling endosomes results in an increased endocytic turnover [48,49,50]. In our studies, depletion of Rab11 in non-polarized cells caused loss of perinuclear endosomes, and increased the abundance of peripheral endosomes and endocytic turnover (S11 Fig). Infection with EPEC-*wt*, did not affect the endocytic turnover in these cells (Fig 6), probably because it could not further redistribute the already peripherally redistributed endosomes.

From the ‘pathogen’s perspective', we identified EspF and Map as the protein effectors which mediate endosomal recruitment to infection sites and the increase in endocytic turnover in non-polarized cells (Figs 7 and 8). Interestingly, EspF, but not Map, has been identified as mediating these activities in polarized cells (Fig 9), emphasizing the role of EspF in the process. This conclusion may coincide with recent data suggesting that EspF, but not Map, is involved in the disruption of epithelial cell polarity [19]. EspF and Map may perform these activities immediately upon translocation into the host and prior to reaching mitochondria (S13B Fig). Studies have shown that EspF interacts with high specificity and strong affinity with the eukaryotic SNX9 and N-WASP to mediate endocytic membrane remodeling coupled to Arp2/3 actin nucleation [23]. Using co-immunoprecipitation followed by proteomics and Western blotting analyses, we confirmed these interactions (Fig 10A and 10C and S6 Table). SNX9, SNX18 and SNX33, which represent a subgroup of SNX-BAR (Bin, Amphiphysin, Rvs) sorting nexins are predicted to function as modulators of endocytosis and endosomal sorting [51]. Studies have shown that SNX18 interacts with the Rab11 family interacting protein (FIP) 5, and that these interactions are required for membrane tubulation and polarized transport of apical proteins [52]. We identified SNX18, SNX33 and WIPF1 as novel interactors of EspF (Fig 10A and 10C and S6 Table). Thus, while the interactions of EspF with SNX9 and with WASP/WIPF1 may be linked to the ability of the effector protein to activate endocytosis (Figs 8G and 9C), the interactions with SNX18 could play a role in Rab11/FIP5 dependent cargo shuttling to the infected apical plasma membrane. Interestingly, EspF seems to interact with SNX18 and SNX33 through the previously identified SNX9 binding motif (Fig 10D). These SNX-EspF interactions may mediate the EspF-dependent increase in endocytic activity.

Our data also indicate that the Map’s GEF and PDZ binding domain play a role in eliciting endocytic turnover (Fig 8H). These observations are consistent with previous studies implicating Cdc42 in modulating a functional connection between the actin cytoskeleton and endocytic traffic [53,54], and in the induction of endocytic membrane turnover [55]. The significance of the PDZ binding motif of Map, which binds EBP50, could be explained by a role attributed to EBP50 in endocytic recycling [56]. Notably, Clements et al have reported that translocated EspG imposed inhibition of endosomal recycling [21] by targeting the TfnR/Rab11/Rab35 positive recycling endosomes [20]. Thus, it is possible that EspG counterbalances the endocytic and recycling activities elicited by EspF and Map.

The functional significance of these events with respect to the pathogen and host cell physiology could be broad. It would be reasonable to assume that the lumen of the infected gut, particularly following experiencing acute diarrhea, is depleted of nutrients. We suggest that upon landing on the apical cell surface of small intestinal enterocytes, the pathogen generates a local niche that enables nutrient acquisition from the serosal environment. One way of achieving this is by apical missorting of basolateral plasma membrane proteins which carry micronutrients from the blood, such as TfnR bound to iron-loaded Tfn. Indeed, bacterial pathogens have evolved remarkably efficient strategies to hijack iron from their host, a critical micronutrient for their homeostasis and growth, [57], including from transferrin [58,59]. Here we show increased EPEC colonization of host cells exposed to iron-loaded Tfn that has been introduced either to the basolateral or the apical poles of polarized MDCK-PTR9 cells. A similar phenomenon was seen when these cells were exposed to free iron. However, such an increase was not observed in parental MDCK cells, which express very low levels of the native canine receptors (S14 Fig). These data suggest that apically infecting EPEC is capable of hijacking and accessing iron bound Tfn even when the ligand is internalized from the basolateral side of the cells, likely by importing the cargo to the apically infected plasma membrane via transcytosis (Fig 1). This process seems to be vital for promoting bacterial colonization of the infected cell surface. Another possible way is the weakening of the tight junction barrier functions, which may allow the infiltration of nutrients from the blood to bacteria adhered to apical cell-cell junctions [5]. Interestingly, tight junctions disruption has been attributed to several EPEC effectors, including effectors investigated in this study, EspF and Map [10]. We suggest that through both these activities the pathogen gains a survival advantage over other bacteria (e.g. commensals), resulting in successful colonization and infection of the gut.

Previous studies have shown that infection with the murine A/E pathogen *C*. *rodentium* resulted in mislocalization of the water channels aquaporin 2 and 3 (AQP2 and AQP3) from the host cell membranes to the cytoplasm. The process, which was partially dependent on EspF and EspG, correlated with a diarrhea-like phenotype [60]. More recently, transiently expressed eGFP-AQP3 was observed at EPEC infection sites [61]. Studies have shown that similar to the TfnR, AQP2 utilizes the clathrin-dynamin dependent endocytic pathway [62,63], as well as the Myo5b and Rab11-FIP2 recycling route [64]. Moreover, AQP2 has been localized to the apical or basolateral surfaces of polarized epithelial cells [63,65], while AQP3 was localized exclusively to the basolateral surface of these cells [66,67]. Here we found that native AQP2 and AQP3 are recruited to EPEC infection sites in a T3SS-dependent manner and that both water transporters colocalized with Myo5b (S15 Fig). Thus, it is possible that similarly to TfnR, EPEC utilizes type III secreted effectors to mislocalize aquaporins to Myo5b/Rab11 recycling endosomes, a process that could alter the water homeostasis in the infected cells and thereby lead to the diarrheal effect.

**Fig 11:**
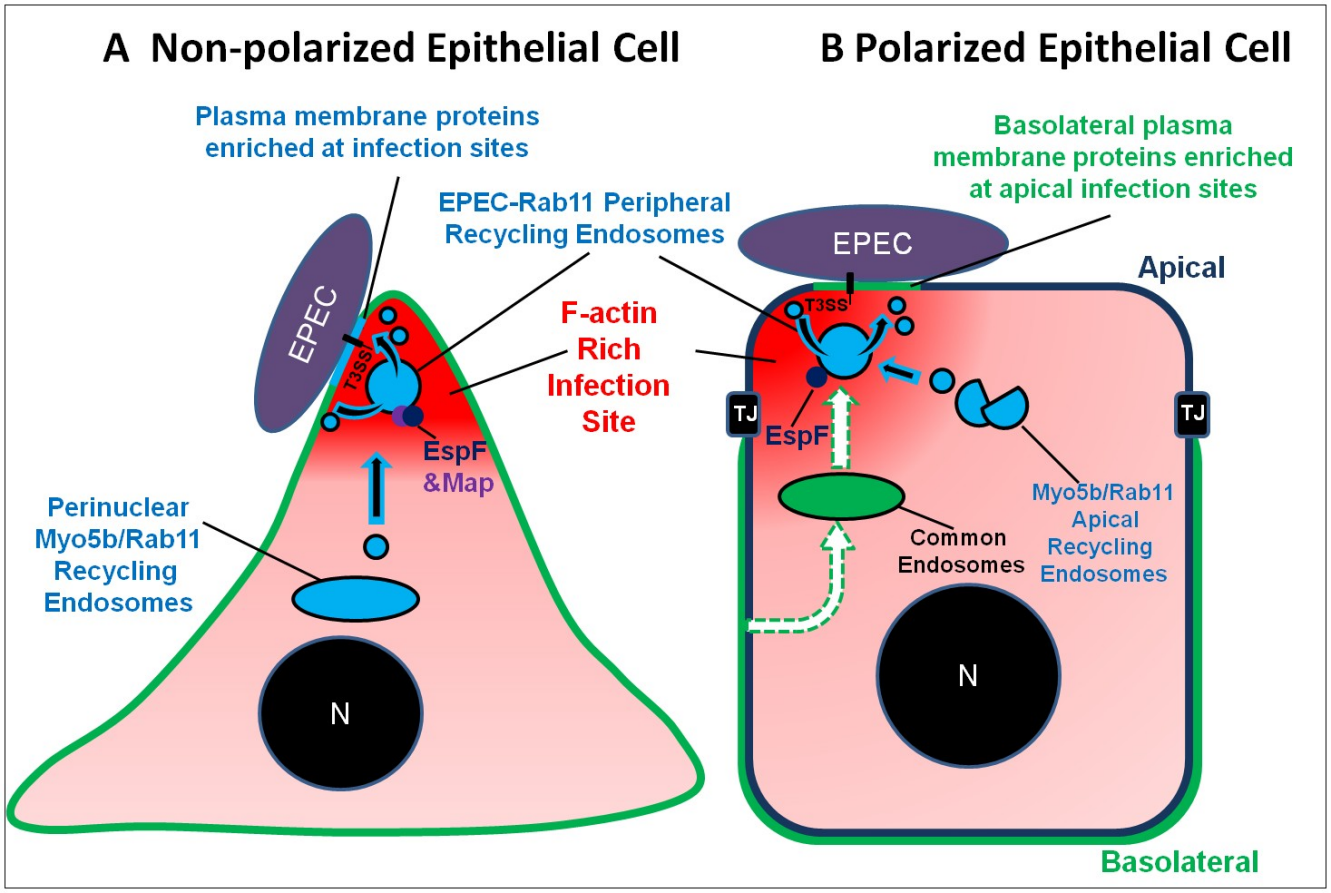
Schematic model of EPEC-induced recruitment of TfnR-Rab11-positive endosomes at infection sites of non-polarized (A) and polarized (B) epithelial cells. In non-polarized cells, EPEC, through its effectors EspF and Map, prompts Myo5b-dependent shuttling of Rab11-positive recycling endosomes to the peripheral infected plasma membrane, where some are clustered at bacterial infection sites. These endosomes exert local endocytic and recycling activities, thereby stimulating the endocytic turnover of plasma membrane proteins at the infected plasma membrane. This activity enriches the plasma membrane infection sites with the missorted endosomes and their cargo proteins. In polarized cells, the Rab11-positive apical recycling endosomes are located beneath the infected (apical) plasma membrane, promoting apical recycling of apical plasma membrane protein. They also utilize their apical recycling capacity to promote transcytosis of basolaterally recycled cargo that has been missorted from the common endosomes to the apical recycling endosomes [25]. Upon infection, translocated EspF promotes Myo5b-dependent recruitment of the Rab11-positive apical recycling endosomes to infection sites, where they locally foster endocytosis and recycling (i.e. endocytic turnover). We hypothesize that EspF, and possibly additional protein effectors, interfere with the basolateral recycling sorting machinery. The consequence of this event is missorting of internalized basolateral plasma membrane recycling proteins (e.g. TfnR) from the common endosomes (which mediate basolateral recycling) to the subapical Rab11 recycling endosomes hijacked by EPEC (dashed line arrows). These endosomes then use their apical recycling capacity to translocate the basolateral plasma membrane proteins into apical plasma membrane infection sites, where they undergo continuous endocytosis and apical recycling. This activity enriches the apical plasma membrane infection sites with plasma membrane proteins originally derived from the basolateral surface of the host.

To summarize, our data suggest the following model. In non-polarized cells (Fig 11A), translocated EspF and Map promote the biogenesis and nucleation of early and recycling endosomes in proximity to plasma membrane infection sites. Actin has shown to be important for endosomal membrane remodeling, endosomal dynamics and plasma membrane protein (cargo) recycling (reviewed in [68]). Thus, EspF and Map may achieve their subversive effect through interactions with actin modulators, e.g. Map-mediated activation of Cdc42 [69] and EspF interactions with N-Wasp and cortactin [23,70,71]. The end result of these activities is a local increase in endocytic turnover and enrichment of recycling plasma membrane proteins at the infection sites. In polarized epithelial cells, another layer of complexity is added (Fig 11B). Basolateral recycling plasma membrane cargoes, e.g. TfnR and AQP3 [67], are sorted, likely by EspF and yet unidentified other effector proteins, to Rab11/Myo5b-positive apical recycling endosomes hijacked to the apical infection sites. These endosomes then utilize their apical recycling capacity to target these cargoes to the infected apical plasma membrane. The consequence of this event is misrouting of basolateral plasma membrane proteins to apical recycling endosomes and plasma membrane infection sites. Some of these proteins may give a survival advantage to the pathogen, while others may disturb the host physiology. A future challenge would be to explore the molecular mechanisms by which EspF (and other protein effectors) target the polarized endocytic sorting machinery of the host, and to elucidate how this contributes to bacterial colonization and virulence.

## Materials and methods

### Bacterial strains, antibodies, plasmids and reagents

Bacterial strains, antibodies, plasmids and reagents used in this study are listed in S1–S4 Tables, respectively. Mutation of *map* in the EPEC *espF*::kan strain was done using the λ Red system [72]. The upstream and downstream recombination sequences were PCR amplified from genomic DNA of EPEC wild-type using primer sets 1371–4197 and 1495–4198, respectively (S5 Table). A chloramphenicol cassette was amplified from pKD3 (S3 Table) using primers 1354 and 1355. A DNA fragment of Δ*map*::cam allele was prepared by isothermal assembly of these three PCR fragments [73] followed by electroporation into EPEC *espF*::kan strains containing pKD46 harboring λ Red genes (γ, β and exo). Then, the desired mutants were selected and pKD46 was cured at 42°C. The mutation was verified using PCR with flanking primers and sequencing.

### Bacterial growth, activation and infection

Bacterial growth and pre-activation of their T3SS was performed as described [74]. HeLa cells were infected with activated bacteria (multiplicity of infection ~100) for 30 min at 37 °C in plain DMEM. Polarized epithelial cells (see below) were infected with non-activated bacteria [i.e. bacteria grown in Luria-Bertani mixed 1:50 (v/v) with plain minimal essential medium (MEM)], for 180 min at 37 °C. For HeLa cells infected with EPEC-*espF*+EspF or EPEC-*map*+Map, expression of the protein effectors was induced by adding 0.2mM isopropyl-β-D-thiogalactopyranoside (IPTG) for the last 30 min of the activation step. For polarized MDCK cells infected with these EPEC strains, expression of the protein effectors was induced by introducing 0.2mM IPTG into the medium during the last 60 min of the infection. Deviations from these conditions are indicated in the text. All infections were performed in a CO_2_ incubator (37°C, 5% CO_2_, 90% humidity).

### Cell culture and transfection

HeLa (J. Orly; The Hebrew University of Jerusalem), Madin-Darby canine kidney (MDCK, type II; K. Mostov; University of California, San Francisco), and Caco2-BBe cells (Tet-off; J. Turner; University of Chicago; Harvard Medical School [75]) were cultured as described [13,74]. MDCK-PTR9 cells (K. Mostov, University of California, San Francisco), which stably express the human Tfn and the rabbit polymeric immunoglobulin receptors, have been described [76,77]. MDCK cells stably expressing a green fluorescent protein (GFP)-human TfnR chimera (MDCK-GFP-TfnR; The Hebrew University of Jerusalem) were generated by transfecting MDCK cells with a human GFP-TfnR encoding plasmid (S3 Table) followed by G418 selection. It was determined that ~85% of the transfected GFP-TfnR is expressed on the basolateral surface of the cells. Cell polarity was obtained by seeding the cells on Transwell filters (12-mm, 0.4 μm, #3401; Corning, Acton, MA), as described [13,74]. Transient transfections of HeLa and MDCK cells with plasmids were performed using the TransIT-X2 6000, or the Lipofectamine 2000 systems, following the manufacturers’ instructions. HeLa cells were analyzed 16 hrs after transfection. MDCK cells were seeded on Transwell filters 16 hrs after transfection and analyzed 96 hrs later.

### Transferrin intake

Unless otherwise indicated, polarized epithelial cells were incubated with fluorescently tagged transferrin (Tfn; S2 Table; 5 μg/ml) administered to either the apical [Tfn (apical)] or the basolateral [Tfn (Basal)] medium during the last 60 min of the infection time. HeLa cells were exposed to the fluorescently tagged Tfn (5 μg/ml) during the 30 min infection period.

### Confocal cell imaging

#### Imaging of fixed cells

Immunofluorescence labeling of fixed and permeabilized cells, labeling of F-actin with Texas Red (TR)-phalloidin and labeling of bacteria and cell nuclei with 4’,6-Diamidine-2’-phenylindole dihydrochloride (DAPI) were performed as described [78]. Immunolabeling of non-permeabilized cells was performed by incubating the fixed cells with primary antibodies (S4 Table) for 60 min at 37 °C in PBS+3% fetal bovine serum (FBS), followed by incubation with fluorescently tagged secondary antibodies (S4 Table). Cells were imaged with an Olympus FV-1200 confocal microscope equipped with a 60x /1.42 oil immersion objective, in a sequential mode to avoid bleed-through of fluorophore emissions. The excitation wavelengths were 488nm, 561nm and 633nm, and the emission filter passbands were 505-525nm, 575-625nm and 650-720nm for green, red and far-red fluorescence respectively. In all experiments, wide-field differential interference contrast (DIC) images were acquired to visualize cell-adhered bacteria. Notably, however, these images are shown only when infecting microcolonies were visualized more clearly than DAPI staining. Z-stacks consisting of optical sections covering the entire cell height were acquired at 0.5 μm spacing. Images were analyzed by FIJI (NIH). Quantitative analysis of fluorescence levels at infection sites was measured in relation to uninfected areas, and termed “% Change of Uninfected”, as described [78]. Analysis of the apical-basal distribution (Z-plots) of F-actin and surface TfnR in polarized MDCK and Caco2-BBe cell monolayers was performed as described [78].

#### Live cell imaging of polarized MDCK cells

Cells were seeded on the underside, i.e. in an “apical side-down” inverted orientation of translucent Transwell filters (12mm, 0.4 μm, #3460, Corning, Acton, MA) for 4 days, as described [79]. Transwell filters were then placed on a glass jar with the apical surface of the cells facing upwards. A drop of 200 μl of warm infection medium [overnight grown bacteria mixed 1:50 v/v with plain MEM (Earl’s salts)] was placed on the apical surface of the cells, which were subsequently incubated for 120 min at 37 °C in a CO_2_ incubator (5% CO_2_; 90% humidity). Cells on filters were then placed in a 12-well plate and incubated for an additional 60 min at 37°C in the presence of plain MEM bathing their apical and basolateral surface. Thereafter, filters were mounted on a coverslip with the cells facing the objective lens, as described [79]. Single time point confocal imaging was performed, and the degree of protein recruitment at bacterial infection sites was determined as described above.

### Transcytosis and basolateral recycling assay

The basolateral surface of polarized MDCK-PTR9 cells was initially exposed to Tfn-AF488 (10 μg/ml) for 90 min at 37°C. Cells were then washed extensively with plain DMEM lacking phenol red (37°C). The apical surface of these cells was infected with pre-activated EPEC (in DMEM lacking phenol red) for 120 min, or left uninfected. The basolateral and apical media of the cells were collected and stored at 4°C. Cells were washed and Texas-Red (TR)-Dextran [70 KDa; 0.5mg/ml in cold (4 °C) DMEM lacking phenol red] was added to their apical surface, while the basolateral surface was kept in plain DMEM lacking phenol red. Cells were incubated for 60 min at 4°C, and the apical and basal media were collected. The fluorescence levels of Tfn-AF488 released to the apical (i.e. transcytosed) or to the basal (recycled) medium and the fluorescence levels of TR-Dextran present in the basolateral medium (cell monolayer leakiness) were determined by the Synergy H1, Hybrid Multi-mode Microplate Reader (BioTek, Winooski, VT), and presented as % change of uninfected cells.

### Biochemical-based assay for monitoring Tfn endocytosis

Polarized MDCK-PTR9 cells were washed and incubated in warm (37°C) MEM containing 1% BSA for 60 min to remove cell-associated Tfn. Cells were then infected for 180 min with EPEC, or left uninfected. Unlabeled human holo-Tfn was added to the apical or basal media of the cells for the last 60 min of the infection period. The surface-bound ligand was stripped off by extensive washes in ice-cold PBS followed by cell incubation in stripping buffer [50mM 2-(N-morpholino) ethanesulfonic acid (MES; pH = 5.0), 200 mM NaCl, 100 mM deferoxamine mesylate salt] for 30 min at 17°C. Cells were lysed in ice-cold lysis buffer [10 mM Tris-HCl pH8.0, 1% v/v Triton-X100, 150 mM NaCl, 5mM EDTA, protease inhibitors cocktail], and shacked by vortex for 30 min at 4°C. Detergent-insoluble materials were removed by centrifugation (16,000 g, 10 min, 4°C). Cell lysates were analyzed for the presence of Tfn by SDS-PAGE followed by Western blotting, using rabbit anti-human Tfn antibody (S4 Table). In some experiments, the dynamin inhibitor Dynasore (80 μM) was applied apically for 60 min prior to cell exposure to Tfn, and together with Tfn during the infection period.

### Flow cytometry-based assay for monitoring endocytosis, recycling and surface-bound Tfn levels

#### Endocytosis assay

Polarized epithelial cells were washed and incubated in warm (37°C) MEM containing 1% BSA for 60 min to remove cell-associated Tfn. Cells were then infected with EPEC and exposed to Tfn-AF647 (apically or basally) for the last 60 min of infection. Surface-bound ligand was stripped off as before. Cells were then trypsinized (Trypsin A, 10 min, 37 °C), washed twice by centrifugation and kept in ‘fluorescence-activated cell sorter (FACS) buffer’ [PBS + 2% fetal bovine serum (FBS)] until measurement. HeLa cells were incubated in warm (37°C) DMEM containing 1% BSA for 60 min, and then infected with EPEC for 30 min concomitant to Tfn-AF647 exposure. Subsequently, cells were washed (3X) with ice-cold PBS and detached by Trypsin C treatment for 1 min at 37°C. Cells were further washed twice by centrifugation in ice-cold PBS and surface-bound ligand was stripped off by incubation in stripping buffer for 30 min at 17°C. Cells were then washed in ice-cold PBS and kept in ice-cold FACS buffer until measurement. Propidium iodide (PI; 10 μg/ml) was added to all samples ~ 3 min prior to the measurement. Inhibition of Tfn endocytosis was achieved by pre-treating cells with Dynasore (80 μM), or Dyngo (20 μM), for 60 min at 37°C, and throughout the infection time. The amount of cell-associated Tfn in PI-negative cells was determined by flow cytometry. The mean fluorescence intensity value of infected (MFIi) and uninfected (MFIu) cells was determined and data were presented as % change of uninfected cells.

$$\%\;Change\;of\;Uninfected\;Cells=(\frac{{MFI}_{i}-{MFI}_{u}}{{MFI}_{u}})x100$$

#### Recycling assay

Two sets of infected and two sets of uninfected cells were allowed to internalize Tfn-AF647, as before. Cells were then subjected to extensive washes with ice-cold PBS to remove unbound ligand. Subsequently, one set of cells was incubated in chase medium (MEM containing 1% BSA, 20 μg/ml unlabeled holo-Tfn and 100 mM deferoxamine mesylate salt) for 90 (MDCK) or 30 (HeLa) min at 37°C, while the other set was untreated and kept on ice. All samples were washed with ice-cold PBS, surface-bound ligand was stripped-off and cells were subjected to FACS analysis. The MFI of cells following ligand chase was subtracted from the MFI of cells subjected to ligand endocytosis; the value obtained represents the amount of ligand that was recycled out (i.e. released) from the cells. Results were expressed as % change of uninfected cells. In both assays, experiments that examined the effects of specific treatments of uninfected cells were expressed as % change of control cells.

#### Determining cell surface TfnR (s-TfnR) levels

HeLa cells were infected for 30 min or left uninfected. Cells were washed four times with ice-cold PBS and exposed to 20 μg/ml Tfn-AF647 for 30 min at 4°C. Cells were then washed with ice-cold PBS to remove unbound ligand, detached by trypsinization, washed and suspended in ice-cold FACS buffer.

#### Flow cytometry

Flow cytometry analysis was performed using a FACS Aria III flow cytometer-sorter (BD Biosciences, San Jose, CA) equipped with 4 lasers (405, 488, 561, 633 nm). All flow cytometry data were analyzed with FACSDiva software (BD Biosciences). Fluorophores were detected using the default detector arrays supplied with the instrument. Voltage settings for SSC, FSC and fluorescence channels were kept constant for all experiments described. Data were recorded for ≥ 30,000 cells from each sample.

### Analysis of perinuclear and peripheral endosomes

#### Determining the area of perinuclear endosomes

HeLa cells were exposed to Tfn-AF647 (5 μg/ml) during EPEC infection, or left uninfected and exposed to Tfn-AF647. Cells were then fixed and imaged by confocal microscopy. The mean fluorescence projection encompassing the entire cell volume (dark-to-dark) was generated and cell borders were marked manually. The distribution of fluorescent ligand within these borders was identified by the ‘Otsu Threshold’ and the area occupied by the perinuclear recycling endosome was measured using the ‘Particle Analysis’ tool of FIJI (NIH). The distribution of area values obtained was displayed in a box and whisker diagram, using the ‘box plot tool’ (http://www.physics.csbsju.edu/stats/display.distribution.html).

#### Determining the fluorescence intensity of peripheral endosomes

HeLa cells were exposed to Tfn-AF647 during EPEC infection, fixed, and imaged by confocal microscopy. In all experiments, confocal scanning was performed under the same conditions. Peripheral endosomes were defined as fluorescent particles residing between the cell edge (selected manually) and the perinuclear recycling endosome punctum identified by ‘Otsu Threshold’. In this region, the noise signal in individual confocal image Z-slices (0.5μm thick) was filtered by the ‘Mean filter’ tool (radius, 1.0 pixel) and the fluorescence intensity threshold (100–1200 AU for TfnR and 100–1000 AU for EEA1) was set to levels allowing visualization of distinct particles (endosomes). Under these conditions, the smallest (0.2μm^2^) and the largest (2.0 μm^2^) visually distinct particles (i.e. endosomes) were identified and converted to Regions Of Interest (ROI) by the FIJI ‘Particle Analysis’ tool, under restriction for size (0.2–2.0 μm^2^) and for particle circularity (0.5–1.0). The signal intensity of individual Tfn, TfnR and EEA1 particles was then measured in unprocessed individual confocal image Z-slices by the FIJI ‘Particle Analysis’ tool. In EPEC-*wt* infected cells, some endosomes recruited to infection sites were excluded because they failed to meet the 2.0 μm^2^ upper limit and showed poor circularity.

### Photo-conversion and live imaging

HeLa cells (20,000 cells) were seeded on ibiTreat μ-slide 8 well plates (Ibidi, Martinsried, Germany). One day after seeding, cells were transfected with tdEos-Rab11a (S3 Table). Sixteen hrs post-transfection cells were washed with warm PBS, and exposed to a 1:1:1 mixture of DMEM, activated EPEC and Tfn-AF647 (100 μl each) (S2 Table; 5μg/ml). Perinuclear Rab11a/Tfn positive puncta were selected and irreversibly photo-converted by exposure to a 405nm laser beam for 30 sec. Cells were then subjected to time-lapse confocal live cell imaging. Quantitative analysis of fluorescence levels at infection sites was measured relative to uninfected areas, and termed “% change of uninfected cells”.

### Electron microscopy

HeLa cells were infected with EPEC in the presence of Tfn-HRP (5 μg/ml; S2 Table). Cells were rinsed in DPBS and fixed for 30 min in freshly prepared fixative containing 4% paraformaldehyde and 0.25% glutaraldehyde (Electron Microscopy Sciences, Fort Washington, PA, USA) in 0.1 M phosphate buffer (pH 7.4). After thorough washings, samples were treated with 3,3' diaminobenzidine tetrahydrochloride (DAB, 5 mg/20 ml PBS supplemented with 4 μl H_2_O_2_) for 10 min to visualize the HRP reaction product. The DAB product was further enhanced and substituted with silver/gold particles, as described [80]. Finally, the samples were postfixed in a mixture of 1% osmium tetroxide and 1.5% potassium ferricyanide in 0.1 M cacodylate buffer pH 7.0, dehydrated in ascending concentrations of ethanol and embedded in EM-BED812 (Electron Microscopy Sciences, Hatfield, PA). Ultrathin sections were lightly stained with uranyl acetate and lead citrate and examined with a Tecnai-12 TEM 100kV (Phillips, Eindhoven, the Netherlands) electron microscope equipped with MegaView II CCD camera and Analysis version 3.0 software (SoftImaging System GmbH, Münstar, Germany).

### Rab11 silencing by siRNA

HeLa cells (~ 50% confluence) were transfected with si-Rab11a (50 nM; S2 Table), or si-Rab11b (50 nM; S2 Table), or Rab11a+b (25 nM each) for 72 hours, using the TransIT-X2 6000 system. Non-Targeting siRNA Pool #2 (50 nM; S2 Table) was used as control. Rab11 silencing was confirmed by Western blotting, using anti-Rab11a and anti-Rab11b antibodies (S4 Table).

### Co-immunoprecipitation analyzed by mass spectometry and Western blotting

#### Co-immunoprecipitation

HeLa cells grown on a Nunclon Delta Treated Square BioAssay Dish (Nunc; #166508, ThermoFisher Scientific, Roskilde, Denmark) to 70% confluence were infected with preactivated (3.5 hrs, IPTG added to the last 30 min) EPEC *espF* or *espF*+EspF for 30 mins at 37 °C. Cells were washed with ice-cold DPBS and lysed in lysis buffer [50mM Tris (pH 7.4), 150mM NaCl, 0.5% NP-40)] supplemented with protease inhibitors cocktail, phosphatase inhibitors cocktail and 1 mM NaVO_4_. Lysates were subjected to immunoprecipitation using anti-FLAG-M2 magnetic beads (Sigma, M8823) for 4 hrs at 4°C with end-to-end rotation. The beads were then washed five times with lysis buffer and three times with lysis buffer without NP-40. Beads were dried using a Hamilton syringe and subjected to proteomic analysis by mass-spectrometry (MS), as described below. The experiment was performed in quadruplicate.

Sample preparation for MS analysis: Beads were washed twice with 25 mM Tris-HCl pH 8.0. The packed beads were resuspended in 100 μl 8M urea, 10 mM DTT, 25 mM Tris-HCl pH 8.0 and incubated for 30 min at 22°C. Next, Iodoacetamide (55 mM) was added and beads were incubated for 30 min (22°C, in the dark), followed by addition of DTT (20 mM). The sample was diluted by the addition of 6 volumes of 25 mM Tris-HCl pH 8.0. Trypsin (0.25 μg) was added and the beads were incubated overnight at 37°C with gentle agitation. The beads were spun down and the supernatant was loaded on C18 Stage tips to desalt the released peptides, as described in [81]. Two-thirds of the eluted peptides were used for MS analysis.

#### nanoLC-MS/MS analysis

MS analysis was performed using a Q Exactive Plus mass spectrometer (Thermo Fisher Scientific, Waltham, MA USA) coupled on-line to a nanoflow UHPLC instrument, Ultimate 3000 Dionex (Thermo Fisher Scientific, Waltham, MA USA). Eluted peptides were separated over a 60 min gradient run at a flow rate of 0.3 μl/min on a reverse phase 25-cm-long C18 column (75 μm ID, 2 μm, 100Å, Thermo PepMapRSLC). The survey scans (380–2,000 m/z, target value 3E6 charges, maximum ion injection times 50 ms) were acquired and followed by higher energy collisional dissociation (HCD) based fragmentation (normalized collision energy 25). A resolution of 70,000 was used for survey scans and up to 15 dynamically chosen most abundant precursor ions, with “peptide preferable” profile were fragmented (isolation window 1.6 m/z). The MS/MS scans were acquired at a resolution of 17,500 (target value 1E5 charges, maximum ion injection times 120 ms). Dynamic exclusion was 60 sec. Data were acquired using Xcalibur software (Thermo Scientific). To avoid a carryover of the peptides between the samples, the column was washed with 80% acetonitrile, 0.1% formic acid for 40 min.

#### MS data analysis

Mass spectra data were processed using the MaxQuant computational platform, version 1.5.3.12 [82]. Peak lists were searched against the *Homo sapiens* and *Escherichia coli O127* Uniprot FASTA sequence databases containing respectively a total of 26,199 and 772 reviewed entries. The search included cysteine carbamidomethylation as a fixed modification and oxidation of methionine as variable modifications and allowed up to two miscleavages. Peptides with a length of at least seven amino-acids were considered and the required FDR was set to 1% at the peptide and protein level. Protein identification required at least 6 unique or razor peptides per protein group (four replicated in each group). Relative protein quantification in MaxQuant was performed using the label-free quantification (LFQ) algorithm [83]. Protein contaminations and proteins identified by less than 2 peptides were excluded from the analysis. Moreover, only proteins identified in at least two repeats were considered for the analysis. To identify a set of EspF interacting proteins, student t-test was carried out together with the ratio of the median LFQ values. Proteins which were not detected in the control sample or enriched by 80 fold with p-value <0.05 were considered as EspF binders. To visualize the protein interaction network we used the STRING server (http://string-db.org/) and the Cytoscape software [84].

#### Analysis by Western blotting

Co-immunoprecipitation experiments were performed essentially as above. Cells grown on five 150x20mm plates (Nunc, #168381, ThermoFisher Scientific, Roskilde, Denmark) were infected with the EPEC-*espF*, or EPEC-*espF*+EspF, or EPEC-*espF*+EspF mod-wt or EPEC-*espF*+EspF mod-RD strains. EspF was immunoprecipitated with anti-FLAG antibodies and associated SNX proteins were detected by SDS-PAGE and Western blotting. Band intensity was determined, as described [78].

### Statistical analysis

Results are presented as means ± standard error (SE). Statistical significance was determined by two-tailed Student’s t-test. A p-value < 0.05 indicates a statistically significant difference. ***p<0.0005, ** p<0.005, * p<0.05; ns = statistically not significant, p>0.05.

## Supporting information

S1 Fig T3SS-dependent recruitment of early and recycling endocytic elements at apical infection sites. (TIF)

S2 Fig T3SS-dependent recruitment of basally or apically internalized Tfn/TfnR, to apical infection sites. (TIF)

S3 Fig T3SS-dependent recruitment of Tfn, TfnR, SH3BP4 and ACAP1 in polarized MDCK cells. (TIF)

S4 Fig Type III secreted elements elicit the recruitment of endocytic markers to infection sites. (TIF)

S5 Fig Dynamic recruitment of early and recycling endocytic markers at infection sites. (TIF)

S6 Fig Unlike Tfn, fluid-phase (Dextran), lysosomal (LysoTracker), and late endosomal (Rab7a) markers are not recruited at EPEC-*wt* infection sites. (TIF)

S7 Fig T3SS-dependent recruitment of β1-Integrin at infection sites. (TIF)

S8 Fig Type III secreted elements elicit increased endocytic turnover in non-polar cells. (TIF)

S9 Fig Endocytic activity is not essential for the recruitment of Tfn/Rab11a positive recycling endosomes at infection sites. (TIF)

S10 Fig Myo5b is essential for Rab11-dependent TfnR trafficking to the cell surface. (TIF)

S11 Fig Knockdown of Rab11a and Rab11b by siRNA redistributes the Tfn-positive endosomes to the cell periphery and increases Tfn endocytic turnover. (TIF)

S12 Fig EspF and Map mediate the recruitment of Tfn/TfnR and Myo5b/Rab11a at infection sites. (TIF)

S13 Fig EspF and Map translocation and localization in the host. (TIF)

S14 Fig Tfn/Iron-dependent increase in host cell surface colonization. (TIF)

S15 Fig T3SS-dependent recruitment of aquaporins 2 and 3 to infection sites. (TIF)

S1 Table EPEC strains. (DOCX)

S2 Table Reagents. (DOCX)

S3 Table Expression constructs. (DOCX)

S4 Table Antibodies. (DOCX)

S5 Table Primers. (DOCX)

S6 Table Dataset (mass spectrometry). (XLSX)

S1 Movie HeLa cells co-expressing mRFP-LifeAct and GFP-TfnR were exposed to Tfn-DL649 and infected with EPEC-*escV*. (MOV)

S2 Movie HeLa cells co-expressing mRFP-LifeAct and GFP-TfnR were infected with EPEC-*wt*, concomitantly exposed to Tfn-DL649 and subjected to time-lapse confocal imaging. (MOV)

S3 Movie HeLa cells co-expressing mRFP-LifeAct and GFP-Rab5a were infected with EPEC-*escV*, concomitantly exposed to Tfn-DL649 and subjected to time-lapse confocal imaging. (MOV)

S4 Movie HeLa cells co-expressing mRFP-LifeAct and GFP-Rab5a were infected with EPEC-*wt*, concomitantly exposed to Tfn-DL649 and subjected to time-lapse confocal imaging. (MOV)

S5 Movie HeLa cells co-expressing mRFP-LifeAct and GFP-Rab11a were infected with EPEC-*escV*, concomitantly exposed to Tfn-DL649 and subjected to time-lapse confocal imaging. (MOV)

S6 Movie HeLa cells co-expressing mRFP-LifeAct and GFP-Rab11a were infected with EPEC-*wt*, concomitantly exposed to Tfn-DL649 and subjected to time-lapse confocal imaging. (MOV)

S7 Movie HeLa cells expressing tdEos-Rab11a were pre-exposed to Tfn-AF647, infected with EPEC-*escV*, photoconverted, and subjected to time-lapse confocal imaging. (MOV)

S8 Movie HeLa cells expressing tdEos-Rab11a were pre-exposed to Tfn-AF647, infected with EPEC-*wt*, photoconverted, and subjected to time-lapse confocal imaging. (MOV)

S1 Text Supporting methods. (DOCX)

## References

[1] Nataro JP, Kaper JB (1998) Diarrheagenic Escherichia coli. Clin Microbiol Rev 11: 142–201. 9457432 10.1128/cmr.11.1.142 PMC121379

[2] Caron E, Crepin VF, Simpson N, Knutton S, Garmendia J, et al (2006) Subversion of actin dynamics by EPEC and EHEC. Curr Opin Microbiol 9: 40–45. 10.1016/j.mib.2005.12.008 16406772

[3] Wong AR, Pearson JS, Bright MD, Munera D, Robinson KS, et al (2011) Enteropathogenic and enterohaemorrhagic Escherichia coli: even more subversive elements. Molecular microbiology 80: 1420–1438. 10.1111/j.1365-2958.2011.07661.x 21488979

[4] Pinaud L, Sansonetti PJ, Phalipon A (2018) Host Cell Targeting by Enteropathogenic Bacteria T3SS Effectors. Trends in microbiology. 10.1016/j.tim.2018.01.010 29477730

[5] Aroeti B, Friedman G, Zlotkin-Rivkin E, Donnenberg MS (2012) Retraction of enteropathogenic E. coli type IV pili promotes efficient host cell colonization, effector translocation and tight junction disruption. Gut microbes 3: 267–271. 10.4161/gmic.19814 22572833 PMC3427219

[6] Finlay BB, Abe A (1998) Enteropathogenic E. coli interactions with host cells. Jpn J Med Sci Biol 51 Suppl: S91–100. 10211441 10.7883/yoken1952.51.supplement1_s91

[7] Iguchi A, Thomson NR, Ogura Y, Saunders D, Ooka T, et al (2009) Complete genome sequence and comparative genome analysis of enteropathogenic Escherichia coli O127:H6 strain E2348/69. J Bacteriol 191: 347–354. 10.1128/JB.01238-08 18952797 PMC2612414

[8] Dean P, Kenny B (2009) The effector repertoire of enteropathogenic E. coli: ganging up on the host cell. Curr Opin Microbiol 12: 101–109. 10.1016/j.mib.2008.11.006 19144561 PMC2697328

[9] Mills E, Baruch K, Charpentier X, Kobi S, Rosenshine I (2008) Real-time analysis of effector translocation by the type III secretion system of enteropathogenic Escherichia coli. Cell host & microbe 3: 104–113. 18312845 10.1016/j.chom.2007.11.007

[10] Ugalde-Silva P, Gonzalez-Lugo O, Navarro-Garcia F (2016) Tight Junction Disruption Induced by Type 3 Secretion System Effectors Injected by Enteropathogenic and Enterohemorrhagic Escherichia coli. Frontiers in cellular and infection microbiology 6: 87 10.3389/fcimb.2016.00087 27606286 PMC4995211

[11] Zhuang X, Chen Z, He C, Wang L, Zhou R, et al (2017) Modulation of host signaling in the inflammatory response by enteropathogenic Escherichia coli virulence proteins. Cellular & molecular immunology 14: 237–244. 27796284 10.1038/cmi.2016.52 PMC5360883

[12] Pinaud L, Sansonetti PJ, Phalipon A (2018) Host Cell Targeting by Enteropathogenic Bacteria T3SS Effectors. Trends in microbiology 26: 266–283. 10.1016/j.tim.2018.01.010 29477730

[13] Sason H, Milgrom M, Weiss AM, Melamed-Book N, Balla T, et al (2009) Enteropathogenic Escherichia coli subverts phosphatidylinositol 4,5-bisphosphate and phosphatidylinositol 3,4,5-trisphosphate upon epithelial cell infection. Mol Biol Cell 20: 544–555. 10.1091/mbc.E08-05-0516 18987340 PMC2613080

[14] Unsworth KE, Mazurkiewicz P, Senf F, Zettl M, McNiven M, et al (2007) Dynamin is required for F-actin assembly and pedestal formation by enteropathogenic Escherichia coli (EPEC). Cell Microbiol 9: 438–449. 10.1111/j.1462-5822.2006.00801.x 16965516

[15] Lin AE, Benmerah A, Guttman JA (2011) Eps15 and Epsin1 are crucial for enteropathogenic Escherichia coli pedestal formation despite the absence of adaptor protein 2. The Journal of infectious diseases 204: 695–703. 10.1093/infdis/jir386 21810914

[16] Bonazzi M, Vasudevan L, Mallet A, Sachse M, Sartori A, et al (2011) Clathrin phosphorylation is required for actin recruitment at sites of bacterial adhesion and internalization. The Journal of cell biology 195: 525–536. 10.1083/jcb.201105152 22042622 PMC3206339

[17] Guttman JA, Lin AE, Veiga E, Cossart P, Finlay BB (2010) Role for CD2AP and other endocytosis-associated proteins in enteropathogenic Escherichia coli pedestal formation. Infection and immunity 78: 3316–3322. 10.1128/IAI.00161-10 20515931 PMC2916276

[18] Veiga E, Guttman JA, Bonazzi M, Boucrot E, Toledo-Arana A, et al (2007) Invasive and adherent bacterial pathogens co-Opt host clathrin for infection. Cell Host Microbe 2: 340–351. 10.1016/j.chom.2007.10.001 18005755 PMC2803069

[19] Tapia R, Kralicek SE, Hecht GA (2017) EPEC effector EspF promotes Crumbs3 endocytosis and disrupts epithelial cell polarity. Cellular microbiology. 10.1111/cmi.12757 PMC5645036 28618099

[20] Furniss RC, Slater S, Frankel G, Clements A (2016) Enterohaemorrhagic E. coli modulates an ARF6:Rab35 signaling axis to prevent recycling endosome maturation during infection. J Mol Biol 428: 3399–3407. 10.1016/j.jmb.2016.05.023 27261256 PMC5013874

[21] Clements A, Stoneham CA, Furniss RC, Frankel G (2014) Enterohaemorrhagic Escherichia coli inhibits recycling endosome function and trafficking of surface receptors. Cell Microbiol 16: 1693–1705. 10.1111/cmi.12319 24898821 PMC4336558

[22] Marches O, Batchelor M, Shaw RK, Patel A, Cummings N, et al (2006) EspF of enteropathogenic Escherichia coli binds sorting nexin 9. J Bacteriol 188: 3110–3115. 10.1128/JB.188.8.3110-3115.2006 16585770 PMC1447016

[23] Alto NM, Weflen AW, Rardin MJ, Yarar D, Lazar CS, et al (2007) The type III effector EspF coordinates membrane trafficking by the spatiotemporal activation of two eukaryotic signaling pathways. J Cell Biol 178: 1265–1278. 10.1083/jcb.200705021 17893247 PMC2064658

[24] Cossart P, Veiga E (2008) Non-classical use of clathrin during bacterial infections. J Microsc 231: 524–528. 10.1111/j.1365-2818.2008.02065.x 18755008

[25] Bay AE, Schreiner R, Rodriguez-Boulan E (2015) Structural and functional analysis of endosomal compartments in epithelial cells. Methods in cell biology 130: 271–288. 10.1016/bs.mcb.2015.06.019 26360040 PMC5755384

[26] Wang E, Pennington JG, Goldenring JR, Hunziker W, Dunn KW (2001) Brefeldin A rapidly disrupts plasma membrane polarity by blocking polar sorting in common endosomes of MDCK cells. J Cell Sci 114: 3309–3321. 11591819 10.1242/jcs.114.18.3309

[27] Tosoni D, Puri C, Confalonieri S, Salcini AE, De Camilli P, et al (2005) TTP specifically regulates the internalization of the transferrin receptor. Cell 123: 875–888. 10.1016/j.cell.2005.10.021 16325581

[28] Dai J, Li J, Bos E, Porcionatto M, Premont RT, et al (2004) ACAP1 promotes endocytic recycling by recognizing recycling sorting signals. Developmental cell 7: 771–776. 10.1016/j.devcel.2004.10.002 15525538

[29] Hoekstra D, Tyteca D, van ISC (2004) The subapical compartment: a traffic center in membrane polarity development. Journal of cell science 117: 2183–2192. 10.1242/jcs.01217 15126620

[30] Lapierre LA, Kumar R, Hales CM, Navarre J, Bhartur SG, et al (2001) Myosin vb is associated with plasma membrane recycling systems. Molecular biology of the cell 12: 1843–1857. 10.1091/mbc.12.6.1843 11408590 PMC37346

[31] Ducharme NA, Ham A-JL, Lapierre LA, Goldenring JR (2011) Rab11-FIP2 influences multiple components of the endosomal system in polarized MDCK cells. Cellular logistics 1: 57–68. 10.4161/cl.1.2.15289 21686255 PMC3116584

[32] Schafer JC, Baetz NW, Lapierre LA, McRae RE, Roland JT, et al (2014) Rab11-FIP2 interaction with MYO5B regulates movement of Rab11a-containing recycling vesicles. Traffic 15: 292–308. 10.1111/tra.12146 24372966 PMC4081500

[33] Muza-Moons MM, Koutsouris A, Hecht G (2003) Disruption of cell polarity by enteropathogenic Escherichia coli enables basolateral membrane proteins to migrate apically and to potentiate physiological consequences. Infect Immun 71: 7069–7078. 10.1128/IAI.71.12.7069-7078.2003 14638797 PMC308921

[34] Mayle KM, Le AM, Kamei DT (2012) The intracellular trafficking pathway of transferrin. Biochimica et biophysica acta 1820: 264–281. 10.1016/j.bbagen.2011.09.009 21968002 PMC3288267

[35] Jing J, Tarbutton E, Wilson G, Prekeris R (2009) Rab11-FIP3 is a Rab11-binding protein that regulates breast cancer cell motility by modulating the actin cytoskeleton. European journal of cell biology 88: 325–341. 10.1016/j.ejcb.2009.02.186 19327867 PMC2673714

[36] Veale KJ, Offenhauser C, Whittaker SP, Estrella RP, Murray RZ (2010) Recycling endosome membrane incorporation into the leading edge regulates lamellipodia formation and macrophage migration. Traffic 11: 1370–1379. 10.1111/j.1600-0854.2010.01094.x 20604897

[37] Simonsen A, Lippe R, Christoforidis S, Gaullier JM, Brech A, et al (1998) EEA1 links PI(3)K function to Rab5 regulation of endosome fusion. Nature 394: 494–498. 10.1038/28879 9697774

[38] Ward ES, Martinez C, Vaccaro C, Zhou J, Tang Q, et al (2005) From sorting endosomes to exocytosis: association of Rab4 and Rab11 GTPases with the Fc receptor, FcRn, during recycling. Molecular biology of the cell 16: 2028–2038. 10.1091/mbc.E04-08-0735 15689494 PMC1073680

[39] Sonnichsen B, De Renzis S, Nielsen E, Rietdorf J, Zerial M (2000) Distinct membrane domains on endosomes in the recycling pathway visualized by multicolor imaging of Rab4, Rab5, and Rab11. The Journal of cell biology 149: 901–914. 10.1083/jcb.149.4.901 10811830 PMC2174575

[40] Newton AJ, Kirchhausen T, Murthy VN (2006) Inhibition of dynamin completely blocks compensatory synaptic vesicle endocytosis. Proceedings of the National Academy of Sciences of the United States of America 103: 17955–17960. 10.1073/pnas.0606212103 17093049 PMC1693854

[41] Roland JT, Bryant DM, Datta A, Itzen A, Mostov KE, et al (2011) Rab GTPase-Myo5B complexes control membrane recycling and epithelial polarization. Proceedings of the National Academy of Sciences of the United States of America 108: 2789–2794. 10.1073/pnas.1010754108 21282656 PMC3041130

[42] Takahashi S, Kubo K, Waguri S, Yabashi A, Shin HW, et al (2012) Rab11 regulates exocytosis of recycling vesicles at the plasma membrane. Journal of cell science 125: 4049–4057. 10.1242/jcs.102913 22685325

[43] Creasey EA, Delahay RM, Bishop AA, Shaw RK, Kenny B, et al (2003) CesT is a bivalent enteropathogenic Escherichia coli chaperone required for translocation of both Tir and Map. Mol Microbiol 47: 209–221. 12492865 10.1046/j.1365-2958.2003.03290.x

[44] Alto NM, Shao F, Lazar CS, Brost RL, Chua G, et al (2006) Identification of a bacterial type III effector family with G protein mimicry functions. Cell 124: 133–145. 10.1016/j.cell.2005.10.031 16413487

[45] Huang Z, Sutton SE, Wallenfang AJ, Orchard RC, Wu X, et al (2009) Structural insights into host GTPase isoform selection by a family of bacterial GEF mimics. Nat Struct Mol Biol 16: 853–860. 10.1038/nsmb.1647 19620963 PMC5130228

[46] Orchard RC, Kittisopikul M, Altschuler SJ, Wu LF, Suel GM, et al (2012) Identification of F-actin as the dynamic hub in a microbial-induced GTPase polarity circuit. Cell 148: 803–815. 10.1016/j.cell.2011.11.063 22341450 PMC3368334

[47] Simpson N, Shaw R, Crepin VF, Mundy R, FitzGerald AJ, et al (2006) The enteropathogenic Escherichia coli type III secretion system effector Map binds EBP50/NHERF1: implication for cell signalling and diarrhoea. Mol Microbiol 60: 349–363. 10.1111/j.1365-2958.2006.05109.x 16573685

[48] D’Souza RS, Semus R, Billings EA, Meyer CB, Conger K, et al (2014) Rab4 orchestrates a small GTPase cascade for recruitment of adaptor proteins to early endosomes. Curr Biol 24: 1187–1198. 10.1016/j.cub.2014.04.003 24835460 PMC4059052

[49] Schonteich E, Wilson GM, Burden J, Hopkins CR, Anderson K, et al (2008) The Rip11/Rab11-FIP5 and kinesin II complex regulates endocytic protein recycling. J Cell Sci 121: 3824–3833. 10.1242/jcs.032441 18957512 PMC4365997

[50] Cox D, Lee DJ, Dale BM, Calafat J, Greenberg S (2000) A Rab11-containing rapidly recycling compartment in macrophages that promotes phagocytosis. Proceedings of the National Academy of Sciences of the United States of America 97: 680–685. 10.1073/pnas.97.2.680 10639139 PMC15390

[51] Cullen PJ (2008) Endosomal sorting and signalling: an emerging role for sorting nexins. Nature reviews Molecular cell biology 9: 574–582. 10.1038/nrm2427 18523436

[52] Willenborg C, Jing J, Wu C, Matern H, Schaack J, et al (2011) Interaction between FIP5 and SNX18 regulates epithelial lumen formation. The Journal of cell biology 195: 71–86. 10.1083/jcb.201011112 21969467 PMC3187708

[53] Ellis S, Mellor H (2000) Regulation of endocytic traffic by rho family GTPases. Trends in cell biology 10: 85–88. 10675900 10.1016/s0962-8924(99)01710-9

[54] Stamnes M (2002) Regulating the actin cytoskeleton during vesicular transport. Current opinion in cell biology 14: 428–433. 12383793 10.1016/s0955-0674(02)00349-6

[55] Francis MK, Holst MR, Vidal-Quadras M, Henriksson S, Santarella-Mellwig R, et al (2015) Endocytic membrane turnover at the leading edge is driven by a transient interaction between Cdc42 and GRAF1. Journal of cell science 128: 4183–4195. 10.1242/jcs.174417 26446261 PMC4712783

[56] Cao TT, Deacon HW, Reczek D, Bretscher A, von Zastrow M (1999) A kinase-regulated PDZ-domain interaction controls endocytic sorting of the beta2-adrenergic receptor. Nature 401: 286–290. 10.1038/45816 10499588

[57] Skaar EP (2010) The battle for iron between bacterial pathogens and their vertebrate hosts. PLoS Pathog 6: e1000949 10.1371/journal.ppat.1000949 20711357 PMC2920840

[58] Siburt CJ, Roulhac PL, Weaver KD, Noto JM, Mietzner TA, et al (2009) Hijacking transferrin bound iron: protein-receptor interactions involved in iron transport in N. gonorrhoeae. Metallomics: integrated biometal science 1: 249–255. 20161024 10.1039/b902860a PMC2749328

[59] Tan S, Noto JM, Romero-Gallo J, Peek RM Jr., Amieva MR (2011) Helicobacter pylori perturbs iron trafficking in the epithelium to grow on the cell surface. PLoS pathogens 7: e1002050 10.1371/journal.ppat.1002050 21589900 PMC3093365

[60] Guttman JA, Samji FN, Li Y, Deng W, Lin A, et al (2007) Aquaporins contribute to diarrhoea caused by attaching and effacing bacterial pathogens. Cellular microbiology 9: 131–141. 10.1111/j.1462-5822.2006.00773.x 16889624

[61] Pedersen GA, Jensen HH, Schelde A-SB, Toft C, Pedersen HN, et al (2017) The basolateral vesicle sorting machinery and basolateral proteins are recruited to the site of enteropathogenic E. coli microcolony growth at the apical membrane. PLoS One 12: e0179122–e0179122. 10.1371/journal.pone.0179122 28636623 PMC5479554

[62] Sun TX, Van Hoek A, Huang Y, Bouley R, McLaughlin M, et al (2002) Aquaporin-2 localization in clathrin-coated pits: inhibition of endocytosis by dominant-negative dynamin. American journal of physiology Renal physiology 282: F998–1011. 10.1152/ajprenal.00257.2001 11997316

[63] Brown D (2003) The ins and outs of aquaporin-2 trafficking. American journal of physiology Renal physiology 284: F893–901. 10.1152/ajprenal.00387.2002 12676734

[64] Nedvetsky PI, Stefan E, Frische S, Santamaria K, Wiesner B, et al (2007) A Role of myosin Vb and Rab11-FIP2 in the aquaporin-2 shuttle. Traffic 8: 110–123. 10.1111/j.1600-0854.2006.00508.x 17156409

[65] Yui N, Lu HA, Chen Y, Nomura N, Bouley R, et al (2013) Basolateral targeting and microtubule-dependent transcytosis of the aquaporin-2 water channel. American journal of physiology Cell physiology 304: C38–48. 10.1152/ajpcell.00109.2012 23015545 PMC3543574

[66] Rai T, Sasaki S, Uchida S (2006) Polarized trafficking of the aquaporin-3 water channel is mediated by an NH2-terminal sorting signal. American journal of physiology Cell physiology 290: C298–304. 10.1152/ajpcell.00356.2005 16135541

[67] Zhu C, Chen Z, Jiang Z (2016) Expression, Distribution and Role of Aquaporin Water Channels in Human and Animal Stomach and Intestines. International journal of molecular sciences 17: 1399. 10.3390/ijms17091399 PMC5037679 27589719

[68] Simonetti B, Cullen PJ (2018) Actin-dependent endosomal receptor recycling. Current opinion in cell biology 56: 22–33. 10.1016/j.ceb.2018.08.006 30227382

[69] Berger CN, Crepin VF, Jepson MA, Arbeloa A, Frankel G (2009) The mechanisms used by enteropathogenic Escherichia coli to control filopodia dynamics. Cell Microbiol 11: 309–322. 10.1111/j.1462-5822.2008.01254.x 19046338 PMC2688667

[70] Campellone KG, Robbins D, Leong JM (2004) EspFU is a translocated EHEC effector that interacts with Tir and N-WASP and promotes Nck-independent actin assembly. Dev Cell 7: 217–228. 10.1016/j.devcel.2004.07.004 15296718

[71] Cantarelli VV, Kodama T, Nijstad N, Abolghait SK, Nada S, et al (2007) Tyrosine phosphorylation controls cortactin binding to two enterohaemorrhagic Escherichia coli effectors: Tir and EspFu/TccP. Cell Microbiol 9: 1782–1795. 10.1111/j.1462-5822.2007.00913.x 17451412

[72] Datsenko KA, Wanner BL (2000) One-step inactivation of chromosomal genes in Escherichia coli K-12 using PCR products. Proc Natl Acad Sci U S A 97: 6640–6645. 10.1073/pnas.120163297 10829079 PMC18686

[73] Gibson DG (2011) Enzymatic assembly of overlapping DNA fragments. Methods Enzymol 498: 349–361. 10.1016/B978-0-12-385120-8.00015-2 21601685 PMC7149801

[74] Zahavi EE, Lieberman JA, Donnenberg MS, Nitzan M, Baruch K, et al (2011) Bundle-forming pilus retraction enhances enteropathogenic Escherichia coli infectivity. Molecular biology of the cell 22: 2436–2447. 10.1091/mbc.E11-01-0001 21613538 PMC3135470

[75] Shen L, Black ED, Witkowski ED, Lencer WI, Guerriero V, et al (2006) Myosin light chain phosphorylation regulates barrier function by remodeling tight junction structure. J Cell Sci 119: 2095–2106. 10.1242/jcs.02915 16638813

[76] Orzech E, Cohen S, Weiss A, Aroeti B (2000) Interactions between the exocytic and endocytic pathways in polarized Madin-Darby canine kidney cells. J Biol Chem 275: 15207–15219. 10.1074/jbc.275.20.15207 10809756

[77] Brown PS, Wang E, Aroeti B, Chapin SJ, Mostov KE, et al (2000) Definition of distinct compartments in polarized Madin-Darby canine kidney (MDCK) cells for membrane-volume sorting, polarized sorting and apical recycling. Traffic 1: 124–140. 11208093 10.1034/j.1600-0854.2000.010205.x

[78] Ramachandran RP, Vences-Catalan F, Wiseman D, Zlotkin-Rivkin E, Shteyer E, et al (2018) EspH Suppresses Erk by Spatial Segregation from CD81 Tetraspanin Microdomains. Infection and immunity 86. 10.1128/IAI.00303-18 PMC6204722 30037792

[79] Wakabayashi Y, Chua J, Larkin JM, Lippincott-Schwartz J, Arias IM (2007) Four-dimensional imaging of filter-grown polarized epithelial cells. Histochem Cell Biol 127: 463–472. 10.1007/s00418-007-0274-x 17308935

[80] Belenky MA, Yarom Y, Pickard GE (2008) Heterogeneous expression of gamma-aminobutyric acid and gamma-aminobutyric acid-associated receptors and transporters in the rat suprachiasmatic nucleus. J Comp Neurol 506: 708–732. 10.1002/cne.21553 18067149

[81] Rappsilber J, Mann M, Ishihama Y (2007) Protocol for micro-purification, enrichment, pre-fractionation and storage of peptides for proteomics using StageTips. Nature protocols 2: 1896–1906. 10.1038/nprot.2007.261 17703201

[82] Cox J, Mann M (2008) MaxQuant enables high peptide identification rates, individualized p.p.b.-range mass accuracies and proteome-wide protein quantification. Nature biotechnology 26: 1367–1372. 10.1038/nbt.1511 19029910

[83] Cox J, Hein MY, Luber CA, Paron I, Nagaraj N, et al (2014) Accurate proteome-wide label-free quantification by delayed normalization and maximal peptide ratio extraction, termed MaxLFQ. Molecular & cellular proteomics: MCP 13: 2513–2526. 10.1074/mcp.M113.031591 24942700 PMC4159666

[84] Shannon P, Markiel A, Ozier O, Baliga NS, Wang JT, et al (2003) Cytoscape: a software environment for integrated models of biomolecular interaction networks. Genome research 13: 2498–2504. 10.1101/gr.1239303 14597658 PMC403769

[85] Szklarczyk D, Morris JH, Cook H, Kuhn M, Wyder S, et al (2017) The STRING database in 2017: quality-controlled protein-protein association networks, made broadly accessible. Nucleic acids research 45: D362–D368. 10.1093/nar/gkw937 27924014 PMC5210637

