# Supplementary figures and images for: Enteropathogenic Escherichia coli remodels host endosomes to promote endocytic turnover and breakdown of surface polarity
Source: PLoS Pathog. 2019 Jun 26;15(6):e1007851. doi: 10.1371/journal.ppat.1007851 (PMC6615643; doi:10.1371/journal.ppat.1007851)

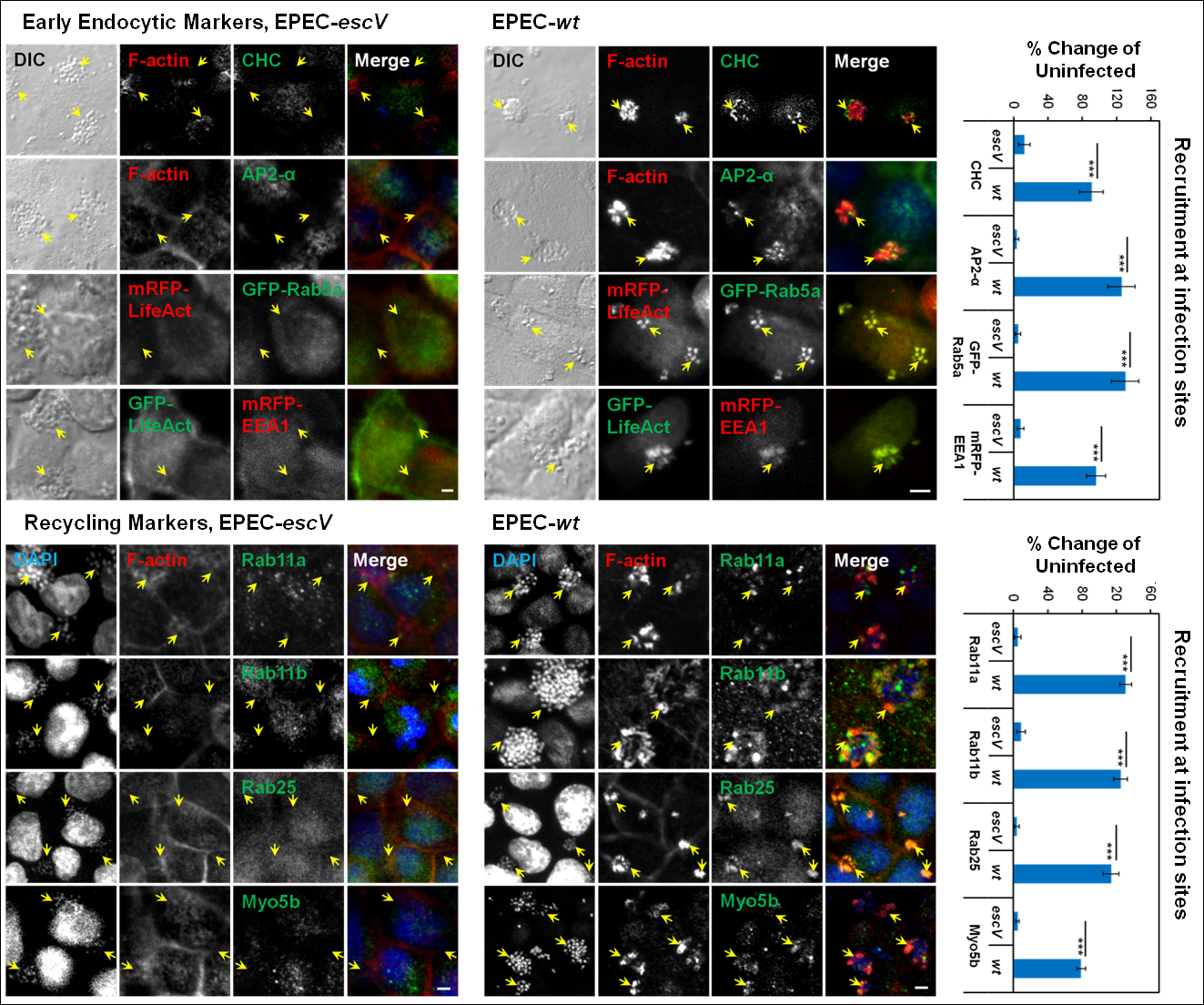

Supplement: S1 Fig — (TIF) [file ppat.1007851.s001.tif]

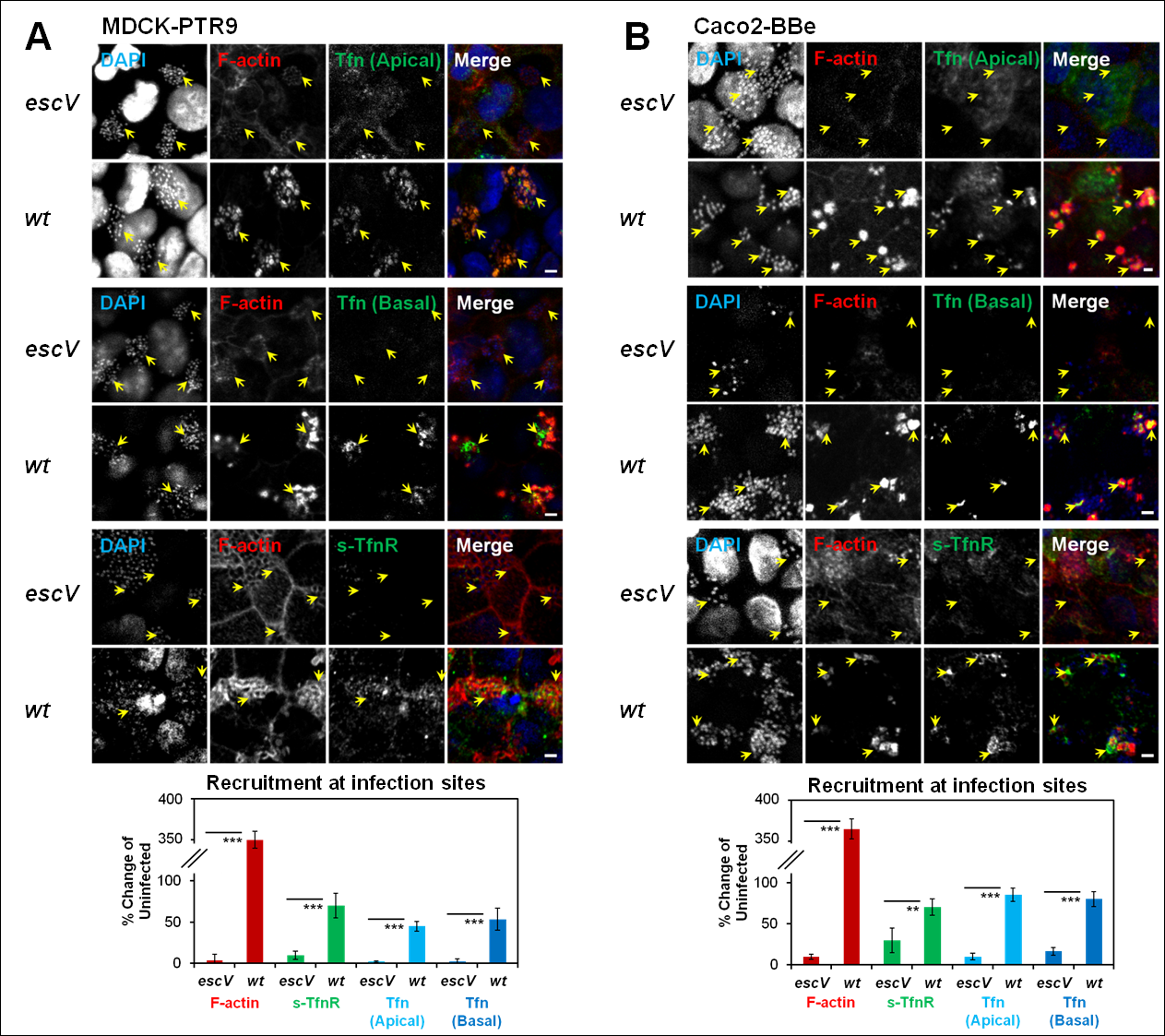

Supplement: S2 Fig — (TIF) [file ppat.1007851.s002.tif]

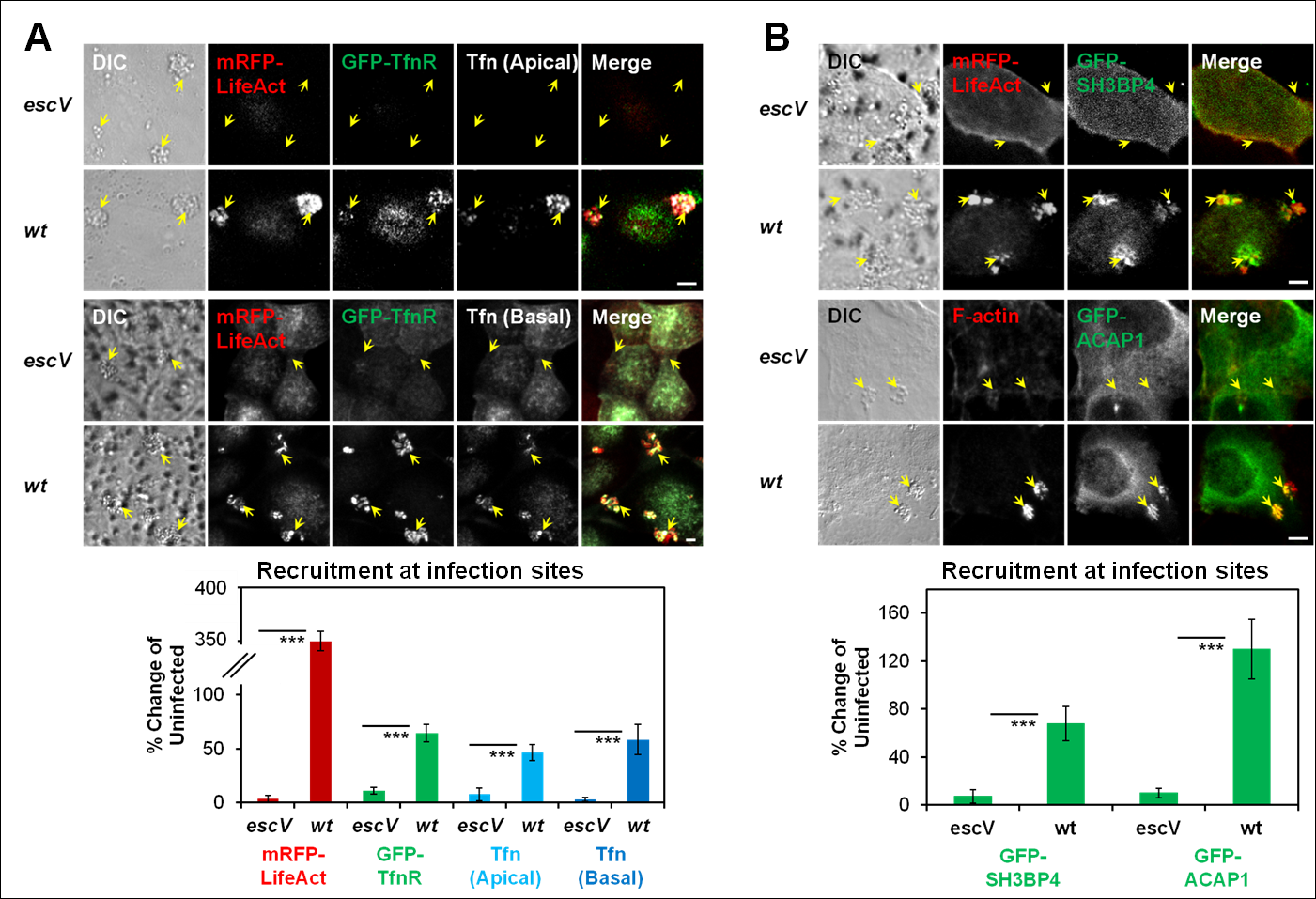

Supplement: S3 Fig — (TIF) [file ppat.1007851.s003.tif]

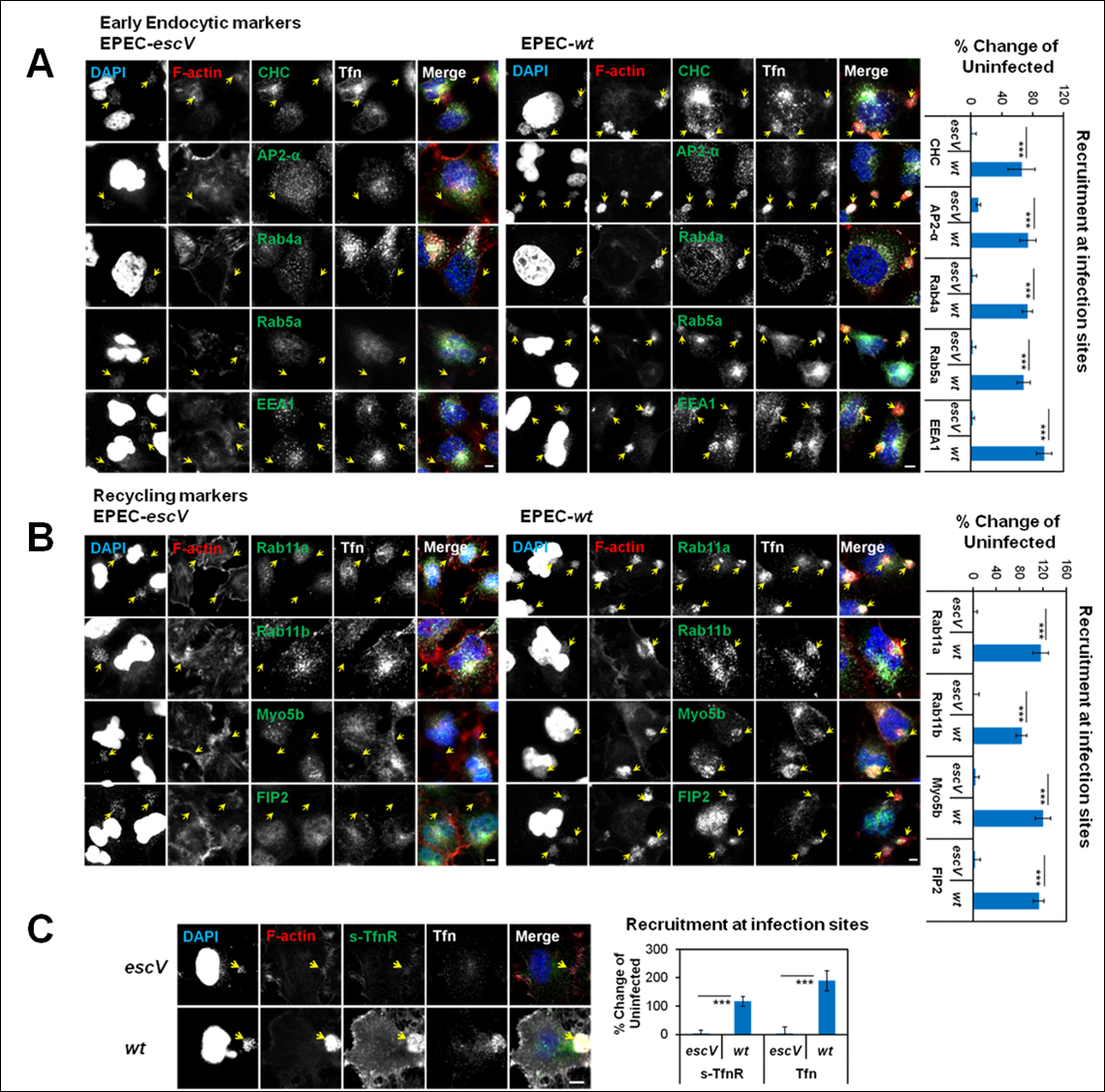

Supplement: S4 Fig — (TIF) [file ppat.1007851.s004.tif]

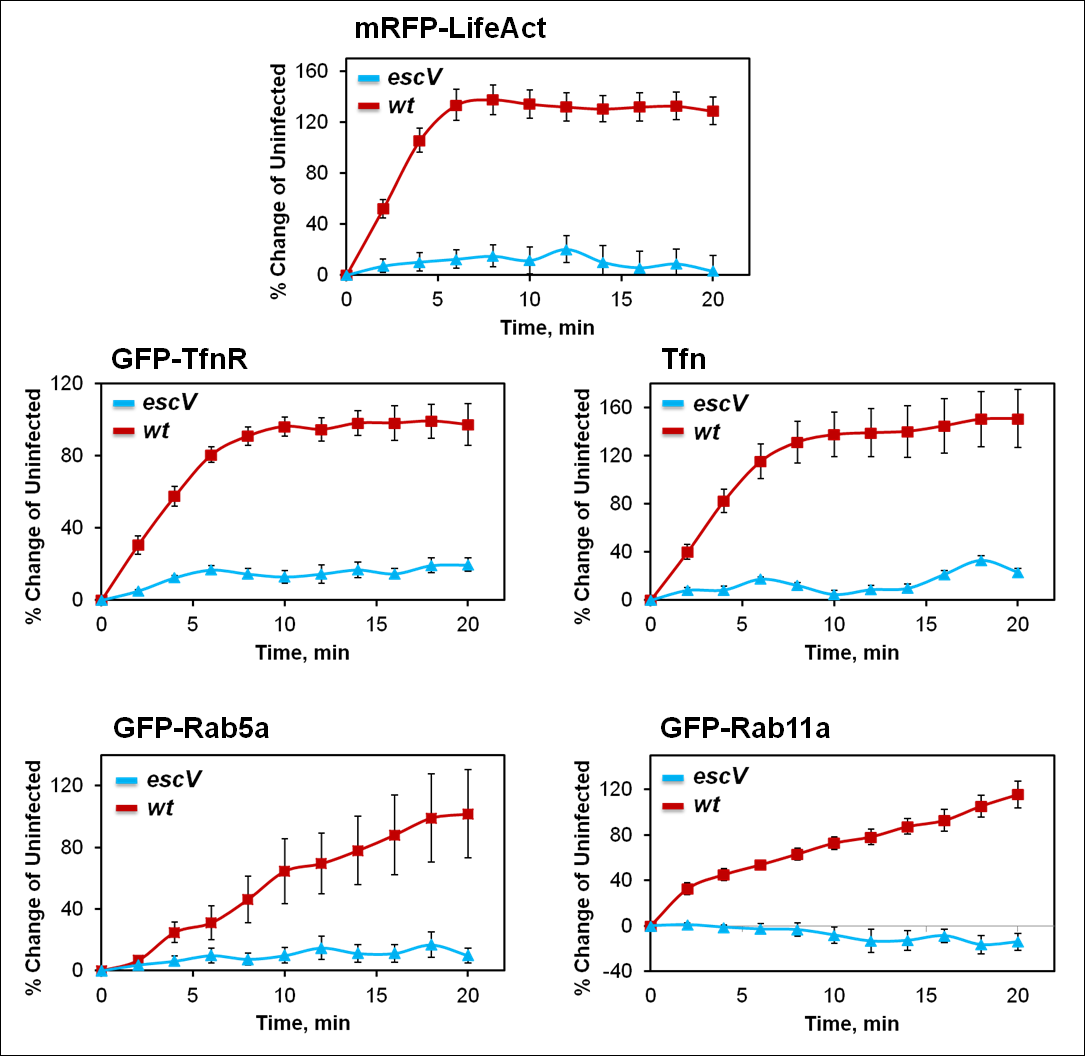

Supplement: S5 Fig — (TIF) [file ppat.1007851.s005.tif]

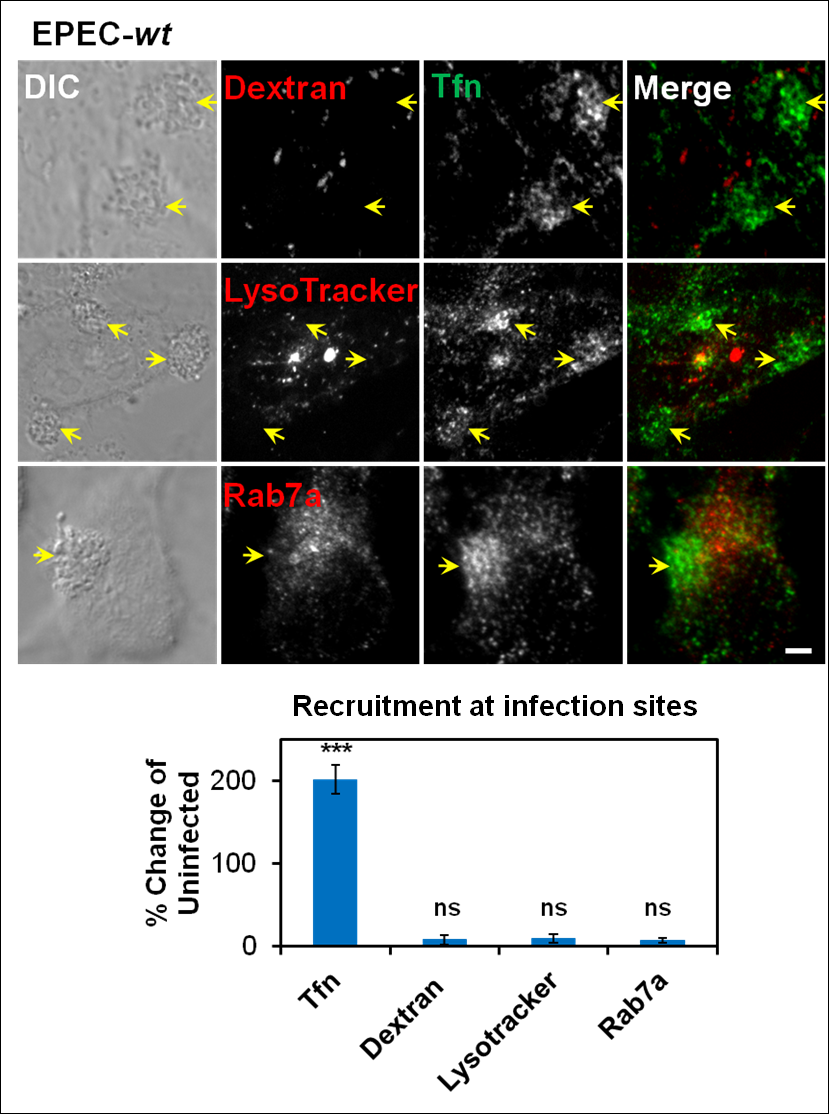

Supplement: S6 Fig — (TIF) [file ppat.1007851.s006.tif]

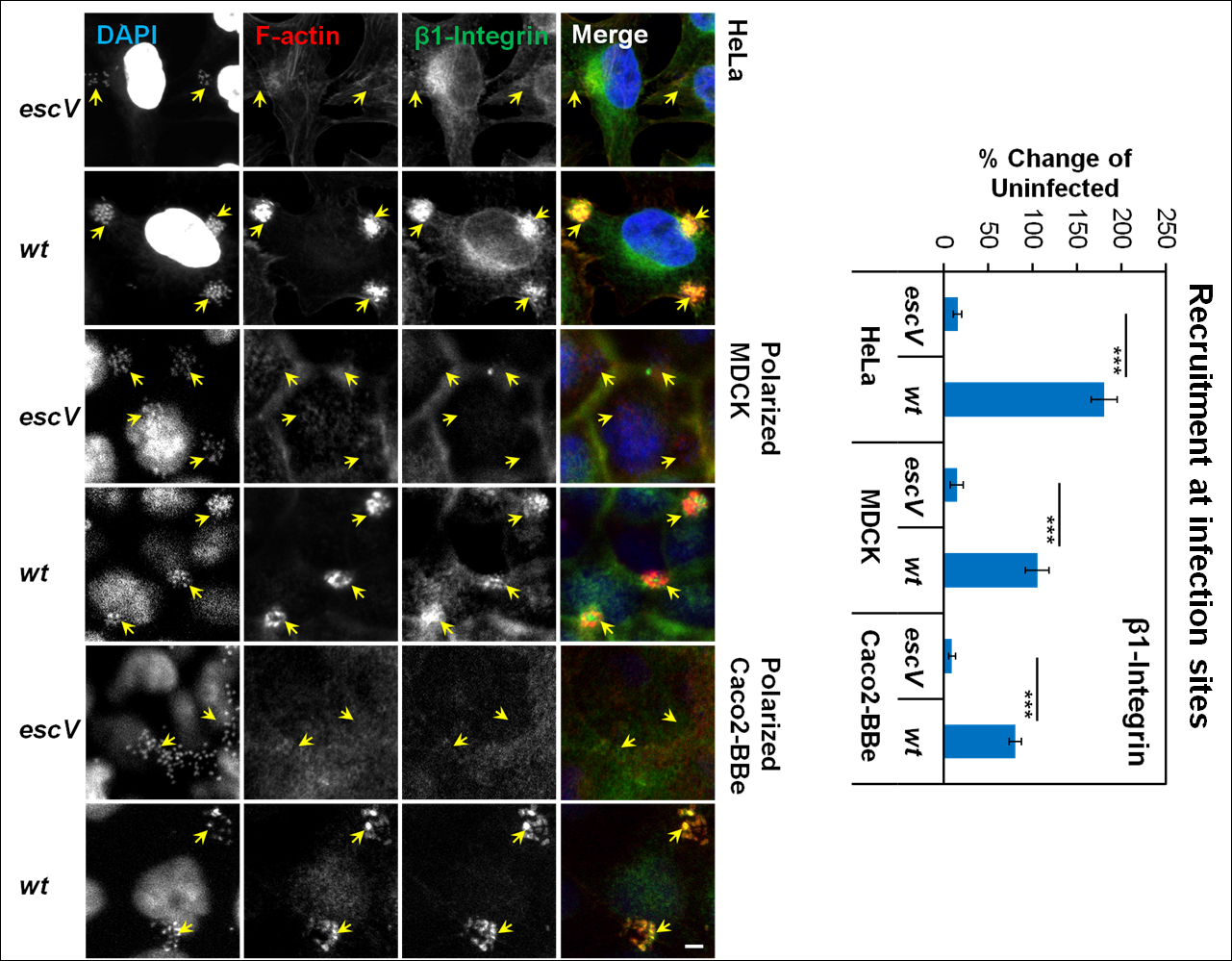

Supplement: S7 Fig — (TIF) [file ppat.1007851.s007.tif]

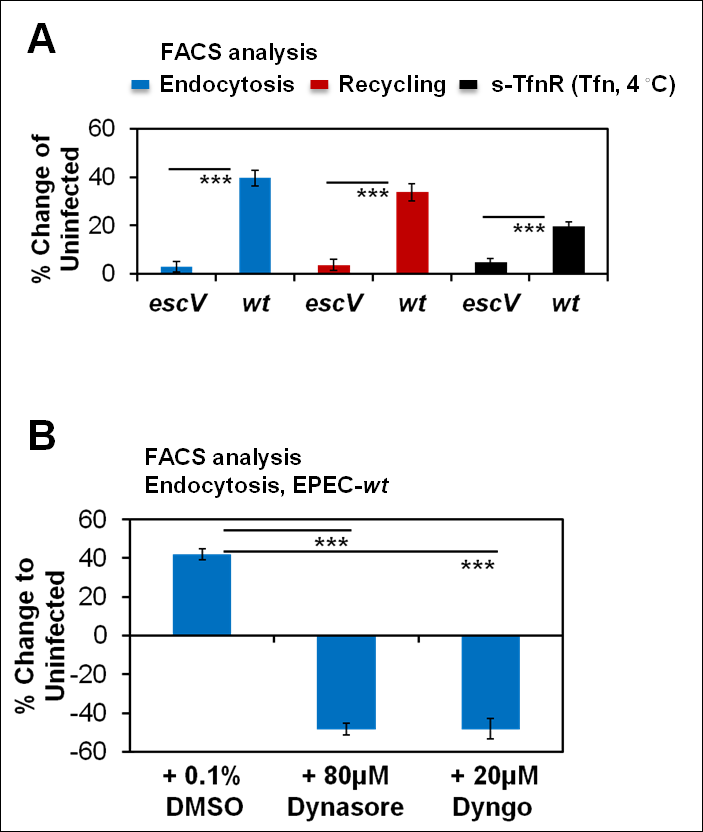

Supplement: S8 Fig — (TIF) [file ppat.1007851.s008.tif]

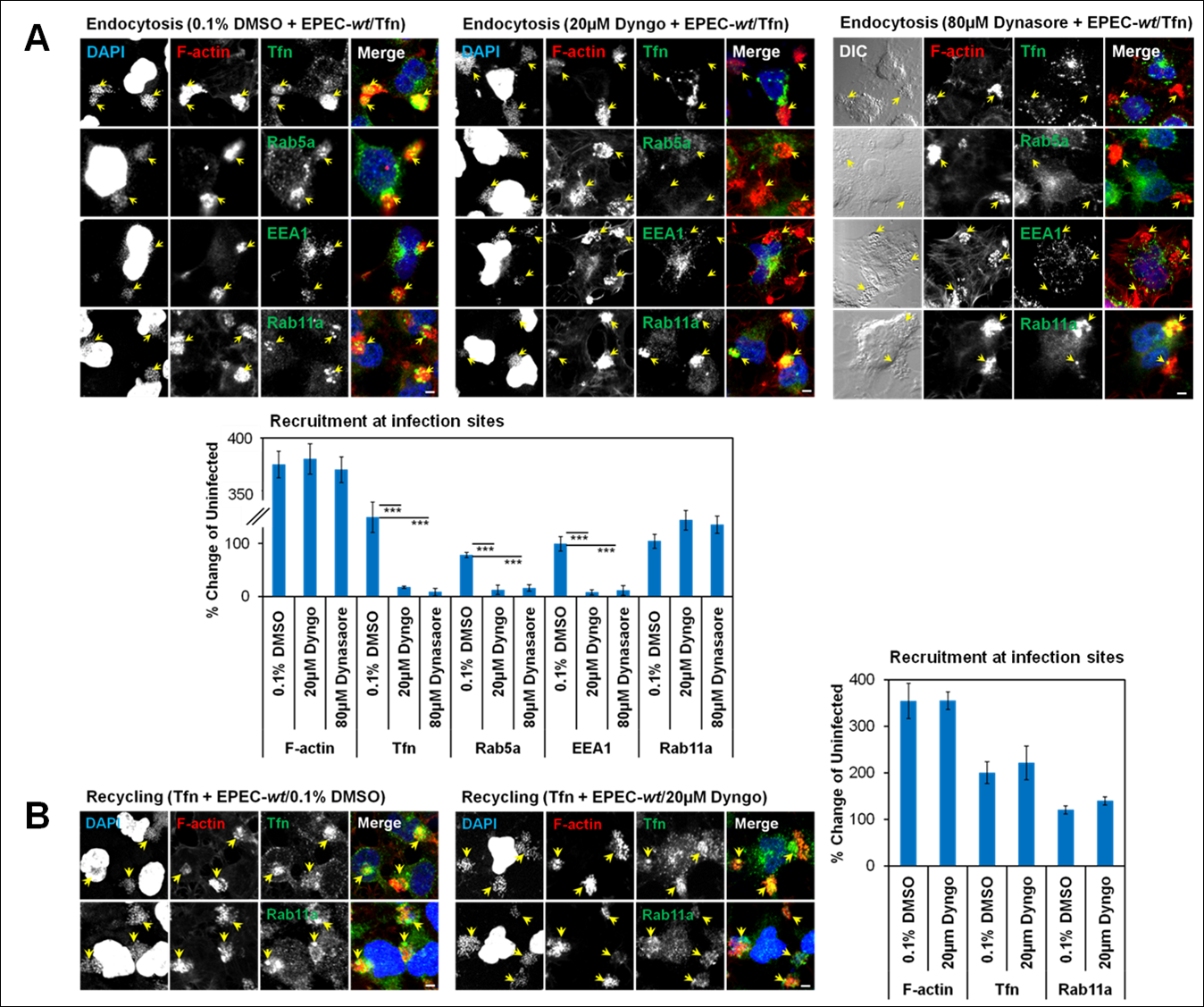

Supplement: S9 Fig — (TIF) [file ppat.1007851.s009.tif]

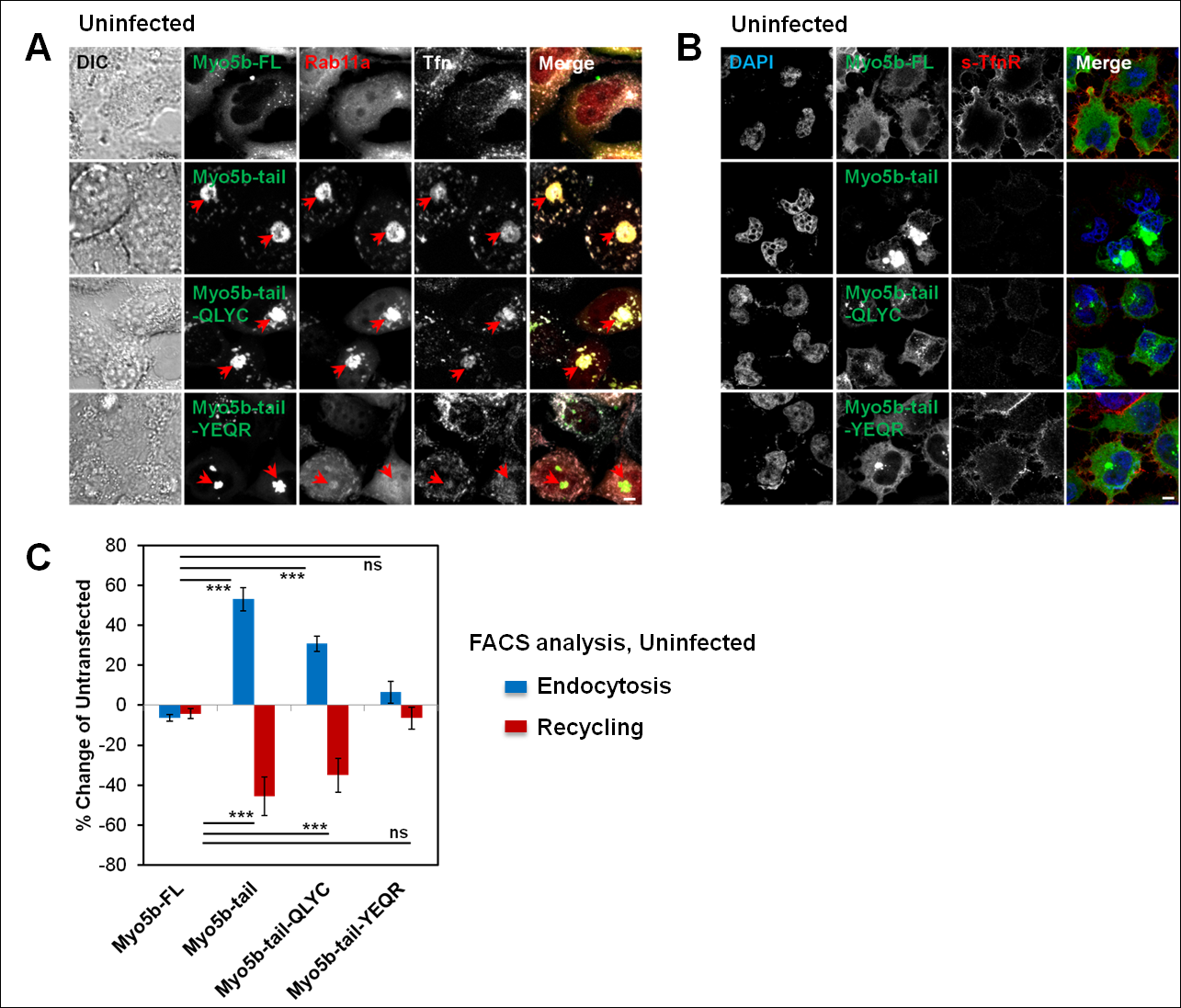

Supplement: S10 Fig — (TIF) [file ppat.1007851.s010.tif]

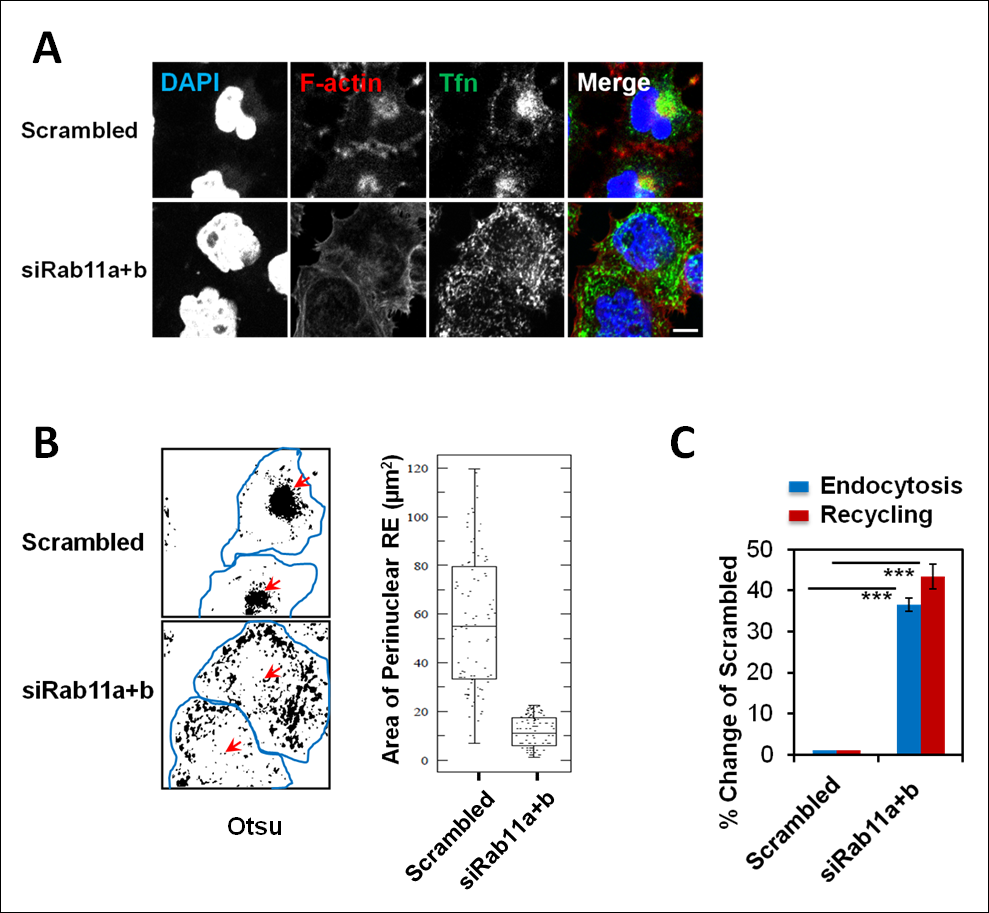

Supplement: S11 Fig — (TIF) [file ppat.1007851.s011.tif]

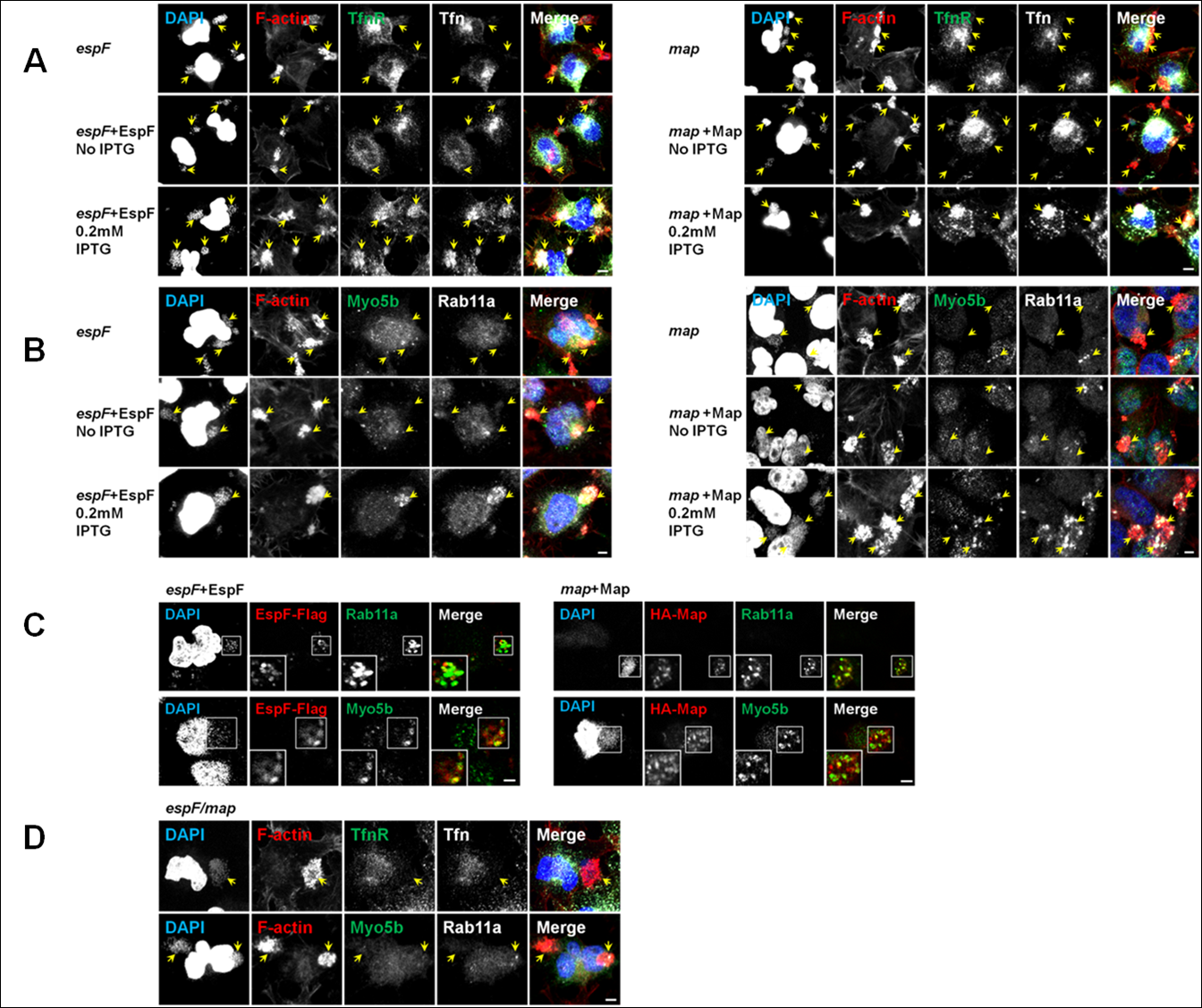

Supplement: S12 Fig — (TIF) [file ppat.1007851.s012.tif]

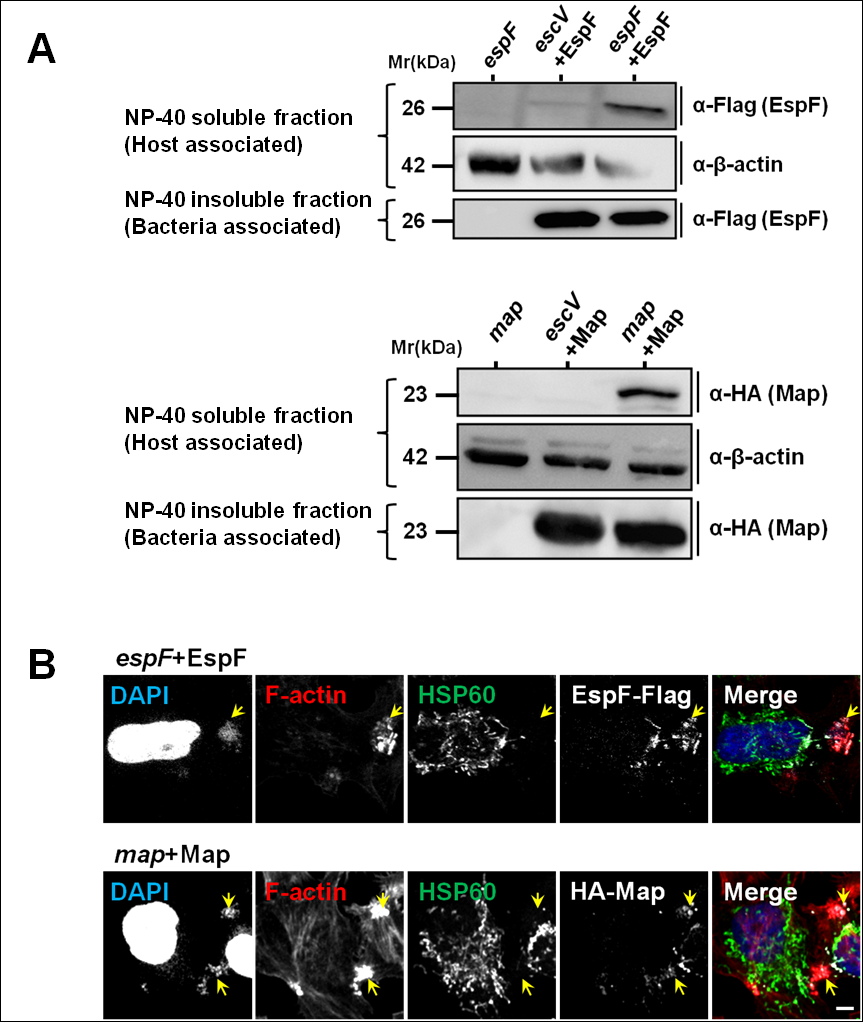

Supplement: S13 Fig — (TIF) [file ppat.1007851.s013.tif]

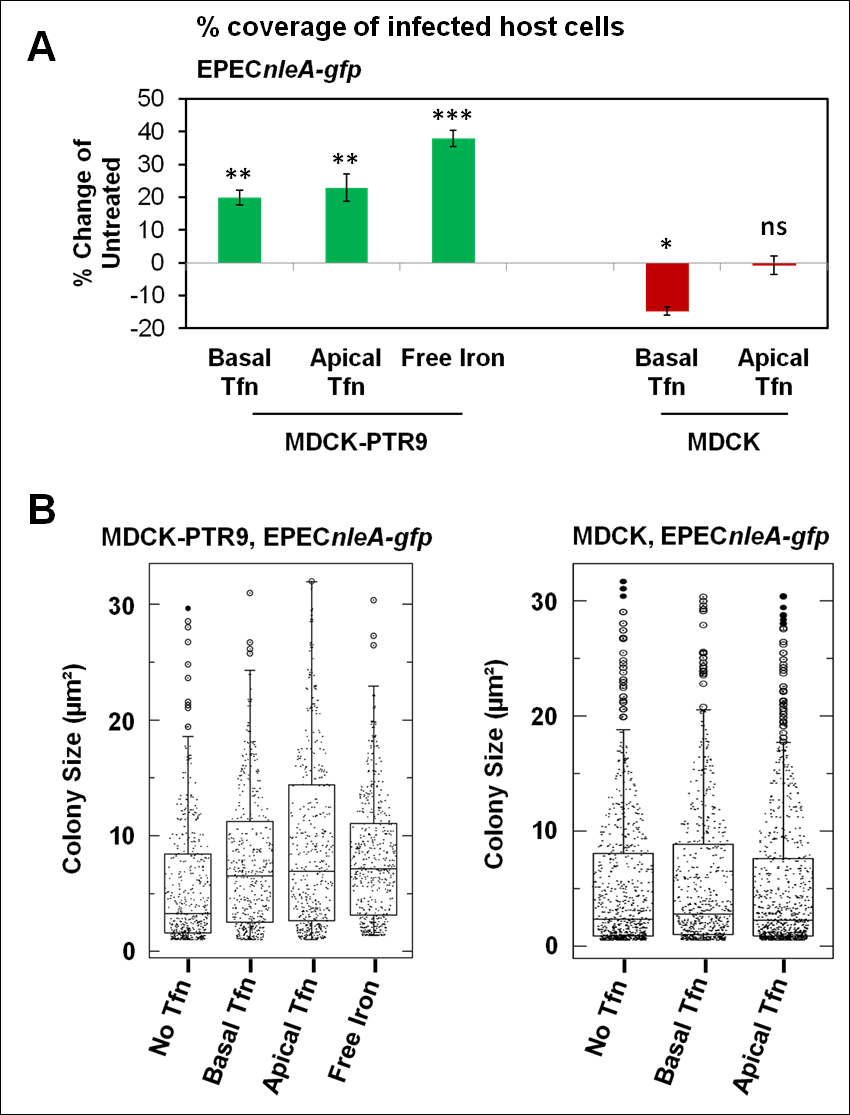

Supplement: S14 Fig — (TIF) [file ppat.1007851.s014.tif]

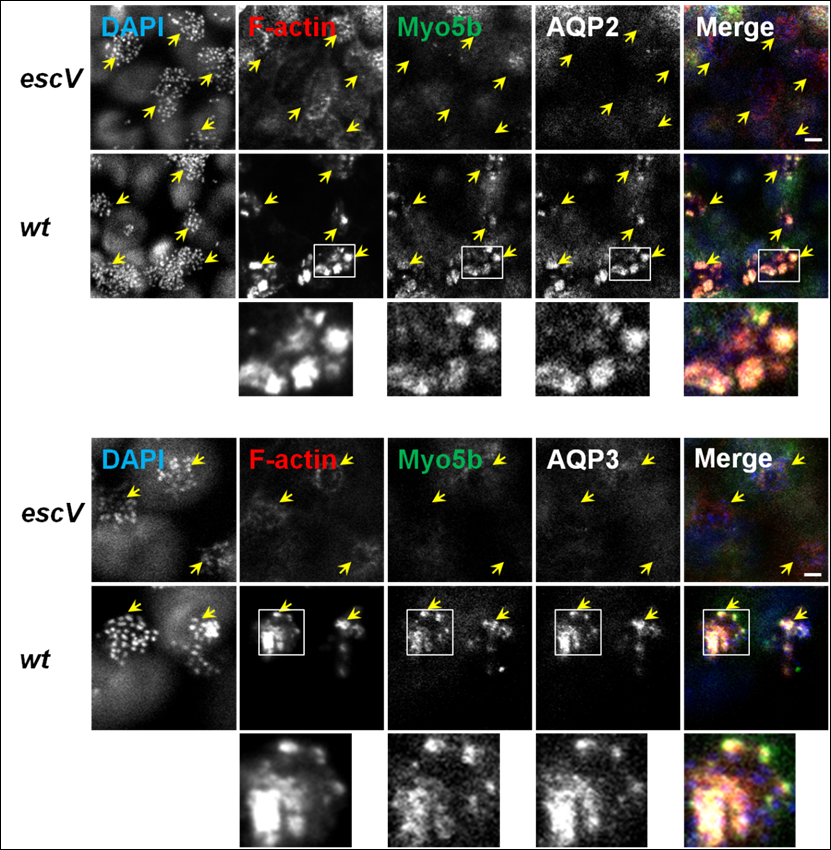

Supplement: S15 Fig — (TIF) [file ppat.1007851.s015.tif]
