# Supplementary material for: Enteropathogenic Escherichia coli remodels host endosomes to promote endocytic turnover and breakdown of surface polarity
Source: PLoS Pathog. 2019 Jun 26;15(6):e1007851. doi: 10.1371/journal.ppat.1007851 (PMC6615643; doi:10.1371/journal.ppat.1007851)
Supplement: S1 Table — (DOCX) [file ppat.1007851.s016.docx]

**S1 Table: EPEC Strains**

| **Strain Name** | **Description** | **Reference and Comments** |
| --- | --- | --- |
| E2348/69 (*wt*) | EPEC isolate, serotype O127:H6 | Wild-type strain; [[3](#_ENREF_3)] |
| SN191 (*escV*) | *escV*::miniTn5*kan* | T3SS deficient mutant; [[4](#_ENREF_4)] |
| ME2018 (*cesT*) | ∆*cesT::Kn* | LEE chaperone; [[5](#_ENREF_5)] |
| ICC202 (*map*) | ∆*map::Kn* | LEE effector; Map, [[6](#_ENREF_6)] |
| XT111 (*espH*) | ∆*espH::Kn* | LEE effector; EspH, [[7](#_ENREF_7)] |
| UMD874 (*espF*) | ∆*espF::Kn* | LEE effector; EspF, [[8](#_ENREF_8)] |
| SE1207 (*espG1/G2*) | ∆*espG1/*∆*espG2* | LEE effector; EspG1/EspG2, [[9](#_ENREF_9)] |
| EM3458 (*tir*) | ∆*tir* | LEE effector; Tir, [[10](#_ENREF_10)] |
| XC2168 (*eae*) | ∆*eae::Kn* | LEE effector; Intimin, [[10](#_ENREF_10)] |
| EM3321 (*3321*) | ∆pp2::*kan*(∆*nleH1, espJ,cif psuedogene::kan*) | Non-LEE pathogenicity island, [[11](#_ENREF_11)] |
| EM3325 (*3325*) | ∆pp4::*kan* (∆*nleG2, nleB, nleC, nleH fragment, nleD::kan*) | Non-LEE pathogenicity island, [[11](#_ENREF_11)] |
| EM3331 (*3331*) | ∆pp6::*kan* (∆*nleH2, nleA.espI, nleF, espO fragment::kan*) | Non-LEE pathogenicity island, [[11](#_ENREF_11)] |
| EM3345 (*3345*) | ∆IE5*::kan* (∆*espG2, espC::kn*) | Non-LEE pathogenicity island, [[11](#_ENREF_11)] |
| EM3347 (*3347*) | ∆IE6*::kan* (∆*lifA, efa1, nleE, nleB, espL::kn*) | Non-LEE pathogenicity island, [[11](#_ENREF_11)] |
| RP8151 (*espF/map*) | E2348/69 Δ*espF*::kan, Δ*map*::cam | This study. |
| EPEC*nleA-gfp* (EM4620) | E2348/69 containing integrated pEM4617 (p*nleA-gfp*) | Non-LEE effector; [[2](#_ENREF_2)] |
