# Supplementary material for: Enteropathogenic Escherichia coli remodels host endosomes to promote endocytic turnover and breakdown of surface polarity
Source: PLoS Pathog. 2019 Jun 26;15(6):e1007851. doi: 10.1371/journal.ppat.1007851 (PMC6615643; doi:10.1371/journal.ppat.1007851)
Supplement: S2 Table — (DOCX) [file ppat.1007851.s017.docx]

**S2 Table: Reagents**

| **Reagent** | **Description** | **Source/Identifies** |
| --- | --- | --- |
| **Tissue Culture Reagents** | | |
| MEM | Minimum Essential Medium (MEM), with Earle’s Salts, Phenol Red and Sodium Bicarbonate but without L-Glutamine | Biological Industries, Beit Ha’emek, Israel. #01-025-1A |
| DMEM | Dulbecco's Modified Eagle Medium (DMEM), with no L-Glutamine, no Sodium Pyruvate | Biological Industries, Beit Ha’emek, Israel. #01-055-1A |
| DMEM without Phenol Red | DMEM with no L-Glutamine, no Sodium Pyruvate and no Phenol Red | Biological Industries, Beit Ha’emek, Israel. #01-053-1A |
| FBS | European Grade Fetal Bovine Serum (FBS) sourced in South America | Biological Industries, Beit Ha’emek, Israel. #04-007-1A |
| BSA | Bovine Serum Albumin (BSA) Solution (10%) | Biological Industries, Beit Ha’emek, Israel. #03-010-1B |
| Trypsin A | Trypsin EDTA Solution A | Biological Industries, Beit Ha’emek, Israel. #03-050-1A |
| Trypsin C | Trypsin EDTA Solution C | Biological Industries, Beit Ha’emek, Israel. #03-053-1A |
| DPBS | Dulbecco's Phosphate Buffered Saline (DPBS), no calcium, no magnesium | Biological Industries, Beit Ha’emek, Israel. #02-023-1A |
| Geneticin (G418 Sulfate) | Selective Antibiotic - Binds to the ribosome and inhibits protein synthesis | Gibco, Life Technologies, Paisley, UK. #11811-031 |
| Lipofectamin 2000 | Transfection Reagent | ThermoFisher Scientific, CA. #11668019 |
| TransIT-X2 6000 | Transfection Reagent | Mirus, Madison, WI. #Mir 6000, |
| **siRNA** | | |
| Scramble siRNA | siGENOME Non-Targeting siRNA Pool #2 | Dharmacon.  #D-001206-14-05 |
| si-Rab11a | siGENOME siRNA reagent targeting the human Rab11a | Dharmacon.  #M-004726-02-0005 |
| si-Rab11b | siGENOME siRNA reagent targeting the human Rab11b | Dharmacon.  #M-004727-02-0005 |
| **Fluorescent Reagents** | | |
| Tfn-AF488 | AlexaFluor (AF)-488 tagged human Transferrin | Jackson Immunoresearch Labs, West Grove, PA. #009-540-050 |
| Tfn-AF647 | AlexaFluor (AF)-647 tagged human Transferrin | Jackson Immunoresearch Labs, West Grove, PA. #009-600-050 |
| Tfn-DL649 | DyLight (DL)-649 tagged human Transferrin | Jackson Immunoresearch Labs, West Grove, PA. #009-490-050 |
| Propidium Iodide (PI) | Cell viability marker | Sigma Aldrich, St. Louis, MO. #P4170 |
| Texas-Red (TR)-Dextran | 70 kDa, Texas Red tagged Dextran | ThermoFisher Scientific. #D-1864 |
| Texas-Red (TR)- phalloidin | Texas-Red-tagged filamentous actin labeling reagent | Invitrogen, Eugene, Oregon. #T7451 |
| DAPI | 4’,6-Diamidine-2’-phenylindole dihydrochloride, DNA labeling reagent | Sigma Aldrich,St. Louis, MO. #D9542 |
| LysoTracker | LysoTracker™ Deep Red | ThermoFisher Scientific. #L12492 |
| **Other Reagents** | | |
| Holo-Tfn | Iron (Fe) Loaded human Transferrin | Biological Industries, Beit Ha’emek, Israel. #41-952-100 |
| Tfn-HRP | Horseradish Peroxidase (HRP)-tagged human Transferrin | Jackson Immunoresearch Labs, West Grove, PA. #009-030-050 |
| IPTG | Isopropyl β-D-1-thiogalactopyranoside | Promega, Madison, WI. #V395D |
| Deferroxamine mesylate | Iron chelator | Sigma Aldrich, St. Louis, MO. #D9533 |
| Triton X-100 | alkylaryl polyether alcohol  Non-ionic detergent | J.T. Baker, Phillipsburg, NJ. #X198-07 |
| MES | 2-(N-morpholino)ethanesulfonic acid | Sigma Aldrich, Steinheim, Germany. #M8250 |
| Protease inhibitor cocktail | Mammalian protease inhibitor cocktail. | Sigma Aldrich, St. Louis, MO. #P8340 |
| Phosphatase inhibitor cocktail | Mammalian phosphatase inhibitor cocktail. | Sigma Aldrich, St. Louis, MO. #P2850 |
| NaVO_4_ | Sodium orthovanadate, phosphatase inhibitor | Sigma Aldrich, St. Louis MO. #S6508 |
| Dynasore | Dynasore hydrate; noncompetitive dynamin 1 and dynamin 2 GTPase activity inhibitor | Sigma Aldrich, St. Louis MO. #D7693 [[12](#_ENREF_12)] |
| Dyngo | Dyngo 4a; a highly potent dynamin inhibitor and Dynasore analog | abcam. #ab120689 [[13](#_ENREF_13)] |
