# Supplementary material for: Enteropathogenic Escherichia coli remodels host endosomes to promote endocytic turnover and breakdown of surface polarity
Source: PLoS Pathog. 2019 Jun 26;15(6):e1007851. doi: 10.1371/journal.ppat.1007851 (PMC6615643; doi:10.1371/journal.ppat.1007851)
Supplement: S3 Table — (DOCX) [file ppat.1007851.s018.docx]

**S3 Table: Expression Constructs**

| **Plasmid name** | **Description** | **Source/ Reference** |
| --- | --- | --- |
| **Bacterial expression constructs** | | |
| pJN61-EspF | A pTrc99A-based vector containing FLAG-tagged espF (EPEC O127:H6 E2348/69) | Michael Donnenberg; [[8](#_ENREF_8)] |
| EspF-mod-wt | pTM007: a pTrc99A-based modular (mod) plasmid that contains two copies of the second proline rich region (PRR) and the native third PRR of EspF | Michael Donnenberg; [[14](#_ENREF_14)] |
| EspF-mod-LA | pKMS13: pTM007 that carries a point mutation on each N-WASP interacting modules (L31A, [[15](#_ENREF_15)]) | Michael Donnenberg;  [[14](#_ENREF_14)] |
| EspF-mod-RD | pKMS12: pTM007 that carries a point mutation on each of the SNX9 interacting modules (R3D, [[16](#_ENREF_16)]) | Michael Donnenberg;  [[14](#_ENREF_14)] |
| pSA10-Map | pSA10 derivative encoding HA-tagged Map (EPEC O127:H6 E2348/69) | Gad Frankel; [[17](#_ENREF_17)] |
| Map-ΔTRL | pSA10-*map*_ΔTRL_: Map whose C-terminal TRL PDZ type I binding motif (aa 608-612) has been deleted | Gad Frankel; [[18](#_ENREF_18)] |
| Map- WA/EA | pSA10-*map*_AxxxA_: Map containing W74A/E78A mutations which disrupt its GTPase binding | Gad Frankel; [[19](#_ENREF_19)] |
| pKD46 | λ RED genes, Amp^r^ | [[20](#_ENREF_20)] |
| pKD3 | Template for the chloramphenicol resistance cassette, Cam^r^ | [[20](#_ENREF_20)] |
| **Mammalian expression constructs** | | |
| mRFP-LifeAct | pCMV/pCAG-LifeAct fused to TagRFP; Labels the actin cytoskeleton | Ibidi. #60102 |
| GFP-LifeAct | pCMV/pCAG-LifeAct fused to TagGFP2; Labels the actin cytoskeleton | Ibidi. #60101 |
| GFP-Rab5a | GFP fused to human Rab5a | Marino Zerial; [[21](#_ENREF_21)] |
| mRFP-EEA1 | TagRFP-T fused to Early Endosome Antigen 1 (EEA1) | Addgene. #42635 |
| Human GFP-TfnR | GFP fused to the N-terminus of the human transferrin receptor | Enrique Rodriguez-Boulan; [[22](#_ENREF_22)] |
| GFP-SH3BP4 | eGFP fused to SH3 binding protein 4 (SH3BP4), also called TTP, which directly binds the TfnR and regulates its CME | Pier Paolo Di Fiore; [[23](#_ENREF_23)] |
| GFP-ACAP1 | eGFP fused to Arf6 GTPase-activating proteins 1 (ACAP1) shown to interact with the TfnR and promote its transport from recycling endosomes | Victor W. Hsu; [[24](#_ENREF_24)] |
| GFP-Rab11a | GFP fused to human Rab11a | James Goldenring; [[25](#_ENREF_25)] |
| mCherry- Rab11a | mCherry fused to human Rab11a | James Goldenring; [[26](#_ENREF_26)] |
| tdEos-Rab11a-7 | tdEos fused to human Rab11a-7 | Addgene. #57664 |
| Myo5b-FL | GFP fused to human Myosin 5b full length (FL) | James Goldenring; [[27](#_ENREF_27)] |
| Myo5b-tail | GFP fused to human Myosin 5b with C-terminal deletion (tail) | James Goldenring; [[27](#_ENREF_27)] |
| Myo5b-tail-QLYC | GFP fused to human Myosin 5b with C-terminal deletion (tail) bearing two point mutations (QLYC) | James Goldenring; [[28](#_ENREF_28)] |
| Myo5b-tail-YEQR | GFP fused to human Myosin 5b with C-terminal deletion (tail) bearing two point mutations (YEQR) | James Goldenring; [[28](#_ENREF_28)] |
