# Supplementary material for: Enteropathogenic Escherichia coli remodels host endosomes to promote endocytic turnover and breakdown of surface polarity
Source: PLoS Pathog. 2019 Jun 26;15(6):e1007851. doi: 10.1371/journal.ppat.1007851 (PMC6615643; doi:10.1371/journal.ppat.1007851)
Supplement: S4 Table — (DOCX) [file ppat.1007851.s019.docx]

**S4 Table: Antibodies**

| **Name** | **Description/Identifier** | **Dilution** |
| --- | --- | --- |
| **Primary antibodies** | | |
| Rabbit anti-CHC | Rabbit polyclonal anti-clathrin heavy chain; abcam. #ab21679 | IF; 1: 1000 |
| Mouse anti-AP2-α | Supernatant produced from the AP6 hybridoma; Detects assembly polypeptide 2 α (AP2-α); [[29](#_ENREF_29)] | IF; 1:200 |
| Mouse anti-Rab5a | Monoclonal anti-Rab5a;  BD Transduction Laboratories, #610724 | IF; 1:100 |
| Mouse anti-EEA1 | Monoclonal anti-EEA1;  BD Transduction Laboratories, #610456 | IF; 1:100 |
| Mouse anti-TfnR (H68.4) | Monoclonal antibody reacts with the N-terminal region of the human transferrin receptor; [[30](#_ENREF_30)] | IF; 1:2500 |
| Mouse anti-TfnR ectodomain (B3/25) | Mouse monoclonal directed against the human receptor ectodomain, Santa Cruz Biotechnology, #sc-65877. [[31](#_ENREF_31)] | IF; 1:50 |
| Rabbit anti-Rab11a | VU57; James Goldenring. [[32](#_ENREF_32)] | IF; 1:200, WB; 1:500 |
| Chicken anti-Myo5b | Prof. James Goldenring. [[33](#_ENREF_33), [34](#_ENREF_34)] | IF; 1:300 |
| Rabbit anti-FIP2 | Polyclonal anti Rab11-FIP2; Sigma Aldrich, #HPA037726 | IF; 1:200 |
| Rabbit anti-Rab25 | Polyclonal anti RAB25; Sigma Aldrich, #HPA010872 | IF; 1:200 |
| Rabbit anti-Rab11b | Monoclonal anti-RAB11B [EPR12725]; abcam, #ab175925 | IF; 1:100, WB; 1:500 |
| Rabbit anti-Rab7a | Monoclonal anti-Rab7a; abcam, #ab137029 | IF; 1: 100 |
| Rat anti-β1-integrin | Rat monoclonal anti-β1-integrin (AIIB2; [[35](#_ENREF_35)]) | IF; 1:1000 |
| Rabbit anti-Rab4a | Polyclonal anti RAB4a; abcam, #ab13252 | IF; 1:100 |
| Rabbit anti-human Tfn | Polyclonal anti-Transferrin; Dako, #A-0061 | WB; 1:2000 |
| Mouse anti-Flag tag | Monoclonal anti-Flag M2; Sigma Aldrich, #F3165 | IF; 1:500, WB; 1:1000 |
| Mouse anti-HA tag | Mouse monoclonal antibody (clone 12CA5) directed against the 9-amino acid sequence derived from the influenza hamagglutinin (HA) protein. abcam, #ab16918 | IF; 1:500, WB; 1:500 |
| Mouse anti-α-tubulin | Monoclonal anti-Tubulin-α antibody, Clone B512, Sigma-Aldrich, #T6074 | WB; 1:2000 |
| Mouse anti-β-actin | Monoclonal Anti-β-Actin Clone AC-15, Sigma Aldrich, #A5441 | WB; 1:5000 |
| Goat anti-Hsp60 | Polyclonal anti HSP60 (k-19); Santa Cruz Biotechnology, #ScL1722; | IF; 1:200 |
| Rabbit anti-AQP2 | Polyclonal anti aquaporin 2; Alomone labs, #AQP-002 | IF; 1:50 |
| Rabbit anti-AQP3 | Polyclonal anti aquaporin 3; Alomone labs, #AQP-003 | IF; 1:50 |
| Rabbit anti-SNX9 | monoclonal anti SH3PX1/SNX9; abcam, ab181856 | WB: 1:2000 |
| Rabbit anti- SNX18 | Polyclonal anti SNX18; abcam, ab111702 | WB: 1:2000 |
| Rabbit anti- SNX33 | Polyclonal anti SNX33; abcam, ab241201 | WB: 1:400 |
| **Secondary antibody** | | |
| Goat anti-mouse IgG, Alexa Fluor 488 | Alexa Fluor 488- AffiniPure Goat Anti-Mouse IgG; Jackson ImmunoResearch Laboratories, #115-545-062 | IF; 1:300 |
| Donkey anti-mouse IgG, Alexa Fluor 488 | Alexa Fluor 488- AffiniPure Goat Anti-Mouse IgG; Thermo-Fisher Scientific, #A-21202 | IF; 1:300 |
| Donkey anti-mouse IgG, Alexa Fluor 594 | Alexa Fluor-594- AffiniPure Donkey Anti-Mouse IgG; Jackson ImmunoResearch Laboratories, #715-585-151 | IF; 1:500 |
| Donkey anti-mouse IgG, Cy5 | Cy5- AffiniPure Donkey Anti-Mouse IgG; Jackson ImmunoResearch Laboratories, #715-175-151 | IF; 1:250 |
| Goat anti-rabbit IgG, Alexa Fluor 488 | Cross-Adsorbed anti rabbit IgG; Alexa Fluor 488; Thermo-Fisher Scientific, #A-11008 | IF; 1:300 |
| Donkey anti-rabbit IgG, Alexa Fluor 488 | Highly cross-Adsorbed anti rabbit IgG; Alexa Fluor 488; Thermo-Fisher Scientific, #A-21206 | IF; 1:300 |
| Donkey anti-rabbit IgG, Alexa Fluor 647 | Alexa Fluor-647- AffiniPure Donkey Anti-rabbit IgG; Jackson ImmunoResearch Laboratories, #711-602-152 | IF; 1:300 |
| Donkey anti-rat IgG, Cy3 | Cy3- AffiniPure Donkey Anti-rat IgG; Jackson ImmunoResearch Laboratories, #712-165-153 | IF; 1:250 |
| Donkey anti-rat IgG, Cy5 | Cy5- AffiniPure Donkey Anti-rat IgG; Jackson ImmunoResearch Laboratories, #712-175-153 | IF; 1:250 |
| Donkey anti-goat IgG, Alexa Fluor 488 | Cross-Adsorbed anti goat IgG; Alexa Fluor 488; Thermo-Fisher Scientific, #A-11055 | IF; 1:300 |
| Donkey anti-goat IgG, Alexa Fluor 647 | Cross-Adsorbed anti goat IgG; Alexa Fluor 647; Thermo-Fisher Scientific, #A-21447 | IF; 1:100 |
| Peroxidase goat anti-mouse IgG | Jackson ImmunoResearch Laboratories, #115-035-166 | WB; 1:10000 |
| Peroxidase goat anti- rabbit IgG | Jackson ImmunoResearch Laboratories, #111-035-003 | WB; 1:10000 |
