# Supplementary material for: Enteropathogenic Escherichia coli remodels host endosomes to promote endocytic turnover and breakdown of surface polarity
Source: PLoS Pathog. 2019 Jun 26;15(6):e1007851. doi: 10.1371/journal.ppat.1007851 (PMC6615643; doi:10.1371/journal.ppat.1007851)
Supplement: S5 Table — (DOCX) [file ppat.1007851.s020.docx]

**S5 Table: Primers**

| **Numbers** | **Sequences (5’-3’)** | **Usages** |
| --- | --- | --- |
| 1354 | GCAATGGTAGGTAGAGCGTTAGCTCAGGCGGTTACACAAACTCTTAGACCCGTGTAGGCTGGAGCTGCTTC | Creation of RP8151 |
| 1355 | CTGCAATCGCCTTCTCAGTTAGAGCCTTGATATCACTGATTTTCGCGGTGCCATATGAATATCCTCCTTAG | Creation of RP8151 |
| 1371 | GTATCCACTCATGACCATCG | Creation of RP8151 |
| 1495 | CTCTGGAATCGACAGAATCAGCC | Creation of RP8151 |
| 4197 | GAGCTAACGCTCTACCTACC | Creation of RP8151 |
| 4198 | CTAACTGAGAAGGCGATTGC | Creation of RP8151 |
| 115 | GATCTTCCGTCACAGGTAGG | Verification of RP8151. |
| 1682 | GCTAAACCAGCAGCAATTGCG | Verification of RP8151 |
