# Supplementary material for: Enteropathogenic Escherichia coli remodels host endosomes to promote endocytic turnover and breakdown of surface polarity
Source: PLoS Pathog. 2019 Jun 26;15(6):e1007851. doi: 10.1371/journal.ppat.1007851 (PMC6615643; doi:10.1371/journal.ppat.1007851)
Supplement: S1 Text — (DOCX) [file ppat.1007851.s030.docx]

# Supporting Information

# Supporting Methods

**Time-lapse confocal live imaging and analysis of protein recruitment to infection sites:** HeLa cells were seeded on ibiTreat μ-slide 8 well plates and transfected with plasmids encoding GFP-tagged or mRFP-tagged proteins (**S3** **Table**), as described before. Cells were washed with warm PBS and exposed to a 1:1:1 (100 μl each) mixture of DMEM, activated EPEC and Tfn-DL649 (5μg/ml, **S2 Table**). Cells were then scanned with an Olympus Fv-1200 confocal microscope equipped with a 60x /1.42 oil immersion objective and an environmental chamber (37^o^C, 5% CO_2_; Life Imaging Services, Basel, Switzerland). Confocal Z-stacks consisting of 5-8 optical sections at 1 μm spacing which covered the entire cell volume were acquired every 2 minutes, for up to 60 min. This has produced a XYZT 4-dimensional image stack for each experiment. Image analysis was done using FIJI (NIH). A mean intensity Z-projection was generated for each XYZT stack, thus producing an XYT stack. The limiting dimensions of cell-associated EPEC microcolonies were manually defined as the region of interest (ROI), using the ‘polygon selections’ tool in XYT, and termed ‘infection site’. For the fluorescent proteins, the average fluorescence (average pixel intensity-PI) in an infection site at time t (PI_t_) was normalized to the average PI at time zero (PI_0_; i.e. the first time frame an EPEC microcolony was detected to adhere to the host cell). A fluorescence accumulation factor (FAF) at a given infection time (t) was defined as follows:

$${FAF}_{t} =\frac{{PI}_{t}-{PI}_{0}}{{PI}_{0}}$$

The mean value of FAF_t_ for all microcolonies (a=1,2…N) was defined as follows:

$$MeanFAF_{t}= \frac{\sum_{a=1}^{N} FAF_{t,a}}{N}$$

Standard error for each time point was defined as follows:

$$\frac{\sqrt{\sum_{a=1}^{N} (FAF_{t,a}-meanFA{F_{t})}^{2}}}{N-1}$$

In all cases the calculated $MeanFAF_{t}$ is presented as percent change of the site before microcolony attachment and termed “% Change of Uninfected Cells”.

**Effector translocation assay:** The assay was performed essentially as described [[1](#_ENREF_1)]. Briefly, HeLa cells cultured on a 6-well plate were infected for 30 minutes at 37°C with EPEC*-espF*, EPEC*-espF* +EspF or EPEC*-escV*+EspF. For analyzing Map translocation, cells were infected with EPEC*-map*, EPEC*-map* +Map, or with EPEC*-escV*+Map. Cell lysates were analyzed by Western blotting probed with anti-Flag (for detecting EspF) or anti-HA (for detecting Map) antibodies (**S4 Table**).

**Determining host cell colonization by infecting host cells with bacteria expressing contact-induced NleA-GFP:** Polarized monolayers of MDCK, or MDCK-PTR9 cells were infected with EPEC*nleA-gfp* [[2](#_ENREF_2)]. Notably, the production of NleA-GFP by this EPEC strain is induced only upon bacterial attachment to the host cell by a post-transcriptional process. Therefore, within a given attached microcolony of EPEC*nleA-gfp*, only bacteria that are directly attached to the host cell will produce NleA-GFP, and appear as a layer of green labeled bacteria [[2](#_ENREF_2)]. Cells were initially incubated for 12 hrs (37 °C; 5% CO_2_) in plain MEM (Biological Industries), whose normal composition is not supplemented with iron. These cells were infected for three hours at 37 °C in plain MEM, or in MEM supplemented with Tfn-AF647 (20 µg/ml) introduced to their basal or apical compartments. In another set of experiments, cells were infected with plain MEM supplemented with 0.1mg/L Fe(NO_3_)_3_·9H_2_O (“free iron”). Cells were then fixed and imaged by confocal microscopy. The maximum fluorescence projection encompassing the entire cell volume (dark-to-dark) was generated. The distribution of fluorescent signals within the imaged field was first identified by the FIJI (NIH) ‘Huang Threshold’ tool. The area occupied by distinct particles (i.e. individual cell-associated NleA-GFP expressing bacteria), as well as the entire area covered by them was measured using the FIJI (NIH) ‘Particle Analysis’ tool. The distribution of bacterial microcolony area values was presented in a box and whisker diagram, using a ‘box plot tool’ (<http://www.physics.csbsju.edu/stats/display.distribution.html>).

# Supporting Figure Captions

S1 Fig. T3SS-dependent recruitment of early and recycling endocytic elements at apical infection sites. Polarized MDCK cells were infected with EPEC-*escV*, or EPEC-*wt* and then immunolabeled with anti-CHC, AP2-α, Rab11a, Rab11b, Rab25 and Myo5b antibodies. Cells were also stained with TR-phalloidin (F-actin) and DAPI (DNA). Visualization of endosomal Rab5a and EEA1 along with F-actin was achieved by cell co-transfection with GFP-Rab5a/mRFP-LifeAct, or with mRFP-EEA1/GFP-LifeAct encoding plasmids, respectively. Cells were imaged by confocal microscopy and analyzed for marker recruitment at infection sites. Representative images and graphs showing the degree of recruitment are shown. Results are mean ± SE; n≥30 bacterial microcolonies (indicated by arrows) analyzed in three independent experiments. Bar = 5 µm.

S2 Fig. T3SS-dependent recruitment of basally or apically internalized Tfn/TfnR to apical infection sites. Polarized MDCK-PTR9 **(A)** or Caco2-BBe **(B)** cells were infected with EPEC-*escV*, or EPEC-*wt*, and Tfn-AF488 (5 µg/ml) was applied at either their apical [Tfn (apical)] or basolateral [Tfn (Basal)] cell surface during infection. Cells were fixed, permeabilized and stained with DAPI and TR-phalloidin. In another experiment, cells were fixed, but not permeabilized, and transferrin receptors on their apical cell surface (s-TfnR) were immunostained with the B3/25 antibody, recognizing the ectodomain of these receptors (see **S4 Table**). Cells were fixed, permeabilized, stained with TR-phalloidin, imaged by confocal microscopy and analyzed for Tfn and s-TfnR recruitment at infection sites. Results are mean ± SE; n≥30 bacterial microcolonies analyzed in three independent experiments. Arrows point towards infection sites. Bar = 5 µm.

S3 Fig. T3SS-dependent recruitment of Tfn, TfnR, SH3BP4 and ACAP1 in polarized MDCK cells. (A) T3SS-dependent recruitment of basolaterally or apically internalized Tfn at apical infection sites in living cells. MDCK-GFP-TfnR cells were transfected with mRFP-LifeAct (F-actin) encoding plasmid and cultured on translucent Transwells®. Cells were then plated in the underside of a Transwell filter. The apical surface of these cells was infected with EPEC-*escV* or EPEC-*wt* and exposed to basolateral Tfn-DL649 [Tfn(basal)] for the last 60 min of infection. Alternatively, Tfn-DL649 was internalized from the apical surface of the cells [Tfn(apical)]. Cells were analyzed by confocal live cell imaging. Representative images and the degree of marker clustering at infection sites are shown. Results are mean ± SE; n≥30 bacterial microcolonies were analyzed in three independent experiments. Arrows point towards infecting microcolonies. Bar = 5 µm. (B) T3SS-dependent recruitment of Tfn specific endocytic (SH3BP4) and recycling (ACAP1) regulators at apical infection sites of living cells. MDCK cells were co-transfected with GFP-SH3BP4 and mRFP-LifeAct encoding plasmids and cultured on translucent Transwells®, as above. The apical surface of these cells was infected with EPEC-*escV* or EPEC-*wt* and analyzed by confocal live cell imaging. In another experiment, MDCK cells were transfected with GFP-ACAP1 encoding plasmids and cultured on Transwell filters. The apical surface of these cells was infected with EPEC-*escV* or EPEC-*wt.* cells were then fixed, stained with TR-Phalloidin and analyzed by confocal imaging. Representative images and the degree of marker clustering at infection sites are shown. Results are mean ± SE; n≥30 bacterial microcolonies were analyzed in three independent experiments. Arrows point towards infecting microcolonies. Bar = 5 µm.

S4 Fig. Type III secreted elements elicit the recruitment of endocytic markers to infection sites. **(A-B) T3SS-dependent recruitment of early endocytic (A) and recycling (B) markers to bacterial infection sites.** HeLa cells were infected with EPEC-*escV*, or EPEC-*wt*, concomitant to Tfn-AF647 exposure. Cells were then immunolabeled with antibodies directed against the indicated markers, stained with TR-phalloidin (F-actin) and DAPI and imaged by confocal microscopy. Representative images and quantitative analysis of recruitment of these markers at infection sites are shown. Arrows point towards infecting microcolonies. Results are mean ± SE; n≥30 microcolonies were analyzed in three independent experiments. Bar = 5 µm. **(C) T3SS-dependent recruitment of cell surface exposed TfnR (s-TfnR) at bacterial infection sites.** HeLa cells were infected with EPEC- *escV*, or EPEC-*wt*, concomitant to Tfn-AF647exposure. Cells were then fixed and s-TfnR was immunolabeled with B3/25 antibodies. Cells were permeabilized, stained with TR-phalloidin and DAPI and imaged by confocal microscopy. Representative images and quantification of Tfn/s-TfnR recruitment at infection sites are shown. An arrow points towards an infecting microcolony. Results are mean ± SE; n≥50 bacterial microcolonies were analyzed in three independent experiments. Bar = 5 µm.

S5 Fig. Dynamic recruitment of early and recycling endocytic markers at infection sites. HeLa cells expressing mRFP-LifeAct along with GFP-TfnR, GFP-Rab5a, or GFP-Rab11a were exposed to Tfn-DL649, infected with EPEC-*escV* or EPEC-*wt* under the microscope and subjected to time-lapse confocal imaging (**S1-S6 Movies**). The recruitment of the indicated fluorescent markers at infection sites was determined. Results are mean ± SE; n≥30 microcolonies were analyzed in three independent experiments.

S6 Fig. Unlike Tfn, **fluid-phase (Dextran), lysosomal (LysoTracker), and late endosomal (Rab7a) markers are not recruited at EPEC-*wt* infection sites.** HeLa cells were infected with EPEC-*wt* and simultaneously exposed to a mixture of Tfn-AF488/TR-Dextran (70kDa), or Tfn-AF488/Deep-Red LysoTracker. Thirty mins after infection, live cells were imaged by confocal microscopy. In another experiment, HeLa cells were infected with EPEC-*wt* and concomitantly exposed to Tfn-AF488. Cells were then fixed immunolabeled with anti-Rab7a antibodies and analyzed by confocal microscopy. Infecting bacterial microcolonies were visualized by DIC microcopy. Representative images and graphs showing the degree of recruitment at infection sites are presented. Results are mean ± SE; n≥30 bacterial microcolonies (indicated by arrows) analyzed in three independent experiments. Bar = 5 µm.

S7 Fig. **T3SS-dependent recruitment of β1-Integrin at infection sites.** HeLa, polarized MDCK and Caco2-BBe cells were infected with EPEC-*escV* or EPEC-*wt,* fixed, immunostained with anti- β1-integrin antibodies, followed by TR-phalloidin/DAPI staining. Cells were analyzed by confocal microscopy. Representative images and graphs showing the degree of marker recruitment at infection sites are presented. Results are mean ± SE; n≥30 bacterial microcolonies (indicated by arrows) analyzed in three independent experiments. Bar = 5 µm.

S8 Fig. Type III secreted elements elicit increased endocytic turnover in non-polar cells. **(A) EPEC-*wt* stimulates TfnR endocytic turnover.** HeLa cells were infected with EPEC-*escV*, *wt*, or left uninfected. Tfn-AF647 endocytosis, recycling and surface bound ligand were determined by FACS analysis. Results are mean ± SE; n≥6 measurements were analyzed in three independent experiments. **(B) Dyngo and Dynasore inhibit the EPEC-*wt* promoted increase in Tfn endocytosis.** HeLa cells were pretreated with 0.1% DMSO, or with Dyngo or Dynasore. Cells were then infected with EPEC-*wt*, or left uninfected, in the presence of Tfn-AF647 and the dynamin inhibitors, or DMSO. Tfn endocytosis was assessed by FACS. Results are mean ± SE; n≥6 measurements were analyzed in three independent experiments.

S9 Fig. Endocytic activity is not essential for the recruitment of Tfn/Rab11a positive recycling endosomes at infection sites. **(A). Dynamin inhibitors diminished the clustering of early, but not of recycling endocytic markers at EPEC-*wt* infection site.** HeLa cells were pre-treated (60 min 37 °C) with 0.1% DMSO, or with Dyngo or Dynasore, and then subjected to EPEC-*wt* infection and Tfn-AF488 exposure in the presence of the inhibitors. Cells were then fixed, immunolabeled with anti-Rab5a, anti-EEA1 or anti-Rab11a antibodies, stained with TR-phalloidin and DAPI, and analyzed by confocal microscopy. Representative images and quantitative analysis of the indicated markers recruitment at infection sites are shown. Results are mean ± SE; n≥30 infection sites were analyzed in three independent experiments. Arrows point towards infecting bacterial microcolonies. Bar = 5 µm. **(B) Dynamin inhibition had no effect on the clustering of Rab11a and pre-internalized Tfn.** Tfn-AF488 was first endocytosed into HeLa cells. Surface-bound Tfn was stripped-off and cells were infected with EPEC-*wt* under continuous presence of DMSO, or Dyngo. Cells were immunolabeled with anti-Rab11a antibodies, stained with TR-phalloidin and DAPI, and imaged by confocal microscopy. Representative images and quantitative analysis of the indicated markers recruitment at infection sites are shown. Results are mean ± SE; n≥30 bacterial microcolonies were analyzed in three independent experiments. Arrows point towards infecting EPEC microcolonies. Bar = 5 µm.

S10 Fig. Myo5b is essential for Rab11-dependent TfnR trafficking to the cell surface. **(A) Expression of GFP-Myo5b mutant deficient in motor activity but can bind Rab11, causes the sequestration of Rab11a and Tfn into large endosomal puncta.** HeLa cells transiently co-expressing mCherry-Rab11a and Myo5b-FL, GFP-Myo5b-tail, GFP-Myo5b-tail-QLYC, or GFP-Myo5b-tail-YEQR were exposed to Tfn-AF647 for 30 min and imaged by live confocal microscopy. Representative images are shown. Red arrows point towards Rab11a and Tfn sequestered into large endosomal puncta. Bar = 5 µm. **(B) Expression of GFP-Myo5b mutant deficient in motor activity but can bind Rab11 diminishes the levels of surface TfnR.** HeLa cells transiently expressing the indicated Myo5b constructs were fixed and surface TfnR were immunolabeled with the B3/25 antibody. Cells were then stained with DAPI and imaged by confocal microscopy. Representative images are shown. Bar = 5 µm. **(C) Sequestering of Rab11a/Tfn positive endosomes inhibits Tfn recycling.** HeLa cells expressing the indicating GFP-Myo5b constructs were exposed to Tfn-AF647 and subjected to endocytosis and recycling analyses in the GFP-Myo5b expressing cells using flow cytometry. Results are mean ± SE; n≥12 were analyzed in three independent experiments.

S11 Fig. Knockdown of Rab11a and Rab11b by siRNA redistributes the Tfn-positive endosomes to the cell periphery and increases Tfn endocytic turnover. **(A) Simultaneous silencing of Rab11a and Rab11b leads to peripheral localization of Tfn-positive endosomes.** HeLa cells were treated with scrambled siRNA or with siRab11a+b, as in **Fig 6**. Cells were exposed to Tfn-AF488 for 30 min at 37°C, stained with TR-phalloidin/DAPI, and imaged by confocal microscopy. Representative images are shown. Bar = 5 µm. **(B) Simultaneous silencing of Rab11a and Rab11b diminishes the area of perinuclear Tfn-positive recycling endosomes.** The experiment was performed as in panel A and analyzed for the Tfn-positive recycling endosomal (RE) punctum, as described in **Fig. 3A.** Arrows point towards Tfn positive perinuclear endosomal puncta. Results are mean ± SE; n≥85 cells were analyzed in three independent experiments. Bar = 5 µm. **(C) Simultaneous silencing of Rab11a and Rab11b increases Tfn endocytic turnover.** HeLa cells treated with scrambled siRNA or siRab11a+b and exposed to Tfn-AF647. Tfn endocytosis and recycling was measured by flow cytometry. Results are mean ± SE; n≥12 were analyzed in three independent experiments.

S12 Fig. EspF and Map mediate the recruitment of Tfn/TfnR and Myo5b/Rab11a at infection sites. **(A) EspF and Map are essential for Tfn/TfnR recruitment at infection sites.** HeLa cells were infected with the EPEC-*espF* or *map* mutant strains and their corresponding *espF*+EspF, or *ma*p+Map complemented strains. Effector protein expression was induced, or not, by IPTG. Cells were exposed to Tfn-AF647 during infection, fixed, immunolabeled with anti-TfnR antibodies, stained with TR-phalloidin and DAPI and analyzed by confocal microscopy. Representative images of F-actin, TfnR and Tfn recruitment at infection sites are shown. Results are mean ± SE of n≥30 bacterial microcolonies analyzed in three independent experiments. Arrows point towards infecting microcolonies. Bar = 5 µm. **(B) EspF and Map are essential for Myo5b recruitment at infection sites.** HeLa cells were infected with the indicated EPEC strains. Effector protein expression was induced, or not, by IPTG. Cells were immunolabeled with anti-Myo5b and anti-Rab11a antibodies, followed by TR-phalloidin and DAPI staining, and analyzed by confocal microscopy. Representative images of the host cell markers at infection sites are shown. Results are mean ± SE; n≥30 infecting microcolonies analyzed in three independent experiments. Arrows point towards cell-adhered microcolonies. Bar = 5 µm. **(C) EspF and Map partially colocalize with Rab11a and Myo5b at infection sites.** HeLa cells were infected with the indicated EPEC-*espF* or EPEC-*map* complemented with EspF-Flag, or HA-Map encoding plasmids (**S3 Table**). Effector protein expression was induced by IPTG. Cells were immunolabeled with anti-Myo5b, anti-Rab11a, anti-Flag (EspF), anti-HA (Map) antibodies (**S4 Table**), stained with DAPI and analyzed by confocal microscopy. Boxed regions show immunstained bacterial effectors which partially overlapped with the recycling endocytic proteins, Rab11a and Myo5b, at infection sites. Bar = 5 µm. **(D) Simultaneous mutation of the *espF* and *map* genes abrogated the recruitment of Tfn/TfnR, Myo5b and Rab11a at infection sites.** HeLa cells were infected with the EPEC-espF/map mutant strain and analyzed as in panels A or B. Arrows point towards cell-adhered microcolonies. Bar = 5 µm.

S13 Fig. EspF and Map translocation and localization in the host. **(A) EspF and Map are translocated into HeLa cells.** HeLa cells were infected with the indicted EPEC strains (**S2 Table**), and translocated effectors were detected by Western blotting, using anti-Flag (EspF), anti-HA (Map) and anti-β-actin antibodies. Representative Western blots are shown. **(B) Translocated EspF and Map can be detected in mitochondria free zones at infection sites.** HeLa cells were infected with the EspF or Map complemented strains. Cells were fixed and immunolabeled with anti-HSP60 (mitochondria) and anti-Flag (EspF) or anti-HA (Map) antibodies. Cells were subsequently stained with TR-phalloidin/DAPI and analyzed by confocal microscopy. Representative images are shown. Arrows point towards effectors localized at mitochondria-free sites beneath the adhered bacterial microcolony. Bar = 5 µm.

S14 Fig. Tfn/iron-dependent increase of host cell surface colonization. Polarized MDCK-PTR9 or plain MDCK cells were infected with the contact-induced NleA-GFP expressing bacteria **(**EPEC*nleA-gfp*) and concomitantly treated with Tfn-AF647 (apical or basal), or with ferric nitrate (“Free iron”), or remained untreated. Cells were fixed, permeabilized and stained with DAPI/TR-phalloidin and imaged by confocal microscopy. The cell surface coverage by infecting (GFP expressing) bacteria **(A)** and the area of individual microcolonies **(B)** were determined. Notably, the degree of host cell surface coverage can be affected by bacterial microcolony size as well as by the number of microcolonies. Results are mean ± SE; n≥500 microcolonies analyzed in three independent experiments.

S15 Fig. T3SS-dependent recruitment of aquaporins 2 and 3 to infection sites. Polarized Caco2-BBe cells were infected with EPEC-*escV*, or EPEC-*wt* and immunolabeled with anti-aquaporin2 (AQP2) or anti-aquaporin 3 (AQP3) antibodies. These cells were also immunolabeled with and anti-Myo5b antibodies, and stained with TR-phalloidin and DAPI. Cells were subjected to confocal imaging and representative images are shown. Boxed regions highlight areas of Myo5b and aquaporin co-residence at infection sites. Arrows point towards infecting microcolonies. Bar = 5 µm.

**Supporting Tables**

**S1 Table: EPEC Strains**

| **Strain Name** | **Description** | **Reference and Comments** |
| --- | --- | --- |
| E2348/69 (*wt*) | EPEC isolate, serotype O127:H6 | Wild-type strain; [[3](#_ENREF_3)] |
| SN191 (*escV*) | *escV*::miniTn5*kan* | T3SS deficient mutant; [[4](#_ENREF_4)] |
| ME2018 (*cesT*) | ∆*cesT::Kn* | LEE chaperone; [[5](#_ENREF_5)] |
| ICC202 (*map*) | ∆*map::Kn* | LEE effector; Map, [[6](#_ENREF_6)] |
| XT111 (*espH*) | ∆*espH::Kn* | LEE effector; EspH, [[7](#_ENREF_7)] |
| UMD874 (*espF*) | ∆*espF::Kn* | LEE effector; EspF, [[8](#_ENREF_8)] |
| SE1207 (*espG1/G2*) | ∆*espG1/*∆*espG2* | LEE effector; EspG1/EspG2, [[9](#_ENREF_9)] |
| EM3458 (*tir*) | ∆*tir* | LEE effector; Tir, [[10](#_ENREF_10)] |
| XC2168 (*eae*) | ∆*eae::Kn* | LEE effector; Intimin, [[10](#_ENREF_10)] |
| EM3321 (*3321*) | ∆pp2::*kan*(∆*nleH1, espJ,cif psuedogene::kan*) | Non-LEE pathogenicity island, [[11](#_ENREF_11)] |
| EM3325 (*3325*) | ∆pp4::*kan* (∆*nleG2, nleB, nleC, nleH fragment, nleD::kan*) | Non-LEE pathogenicity island, [[11](#_ENREF_11)] |
| EM3331 (*3331*) | ∆pp6::*kan* (∆*nleH2, nleA.espI, nleF, espO fragment::kan*) | Non-LEE pathogenicity island, [[11](#_ENREF_11)] |
| EM3345 (*3345*) | ∆IE5*::kan* (∆*espG2, espC::kn*) | Non-LEE pathogenicity island, [[11](#_ENREF_11)] |
| EM3347 (*3347*) | ∆IE6*::kan* (∆*lifA, efa1, nleE, nleB, espL::kn*) | Non-LEE pathogenicity island, [[11](#_ENREF_11)] |
| RP8151 (*espF/map*) | E2348/69 Δ*espF*::kan, Δ*map*::cam | This study. |
| EPEC*nleA-gfp* (EM4620) | E2348/69 containing integrated pEM4617 (p*nleA-gfp*) | Non-LEE effector; [[2](#_ENREF_2)] |

**S2 Table: Reagents**

| **Reagent** | **Description** | **Source/Identifies** |
| --- | --- | --- |
| **Tissue Culture Reagents** | | |
| MEM | Minimum Essential Medium (MEM), with Earle’s Salts, Phenol Red and Sodium Bicarbonate but without L-Glutamine | Biological Industries, Beit Ha’emek, Israel. #01-025-1A |
| DMEM | Dulbecco's Modified Eagle Medium (DMEM), with no L-Glutamine, no Sodium Pyruvate | Biological Industries, Beit Ha’emek, Israel. #01-055-1A |
| DMEM without Phenol Red | DMEM with no L-Glutamine, no Sodium Pyruvate and no Phenol Red | Biological Industries, Beit Ha’emek, Israel. #01-053-1A |
| FBS | European Grade Fetal Bovine Serum (FBS) sourced in South America | Biological Industries, Beit Ha’emek, Israel. #04-007-1A |
| BSA | Bovine Serum Albumin (BSA) Solution (10%) | Biological Industries, Beit Ha’emek, Israel. #03-010-1B |
| Trypsin A | Trypsin EDTA Solution A | Biological Industries, Beit Ha’emek, Israel. #03-050-1A |
| Trypsin C | Trypsin EDTA Solution C | Biological Industries, Beit Ha’emek, Israel. #03-053-1A |
| DPBS | Dulbecco's Phosphate Buffered Saline (DPBS), no calcium, no magnesium | Biological Industries, Beit Ha’emek, Israel. #02-023-1A |
| Geneticin (G418 Sulfate) | Selective Antibiotic - Binds to the ribosome and inhibits protein synthesis | Gibco, Life Technologies, Paisley, UK. #11811-031 |
| Lipofectamin 2000 | Transfection Reagent | ThermoFisher Scientific, CA. #11668019 |
| TransIT-X2 6000 | Transfection Reagent | Mirus, Madison, WI. #Mir 6000, |
| **siRNA** | | |
| Scramble siRNA | siGENOME Non-Targeting siRNA Pool #2 | Dharmacon.  #D-001206-14-05 |
| si-Rab11a | siGENOME siRNA reagent targeting the human Rab11a | Dharmacon.  #M-004726-02-0005 |
| si-Rab11b | siGENOME siRNA reagent targeting the human Rab11b | Dharmacon.  #M-004727-02-0005 |
| **Fluorescent Reagents** | | |
| Tfn-AF488 | AlexaFluor (AF)-488 tagged human Transferrin | Jackson Immunoresearch Labs, West Grove, PA. #009-540-050 |
| Tfn-AF647 | AlexaFluor (AF)-647 tagged human Transferrin | Jackson Immunoresearch Labs, West Grove, PA. #009-600-050 |
| Tfn-DL649 | DyLight (DL)-649 tagged human Transferrin | Jackson Immunoresearch Labs, West Grove, PA. #009-490-050 |
| Propidium Iodide (PI) | Cell viability marker | Sigma Aldrich, St. Louis, MO. #P4170 |
| Texas-Red (TR)-Dextran | 70 kDa, Texas Red tagged Dextran | ThermoFisher Scientific. #D-1864 |
| Texas-Red (TR)- phalloidin | Texas-Red-tagged filamentous actin labeling reagent | Invitrogen, Eugene, Oregon. #T7451 |
| DAPI | 4’,6-Diamidine-2’-phenylindole dihydrochloride, DNA labeling reagent | Sigma Aldrich,St. Louis, MO. #D9542 |
| LysoTracker | LysoTracker™ Deep Red | ThermoFisher Scientific. #L12492 |
| **Other Reagents** | | |
| Holo-Tfn | Iron (Fe) Loaded human Transferrin | Biological Industries, Beit Ha’emek, Israel. #41-952-100 |
| Tfn-HRP | Horseradish Peroxidase (HRP)-tagged human Transferrin | Jackson Immunoresearch Labs, West Grove, PA. #009-030-050 |
| IPTG | Isopropyl β-D-1-thiogalactopyranoside | Promega, Madison, WI. #V395D |
| Deferroxamine mesylate | Iron chelator | Sigma Aldrich, St. Louis, MO. #D9533 |
| Triton X-100 | alkylaryl polyether alcohol  Non-ionic detergent | J.T. Baker, Phillipsburg, NJ. #X198-07 |
| MES | 2-(N-morpholino)ethanesulfonic acid | Sigma Aldrich, Steinheim, Germany. #M8250 |
| Protease inhibitor cocktail | Mammalian protease inhibitor cocktail. | Sigma Aldrich, St. Louis, MO. #P8340 |
| Phosphatase inhibitor cocktail | Mammalian phosphatase inhibitor cocktail. | Sigma Aldrich, St. Louis, MO. #P2850 |
| NaVO_4_ | Sodium orthovanadate, phosphatase inhibitor | Sigma Aldrich, St. Louis MO. #S6508 |
| Dynasore | Dynasore hydrate; noncompetitive dynamin 1 and dynamin 2 GTPase activity inhibitor | Sigma Aldrich, St. Louis MO. #D7693 [[12](#_ENREF_12)] |
| Dyngo | Dyngo 4a; a highly potent dynamin inhibitor and Dynasore analog | abcam. #ab120689 [[13](#_ENREF_13)] |

**S3 Table: Expression Constructs**

| **Plasmid name** | **Description** | **Source/ Reference** |
| --- | --- | --- |
| **Bacterial expression constructs** | | |
| pJN61-EspF | A pTrc99A-based vector containing FLAG-tagged espF (EPEC O127:H6 E2348/69) | Michael Donnenberg; [[8](#_ENREF_8)] |
| EspF-mod-wt | pTM007: a pTrc99A-based modular (mod) plasmid that contains two copies of the second proline rich region (PRR) and the native third PRR of EspF | Michael Donnenberg; [[14](#_ENREF_14)] |
| EspF-mod-LA | pKMS13: pTM007 that carries a point mutation on each N-WASP interacting modules (L31A, [[15](#_ENREF_15)]) | Michael Donnenberg;  [[14](#_ENREF_14)] |
| EspF-mod-RD | pKMS12: pTM007 that carries a point mutation on each of the SNX9 interacting modules (R3D, [[16](#_ENREF_16)]) | Michael Donnenberg;  [[14](#_ENREF_14)] |
| pSA10-Map | pSA10 derivative encoding HA-tagged Map (EPEC O127:H6 E2348/69) | Gad Frankel; [[17](#_ENREF_17)] |
| Map-ΔTRL | pSA10-*map*_ΔTRL_: Map whose C-terminal TRL PDZ type I binding motif (aa 608-612) has been deleted | Gad Frankel; [[18](#_ENREF_18)] |
| Map- WA/EA | pSA10-*map*_AxxxA_: Map containing W74A/E78A mutations which disrupt its GTPase binding | Gad Frankel; [[19](#_ENREF_19)] |
| pKD46 | λ RED genes, Amp^r^ | [[20](#_ENREF_20)] |
| pKD3 | Template for the chloramphenicol resistance cassette, Cam^r^ | [[20](#_ENREF_20)] |
| **Mammalian expression constructs** | | |
| mRFP-LifeAct | pCMV/pCAG-LifeAct fused to TagRFP; Labels the actin cytoskeleton | Ibidi. #60102 |
| GFP-LifeAct | pCMV/pCAG-LifeAct fused to TagGFP2; Labels the actin cytoskeleton | Ibidi. #60101 |
| GFP-Rab5a | GFP fused to human Rab5a | Marino Zerial; [[21](#_ENREF_21)] |
| mRFP-EEA1 | TagRFP-T fused to Early Endosome Antigen 1 (EEA1) | Addgene. #42635 |
| Human GFP-TfnR | GFP fused to the N-terminus of the human transferrin receptor | Enrique Rodriguez-Boulan; [[22](#_ENREF_22)] |
| GFP-SH3BP4 | eGFP fused to SH3 binding protein 4 (SH3BP4), also called TTP, which directly binds the TfnR and regulates its CME | Pier Paolo Di Fiore; [[23](#_ENREF_23)] |
| GFP-ACAP1 | eGFP fused to Arf6 GTPase-activating proteins 1 (ACAP1) shown to interact with the TfnR and promote its transport from recycling endosomes | Victor W. Hsu; [[24](#_ENREF_24)] |
| GFP-Rab11a | GFP fused to human Rab11a | James Goldenring; [[25](#_ENREF_25)] |
| mCherry- Rab11a | mCherry fused to human Rab11a | James Goldenring; [[26](#_ENREF_26)] |
| tdEos-Rab11a-7 | tdEos fused to human Rab11a-7 | Addgene. #57664 |
| Myo5b-FL | GFP fused to human Myosin 5b full length (FL) | James Goldenring; [[27](#_ENREF_27)] |
| Myo5b-tail | GFP fused to human Myosin 5b with C-terminal deletion (tail) | James Goldenring; [[27](#_ENREF_27)] |
| Myo5b-tail-QLYC | GFP fused to human Myosin 5b with C-terminal deletion (tail) bearing two point mutations (QLYC) | James Goldenring; [[28](#_ENREF_28)] |
| Myo5b-tail-YEQR | GFP fused to human Myosin 5b with C-terminal deletion (tail) bearing two point mutations (YEQR) | James Goldenring; [[28](#_ENREF_28)] |

**S4 Table: Antibodies**

| **Name** | **Description/Identifier** | **Dilution** |
| --- | --- | --- |
| **Primary antibodies** | | |
| Rabbit anti-CHC | Rabbit polyclonal anti-clathrin heavy chain; abcam. #ab21679 | IF; 1: 1000 |
| Mouse anti-AP2-α | Supernatant produced from the AP6 hybridoma; Detects assembly polypeptide 2 α (AP2-α); [[29](#_ENREF_29)] | IF; 1:200 |
| Mouse anti-Rab5a | Monoclonal anti-Rab5a;  BD Transduction Laboratories, #610724 | IF; 1:100 |
| Mouse anti-EEA1 | Monoclonal anti-EEA1;  BD Transduction Laboratories, #610456 | IF; 1:100 |
| Mouse anti-TfnR (H68.4) | Monoclonal antibody reacts with the N-terminal region of the human transferrin receptor; [[30](#_ENREF_30)] | IF; 1:2500 |
| Mouse anti-TfnR ectodomain (B3/25) | Mouse monoclonal directed against the human receptor ectodomain, Santa Cruz Biotechnology, #sc-65877. [[31](#_ENREF_31)] | IF; 1:50 |
| Rabbit anti-Rab11a | VU57; James Goldenring. [[32](#_ENREF_32)] | IF; 1:200, WB; 1:500 |
| Chicken anti-Myo5b | Prof. James Goldenring. [[33](#_ENREF_33), [34](#_ENREF_34)] | IF; 1:300 |
| Rabbit anti-FIP2 | Polyclonal anti Rab11-FIP2; Sigma Aldrich, #HPA037726 | IF; 1:200 |
| Rabbit anti-Rab25 | Polyclonal anti RAB25; Sigma Aldrich, #HPA010872 | IF; 1:200 |
| Rabbit anti-Rab11b | Monoclonal anti-RAB11B [EPR12725]; abcam, #ab175925 | IF; 1:100, WB; 1:500 |
| Rabbit anti-Rab7a | Monoclonal anti-Rab7a; abcam, #ab137029 | IF; 1: 100 |
| Rat anti-β1-integrin | Rat monoclonal anti-β1-integrin (AIIB2; [[35](#_ENREF_35)]) | IF; 1:1000 |
| Rabbit anti-Rab4a | Polyclonal anti RAB4a; abcam, #ab13252 | IF; 1:100 |
| Rabbit anti-human Tfn | Polyclonal anti-Transferrin; Dako, #A-0061 | WB; 1:2000 |
| Mouse anti-Flag tag | Monoclonal anti-Flag M2; Sigma Aldrich, #F3165 | IF; 1:500, WB; 1:1000 |
| Mouse anti-HA tag | Mouse monoclonal antibody (clone 12CA5) directed against the 9-amino acid sequence derived from the influenza hamagglutinin (HA) protein. abcam, #ab16918 | IF; 1:500, WB; 1:500 |
| Mouse anti-α-tubulin | Monoclonal anti-Tubulin-α antibody, Clone B512, Sigma-Aldrich, #T6074 | WB; 1:2000 |
| Mouse anti-β-actin | Monoclonal Anti-β-Actin Clone AC-15, Sigma Aldrich, #A5441 | WB; 1:5000 |
| Goat anti-Hsp60 | Polyclonal anti HSP60 (k-19); Santa Cruz Biotechnology, #ScL1722; | IF; 1:200 |
| Rabbit anti-AQP2 | Polyclonal anti aquaporin 2; Alomone labs, #AQP-002 | IF; 1:50 |
| Rabbit anti-AQP3 | Polyclonal anti aquaporin 3; Alomone labs, #AQP-003 | IF; 1:50 |
| Rabbit anti-SNX9 | monoclonal anti SH3PX1/SNX9; abcam, ab181856 | WB: 1:2000 |
| Rabbit anti- SNX18 | Polyclonal anti SNX18; abcam, ab111702 | WB: 1:2000 |
| Rabbit anti- SNX33 | Polyclonal anti SNX33; abcam, ab241201 | WB: 1:400 |
| **Secondary antibody** | | |
| Goat anti-mouse IgG, Alexa Fluor 488 | Alexa Fluor 488- AffiniPure Goat Anti-Mouse IgG; Jackson ImmunoResearch Laboratories, #115-545-062 | IF; 1:300 |
| Donkey anti-mouse IgG, Alexa Fluor 488 | Alexa Fluor 488- AffiniPure Goat Anti-Mouse IgG; Thermo-Fisher Scientific, #A-21202 | IF; 1:300 |
| Donkey anti-mouse IgG, Alexa Fluor 594 | Alexa Fluor-594- AffiniPure Donkey Anti-Mouse IgG; Jackson ImmunoResearch Laboratories, #715-585-151 | IF; 1:500 |
| Donkey anti-mouse IgG, Cy5 | Cy5- AffiniPure Donkey Anti-Mouse IgG; Jackson ImmunoResearch Laboratories, #715-175-151 | IF; 1:250 |
| Goat anti-rabbit IgG, Alexa Fluor 488 | Cross-Adsorbed anti rabbit IgG; Alexa Fluor 488; Thermo-Fisher Scientific, #A-11008 | IF; 1:300 |
| Donkey anti-rabbit IgG, Alexa Fluor 488 | Highly cross-Adsorbed anti rabbit IgG; Alexa Fluor 488; Thermo-Fisher Scientific, #A-21206 | IF; 1:300 |
| Donkey anti-rabbit IgG, Alexa Fluor 647 | Alexa Fluor-647- AffiniPure Donkey Anti-rabbit IgG; Jackson ImmunoResearch Laboratories, #711-602-152 | IF; 1:300 |
| Donkey anti-rat IgG, Cy3 | Cy3- AffiniPure Donkey Anti-rat IgG; Jackson ImmunoResearch Laboratories, #712-165-153 | IF; 1:250 |
| Donkey anti-rat IgG, Cy5 | Cy5- AffiniPure Donkey Anti-rat IgG; Jackson ImmunoResearch Laboratories, #712-175-153 | IF; 1:250 |
| Donkey anti-goat IgG, Alexa Fluor 488 | Cross-Adsorbed anti goat IgG; Alexa Fluor 488; Thermo-Fisher Scientific, #A-11055 | IF; 1:300 |
| Donkey anti-goat IgG, Alexa Fluor 647 | Cross-Adsorbed anti goat IgG; Alexa Fluor 647; Thermo-Fisher Scientific, #A-21447 | IF; 1:100 |
| Peroxidase goat anti-mouse IgG | Jackson ImmunoResearch Laboratories, #115-035-166 | WB; 1:10000 |
| Peroxidase goat anti- rabbit IgG | Jackson ImmunoResearch Laboratories, #111-035-003 | WB; 1:10000 |

**S5 Table: Primers**

| **Numbers** | **Sequences (5’-3’)** | **Usages** |
| --- | --- | --- |
| 1354 | GCAATGGTAGGTAGAGCGTTAGCTCAGGCGGTTACACAAACTCTTAGACCCGTGTAGGCTGGAGCTGCTTC | Creation of RP8151 |
| 1355 | CTGCAATCGCCTTCTCAGTTAGAGCCTTGATATCACTGATTTTCGCGGTGCCATATGAATATCCTCCTTAG | Creation of RP8151 |
| 1371 | GTATCCACTCATGACCATCG | Creation of RP8151 |
| 1495 | CTCTGGAATCGACAGAATCAGCC | Creation of RP8151 |
| 4197 | GAGCTAACGCTCTACCTACC | Creation of RP8151 |
| 4198 | CTAACTGAGAAGGCGATTGC | Creation of RP8151 |
| 115 | GATCTTCCGTCACAGGTAGG | Verification of RP8151. |
| 1682 | GCTAAACCAGCAGCAATTGCG | Verification of RP8151 |

# Supporting Movies

**S1-S6 Movies** were generated from cells imaged with time-lapse confocal imaging at 2 min per frame intervals. Fluorescence signals from GFP, mRFP and DL649 are presented as green, red and blue respectively. **S7-S8** **Movies** were generated from cells imaged with time-lapse confocal imaging at 0 min per frame intervals (i.e., next frame imaged as soon as the last one was completed, in this case, ~4 sec intervals). Fluorescence signals from tdEos-Rab11a, photoconverted tdEos-Rab11a and AF647 are presented as green, red and blue respectively. In all experiments, differential interference contrast (DIC) images were acquired to visualize cell-adhered bacteria (indicated by arrows).

**References:**

1. Ramachandran, R.P., et al., *EspH Suppresses Erk by Spatial Segregation from CD81 Tetraspanin Microdomains.* Infection and immunity, 2018. **86**(10).

2. Katsowich, N., et al., *Host cell attachment elicits posttranscriptional regulation in infecting enteropathogenic bacteria.* Science, 2017. **355**(6326): p. 735-739.

3. Levine, M.M., et al., *Escherichia coli strains that cause diarrhoea but do not produce heat-labile or heat-stable enterotoxins and are non-invasive.* Lancet, 1978. **1**(8074): p. 1119-22.

4. Nadler, C., et al., *Characterization of enteropathogenic Escherichia coli mutants that fail to disrupt host cell spreading and attachment to substratum.* Infect Immun, 2006. **74**(2): p. 839-49.

5. Li, M., et al., *Identification and characterization of NleI, a new non-LEE-encoded effector of enteropathogenic Escherichia coli (EPEC).* Microbes Infect, 2006. **8**(14-15): p. 2890-8.

6. Wong, A.R., et al., *The interplay between the Escherichia coli Rho guanine nucleotide exchange factor effectors and the mammalian RhoGEF inhibitor EspH.* mBio, 2012. **3**(1).

7. Tu, X., et al., *EspH, a new cytoskeleton-modulating effector of enterohaemorrhagic and enteropathogenic Escherichia coli.* Mol Microbiol, 2003. **47**(3): p. 595-606.

8. McNamara, B.P., et al., *Translocated EspF protein from enteropathogenic Escherichia coli disrupts host intestinal barrier function.* J Clin Invest, 2001. **107**(5): p. 621-9.

9. Elliott, S.J., et al., *EspG, a novel type III system-secreted protein from enteropathogenic Escherichia coli with similarities to VirA of Shigella flexneri.* Infection and immunity, 2001. **69**(6): p. 4027-33.

10. Mills, E., et al., *Dynamics of the type III secretion system activity of enteropathogenic Escherichia coli.* mBio, 2013. **4**(4).

11. Nadler, C., et al., *The type III secretion effector NleE inhibits NF-kappaB activation.* PLoS pathogens, 2010. **6**(1): p. e1000743.

12. Kirchhausen, T., E. Macia, and H.E. Pelish, *Use of dynasore, the small molecule inhibitor of dynamin, in the regulation of endocytosis.* Methods in enzymology, 2008. **438**: p. 77-93.

13. McCluskey, A., et al., *Building a better dynasore: the dyngo compounds potently inhibit dynamin and endocytosis.* Traffic, 2013. **14**(12): p. 1272-89.

14. Garber, J.J., et al., *Attaching-and-Effacing Pathogens Exploit Junction Regulatory Activities of N-WASP and SNX9 to Disrupt the Intestinal Barrier.* Cell Mol Gastroenterol Hepatol, 2017. **5**(3): p. 273-288.

15. Cheng, H.C., et al., *Structural mechanism of WASP activation by the enterohaemorrhagic E. coli effector EspF(U).* Nature, 2008. **454**(7207): p. 1009-13.

16. Alto, N.M., et al., *The type III effector EspF coordinates membrane trafficking by the spatiotemporal activation of two eukaryotic signaling pathways.* J Cell Biol, 2007. **178**(7): p. 1265-78.

17. Berger, C.N., et al., *EspZ of enteropathogenic and enterohemorrhagic Escherichia coli regulates type III secretion system protein translocation.* mBio, 2012. **3**(5): p. 00317-12.

18. Simpson, N., et al., *The enteropathogenic Escherichia coli type III secretion system effector Map binds EBP50/NHERF1: implication for cell signalling and diarrhoea.* Mol Microbiol, 2006. **60**(2): p. 349-63.

19. Berger, C.N., et al., *The mechanisms used by enteropathogenic Escherichia coli to control filopodia dynamics.* Cell Microbiol, 2009. **11**(2): p. 309-22.

20. Datsenko, K.A. and B.L. Wanner, *One-step inactivation of chromosomal genes in Escherichia coli K-12 using PCR products.* Proc Natl Acad Sci U S A, 2000. **97**(12): p. 6640-5.

21. Sonnichsen, B., et al., *Distinct membrane domains on endosomes in the recycling pathway visualized by multicolor imaging of Rab4, Rab5, and Rab11.* The Journal of cell biology, 2000. **149**(4): p. 901-14.

22. Perez Bay, A.E., et al., *The kinesin KIF16B mediates apical transcytosis of transferrin receptor in AP-1B-deficient epithelia.* The EMBO journal, 2013. **32**(15): p. 2125-39.

23. Tosoni, D., et al., *TTP specifically regulates the internalization of the transferrin receptor.* Cell, 2005. **123**(5): p. 875-88.

24. Li, J., et al., *An ACAP1-containing clathrin coat complex for endocytic recycling.* J Cell Biol, 2007. **178**(3): p. 453-64.

25. Casanova, J.E., et al., *Association of Rab25 and Rab11a with the apical recycling system of polarized Madin-Darby canine kidney cells.* Mol Biol Cell, 1999. **10**(1): p. 47-61.

26. Baetz, N.W. and J.R. Goldenring, *Rab11-family interacting proteins define spatially and temporally distinct regions within the dynamic Rab11a-dependent recycling system.* Molecular biology of the cell, 2013. **24**(5): p. 643-58.

27. Lapierre, L.A., et al., *Myosin vb is associated with plasma membrane recycling systems.* Molecular biology of the cell, 2001. **12**(6): p. 1843-57.

28. Roland, J.T., et al., *Rab GTPase-Myo5B complexes control membrane recycling and epithelial polarization.* Proceedings of the National Academy of Sciences of the United States of America, 2011. **108**(7): p. 2789-94.

29. Chin, D.J., et al., *100-kDa polypeptides in peripheral clathrin-coated vesicles are required for receptor-mediated endocytosis.* Proc Natl Acad Sci U S A, 1989. **86**(23): p. 9289-93.

30. White, S., et al., *Monoclonal antibodies against defined epitopes of the human transferrin receptor cytoplasmic tail.* Biochimica et biophysica acta, 1992. **1136**(1): p. 28-34.

31. Taetle, R., J. Castagnola, and J. Mendelsohn, *Mechanisms of growth inhibition by anti-transferrin receptor monoclonal antibodies.* Cancer research, 1986. **46**(4 Pt 1): p. 1759-63.

32. Lapierre, L.A., et al., *Characterization of immunoisolated human gastric parietal cells tubulovesicles: identification of regulators of apical recycling.* American journal of physiology. Gastrointestinal and liver physiology, 2007. **292**(5): p. G1249-62.

33. Lapierre, L.A., et al., *Phosphorylation of Rab11-FIP2 regulates polarity in MDCK cells.* Molecular biology of the cell, 2012. **23**(12): p. 2302-2318.

34. Schafer, J.C., et al., *Rab11-FIP2 interaction with MYO5B regulates movement of Rab11a-containing recycling vesicles.* Traffic, 2014. **15**(3): p. 292-308.

35. Hall, D.E., et al., *The alpha 1/beta 1 and alpha 6/beta 1 integrin heterodimers mediate cell attachment to distinct sites on laminin.* J Cell Biol, 1990. **110**(6): p. 2175-84.
